# Heterogeneous graphene oxide as recyclable catalyst for azomethine ylide mediated 1,3 dipolar cycloaddition reaction in aqueous medium†

**DOI:** 10.1039/c8ra06714g

**Published:** 2018-10-17

**Authors:** Marri Sameer Reddy, Nandigama Satish Kumar, L. Raju Chowhan

**Affiliations:** School of Chemical Sciences, Central University of Gujarat Sector 30 Gandhinagar-382030 India; Centre for Applied Chemistry, Central University of Gujarat Sector 30 Gandhinagar-382030 India rchowhan@cug.ac.in; Nanoscience and Nanotechnology Laboratory, Department of Chemistry, Gitam Institute of Sciences, Gitam University Visakhapatnam 530045 India

## Abstract

Graphene oxide (GO) catalysed multi component reaction of azomethine ylide driven 1,3 dipolar cycloaddition reaction in aqueous ethanolic solution is reported for the first time. This strategy has been applied for the synthesis of poly heterocyclic spiro-indenoquinoxaline pyrrolizidines and spiro-oxindoles pyrrolizidines with good to excellent yield along with excellent regio and diastereoselectivity. An ultra-low catalyst loading of 0.50 wt% was found to be efficient to catalyse the reaction.

## Abstract 2

Graphene oxide (GO) catalyzed regio and diastereoselective synthesis of spiro-indenoquinoxaline pyrrolizidines and spiro-oxindoles pyrrolizidines is described with good substrate scope and yield using azomethine ylide under aq. EtOH condition at RT.

## Graphical abstract

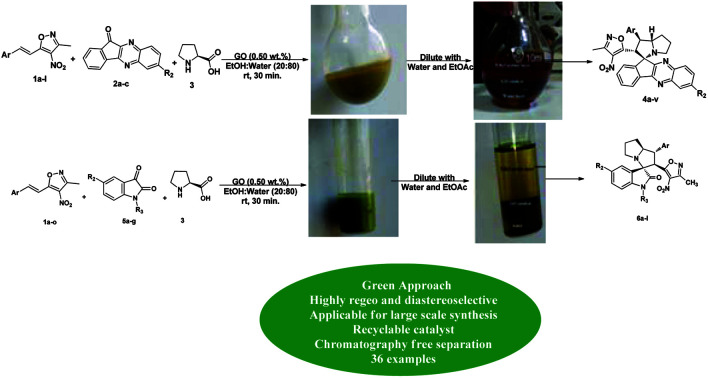

## 1. Introduction

**Fig. 1:**
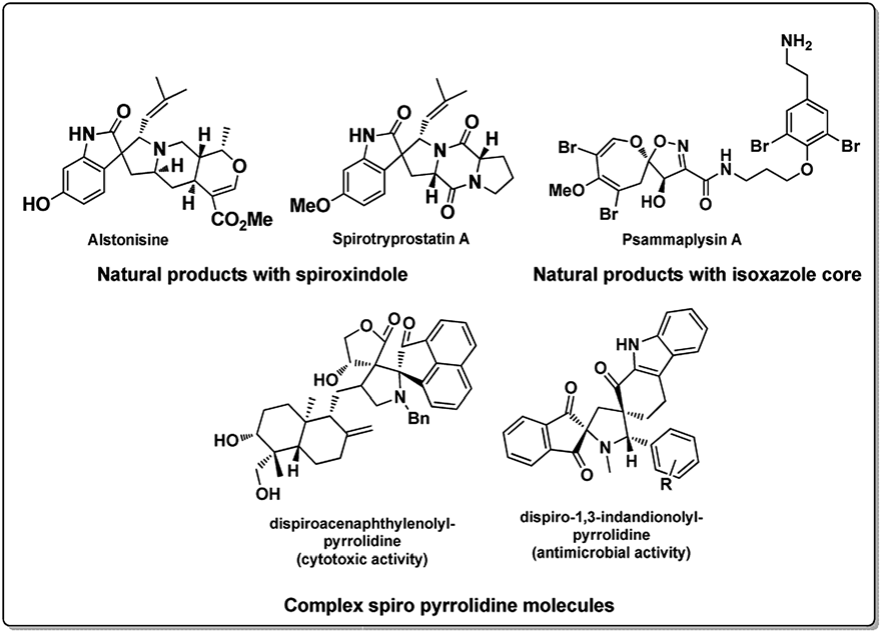
Some biologically potent spiro and isoxazole molecules.

Development of carbon based heterogeneous catalysts in recent times has opened new vistas in the areas of green and sustainable chemistry.^1^ The use of graphene and its derivatives like graphene oxide functionalized with various functionalities has been slowly and effectively found to replace transition metals in organic synthesis.^2^ Especially graphene oxide has been used successfully in an array of reactions ranging from Friedel craft alkylation,^3^ oxidative coupling reactions,^4^ selective hydrogen transfer,^5^ hydration oxidation,^6^ Diels–Alder reaction,^7^ allylic oxidation^8^ and many others.^9^ It is interesting to note that this economically viable heterogeneous catalyst graphene oxide (GO) has found application in catalysing MCR with high yields,^10^ especially when applied as a catalyst in aqueous media^11^ it is a promising lead in developing large scale application. On the other hand, the emergence of multicomponent reactions (MCR) has provided chemists an effective means of synthesizing complex, multifunctional moieties by overcoming complex synthetic procedures. They have proved not only to be efficient but also robust in operation, improving areas like drug discovery and material sciences over last few decades.^12^ Especially aqueous MCRs are promising strategies in the purview green chemistry.^13^ Moreover, MCR associated with 1,3 dipolar cycloaddition reactions (1,3 DCR) of azomethine ylides has been largely explored in recent time for the synthesis of pyrrolizidine and substituted pyrrole core which represent a large class of alkaloids (Fig. 1).^14^ This strategy has enabled the chemists to concise the synthesis of spirooxindoles which are one of the widely occurring spiro alkaloids^15^ along with a large synthetic analogue pool of spirooxindoles, with biological activities^16^ as well as few other natural products.^17^ However, it can be observed from the literature reports, many reactions suffer from longer reaction time, involves use of metal salts like silver or copper and volatile organic solvents.^12*a*^ There are very few reports in the literature on aqueous 1,3 dipolar cycloaddition reactions involving azomethine ylides is reported.^18^ We believe a robust, rapid, highly selective, green method for the synthesis of spiro molecules is of high value.

In our pursuit to develop multi component/green reactions,^19^ we herein report an MCR involving azomethine ylide in aqueous medium, catalysed by highly recyclable graphene oxide catalyst with ultra-low loading (0.5 wt%). Thus, developed method has been successfully applied for efficient synthesis of poly heterocyclic spiro pyrrolizidines molecules of oxindole and indenoquinoxaline using 3-methyl-4-nitro-5-alkenylisoxazoles as dipolarophile in aqueous medium. Products were isolated in high yields in analytically pure form without performing column chromatography. The strategy also explored for its recyclability of the catalyst (up to 4 cycle) and scale up experiment on gram scale (10 mmol) successfully. To best of our knowledge, this is the first report of graphene oxide (GO) catalysed azomethine ylide 1,3 dipolar cycloaddition reaction in aqueous medium.

## 2. Results and discussion

**Fig. 2:**
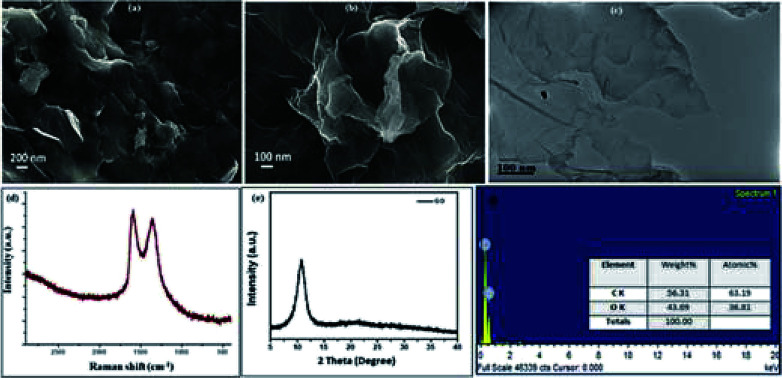
(a) and (b) SEM images with scale bar 200 nm and 100 nm; (c) TEM images with 100 nm scale bar; (d) Raman spectra; (e) powder XRD pattern; (f) EDX elemental analysis plot.

The GO catalyst was synthesized using improved Hummer's method^20^ and the structural and elemental investigation was done using IR, Raman, powder XRD, SEM, EDS, and TEM. The SEM, TEM images (Fig. 2a–f) establishes the sheet like structure of GO synthesized. Confirmation of formation of GO was done from PXRD with its characteristic value of 12.6*θ*. The SEM, TEM images (Fig. 2a–c) shows the multiple well segregated layers of GO. The Raman spectra shows characteristic peaks at 1609 and 1360 cm^−1^, confirms the same with clear.

**Scheme 1:**
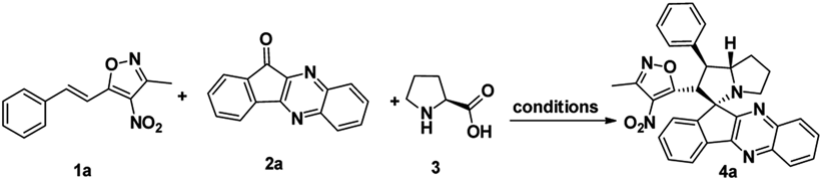
Synthesis of spiro indenoquinoxaline pyrrolizidine.

To proceed with the optimisation studies, we chose synthesis of novel spiro indenoquinoxaline pyrrolizidines by three components 1,3 DCR using 3-methyl-4-nitro-5-alkenylisoxazole 1a, indeno quinoxalinone (11*H*-indeno[1,2-*b*]quinoxalin-2-one) 2a and l-proline 3. Initially the reaction was performed at room temperature (RT) using ethanol as solvent, desired product was formed in very low yields even after 24 h (Table 1, entry 1) without any catalyst (Scheme 1). However, under reflux condition in ethanol yield was found to be increased substantially up to 60% yield after 4 h (Table 1, entry 2). On further prolong reflux, the yields lower and we observed un identified spots in TLC and we failed to identify them (Table 1, entry 3). Our major aim of the study was to perform the reaction at RT with high yields. Hence, we turned to explore catalytic reaction. To our delight, the desired product was formed with excellent regio and diastereoselectivity in 89% yield at RT in ethanol when 2 wt% of GO was employed as catalyst within 40 minutes (Table 1, entry 4). Encouraged with these results we proceeded with the optimisation study to check the lowering catalytic load from 2 wt% to 0.5 wt% and to our surprise much better yields were obtained (Table 1, entry 5). We then turned our focus to replace ethanol with water to make the reaction more sustainable. Replacing ethanol with water completely drastically reduced the yield (Table 1, entry 6). We assumed that the lower solubility of the substrates might be the reason for this result. So, further optimisations were planned in aqueous ethanolic solution and it can be seen that a ratio of ethanol to water in (2 : 8) was to be an efficient medium for the given reaction and proceeded (Table 1, entry 7 and 8) and also observations showed better conversion was seen with 1 eq. of indenoquinoxalinone 2, 1.2 eq. of proline 3 and 1.2 eq. of 3-methyl-4-nitro-5-alkenylisoxazoles 1 were used. Further reduction of catalytic loading was also found to be efficient but took longer time for the conversion of substrate into products (Table 1, entry 9). More importantly, in all the catalytic reaction, the products were isolated without column chromatography in analytically pure form, by simple dissolving the solid formed at the end of the reaction in ethyl acetate and filtering out the heterogenous catalyst followed by concentrating under vacuum or simply by washing crude solid with cold methanol.

**Table 1:** Optimisation of reaction

Entry^a^	Solvent	Catalytic loading (wt%)	Temperature	Reaction time	Yield^b^ (%)
1	EtOH	—	RT	24 h	20
2	EtOH	—	Reflux	4 h	60
3	EtOH	—	Reflux	10 h	40
4	EtOH	2	RT	40 min	89
5	EtOH	0.50	RT	30 min	95
6	H_2_O	0.50	RT	2 h	10
7	EtOH : H_2_O (1 : 9)	0.50	RT	3 h	82
8	EtOH : H_2_O (2 : 8)	0.50	RT	30 min	90^c^
9	EtOH : H_2_O (2 : 8)	0.20	RT	2 h	85^c^

a All reactions were carried out at 0.5 mmol scale with equimolar concentration.

b Isolated yield.

c With 1 eq. of indenoquinoxalinone, 1.2 eq. of proline and 1.2 eq. of 3-methyl-4-nitro-5-alkenylisoxazoles were used.

Under these optimised conditions we further explored the substrate scope and found the reaction conditions accommodated wide range of substrates both 3-methyl-4-nitro-5-alkenylisoxazoles 1a–l and indenoquinoxalinone 2a–c and the results were summarised in Table 2. From Table 2 it is learnt that substrate with halogens and alkyl groups performed well and afforded the desired product in good to excellent yield with high diastereoselectivity. The progress of the reaction can be visualised by change in colour of the reaction mixture as the reaction proceeds colour changes from brown to pale yellow precipitation at the end.

**Table 2:** Substrate scope for the synthesis of spiro indenoquinoxaline pyrrolizidine^a^

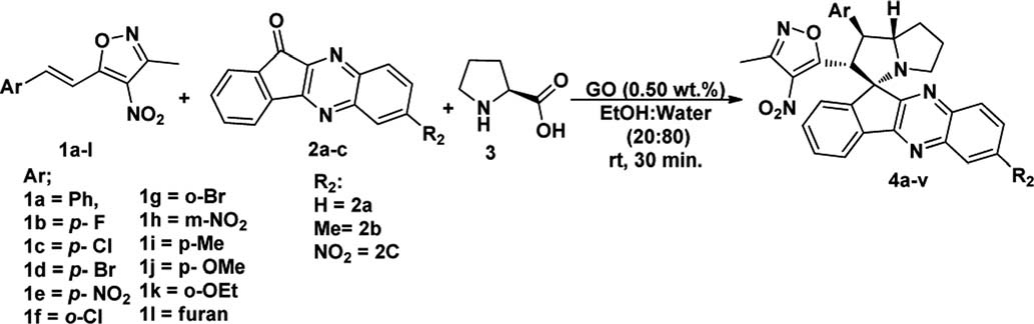
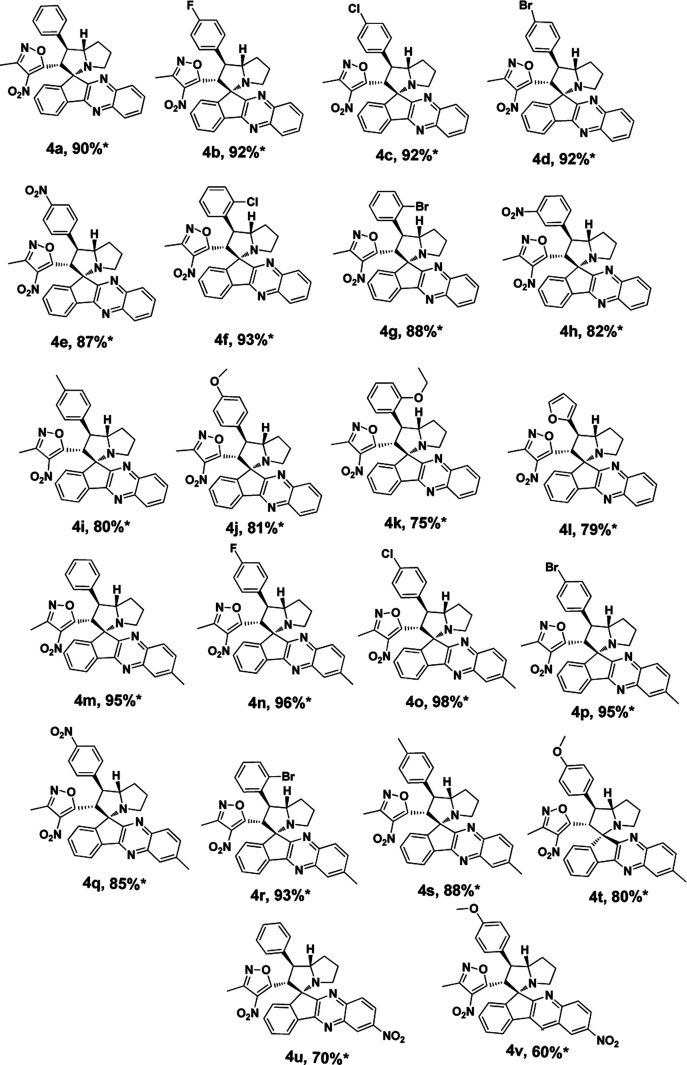

a Reaction conditions: all the reactions were carried out at 0.5 mmol scale with 1 eq. of indenoquinoxalinone, 1.2 eq. of proline and 1.2 eq. of 3-methyl-4-nitro-5-alkenylisoxazoles. *Isolated yield.

**Fig. 3:**
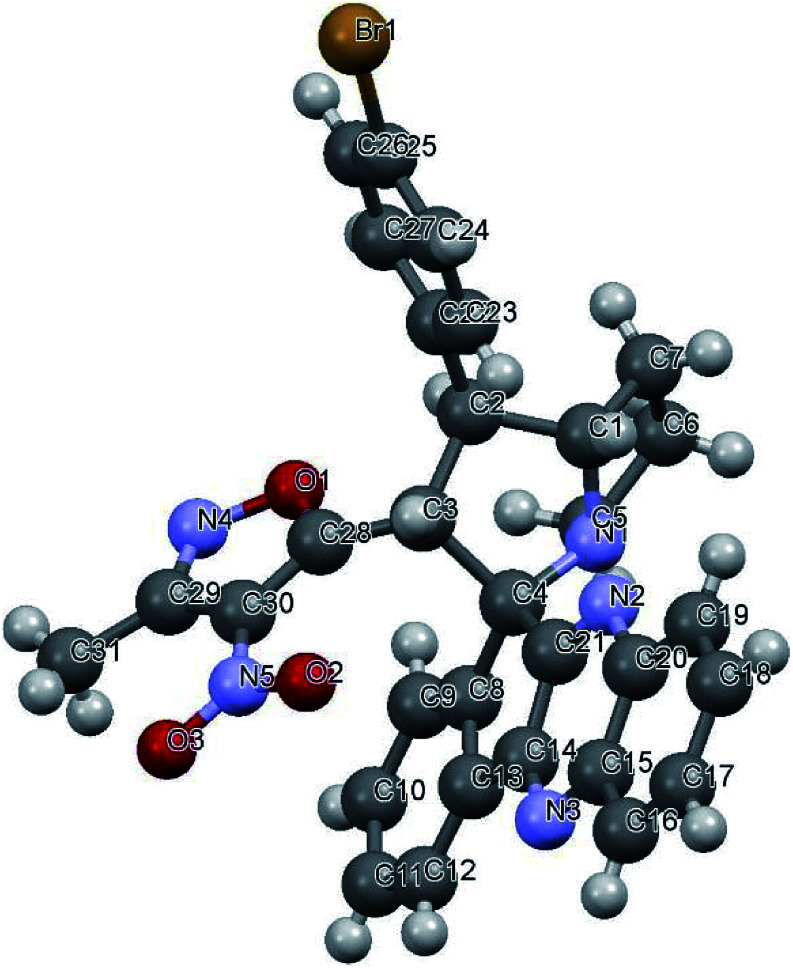
X-ray crystallographic structure of compound 4d (CCDC 1833739).

Variation in the yields obtained was found to be influenced by the substitutions on the substrates. Hetero aromatic furan ring (Table 2, 4l) was suffered from the lower yields. Also, in case of nitro substituted indenoquinoxalinone 2c afforded less yield (Table 2, 4u–v). However, nitro substituted 3-methyl-4-nitro-5-alkenylisoxazoles irrespective of the position has suffered from lower yields (4e, h, q). This lead us to assume that under given reaction condition, the reactivity of nitro substituted reactants is hindered due to deactivation of the aromatic ring and makes low reactive in the reaction. All the substrates afforded the desired products as a single regio isomer and diastereomers. Finally, the absolute structure was determined by single crystal X-ray diffraction analysis of 4d (Fig. 3).

**Fig. 4:**
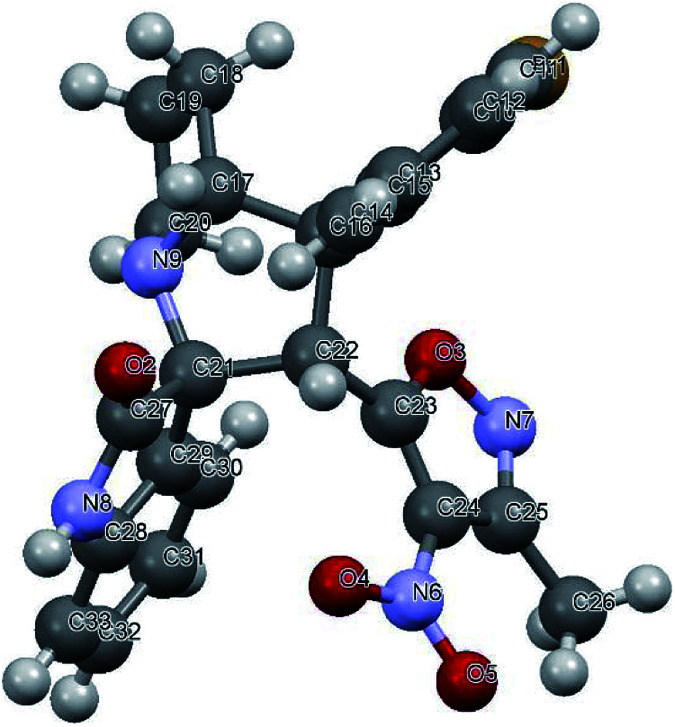
X-ray crystallographic structure of compound 6d (CCDC 1849303).

We also employed this strategy on various isatins 5a–g to increase the substrate scope of the methodology. In previous report, Liu *et al.* has reported the synthesis of isoxazole-fused spiro pyrrolizidine oxindoles in CH_3_CN solvent under reflux with a reaction time of 24 hours.^16*b*^ However, under the optimized reaction conditions in presence of GO catalyst, the reaction time were considerable reduced to 45 minutes with high regio and diastereoselectivity. A series of compounds were synthesised with good to excellent yield (Table 3) with good substrate scope. In this case also the progress of the reaction can be seen by change in the colour of the reaction mixture as the reaction proceeds the colour of the reaction mixture changes from orange to greenish precipitate. The absolute configuration was derived from single crystal X-ray diffraction analysis of 6d (Fig. 4).

**Table 3:** Substrate scope for synthesis of spiro pyrrolizidine oxindoles^a^

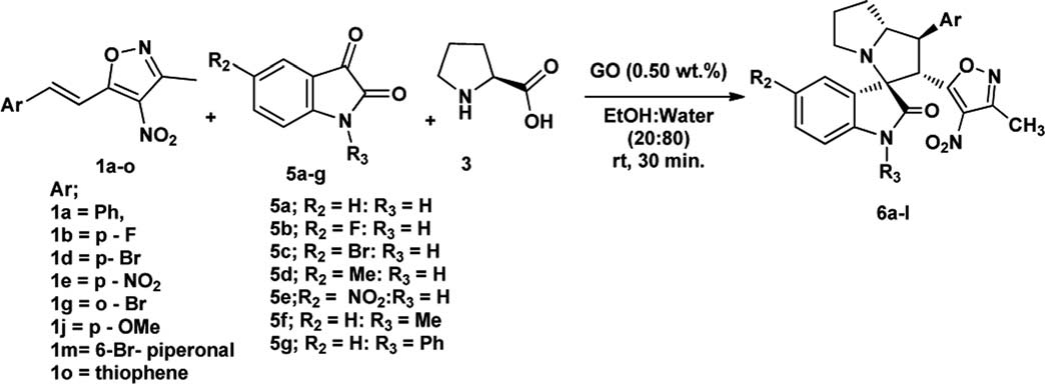
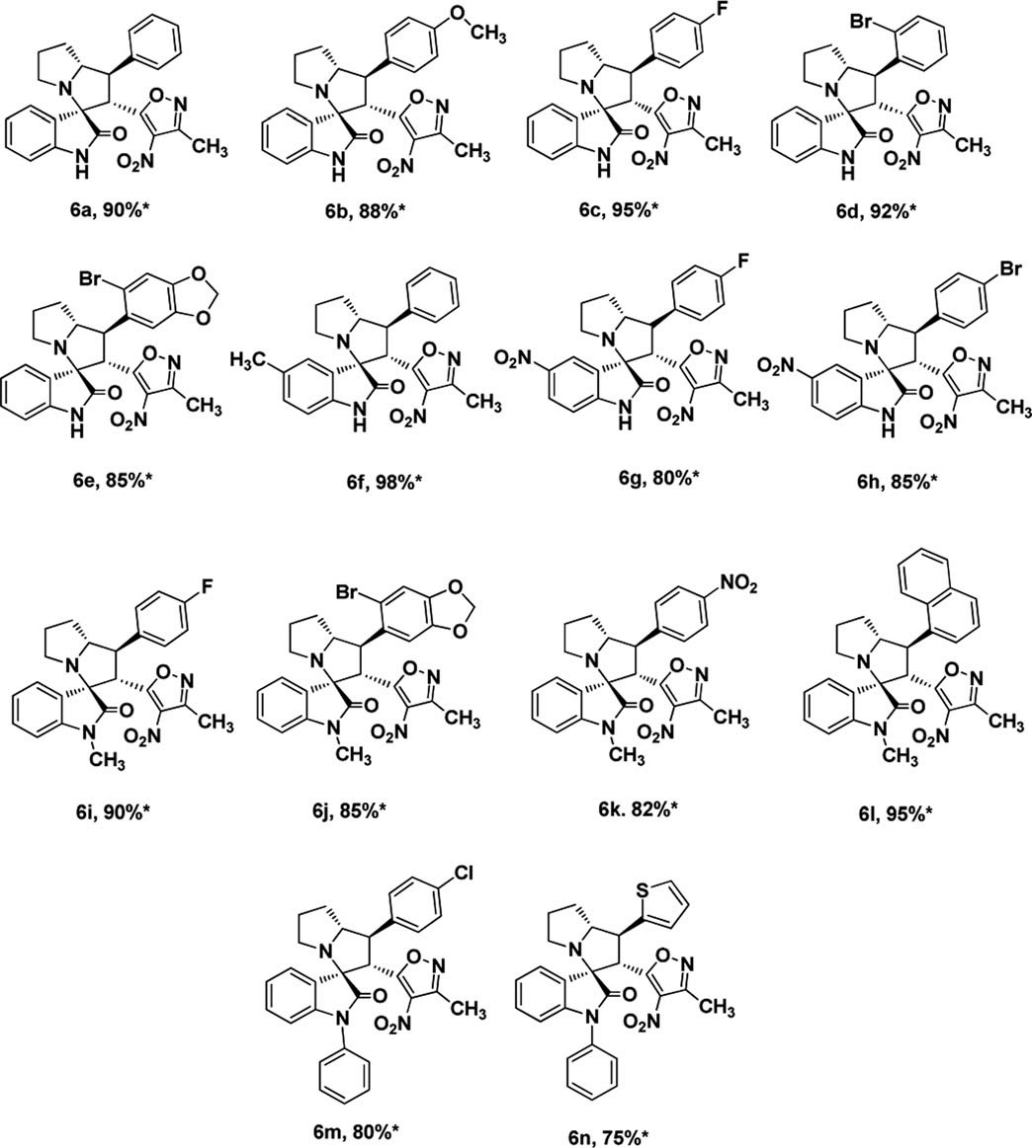

a Reaction conditions: all the reactions were carried out at 0.5 mmol scale with 1 eq. of isatin 5a–g, 1.2 eq. of proline 3 and 1.2 eq. of 3-methyl-4-nitro-5-alkenylisoxazoles 1a–o. *Isolated yield.

**Scheme 2:**
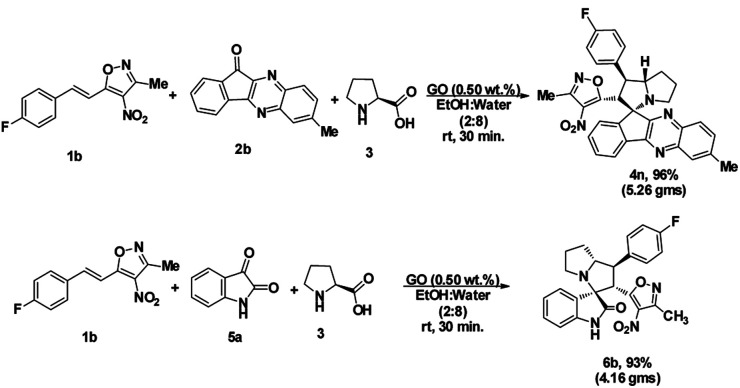
Scale-up experiment.

**Fig. 5:**
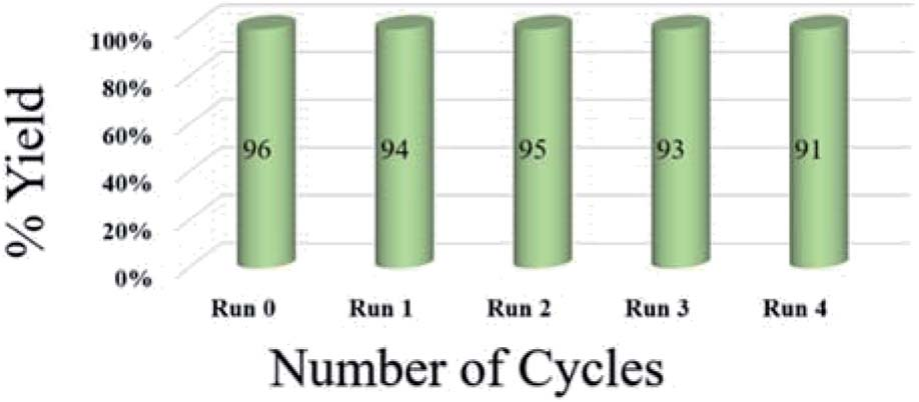
Recyclability experiment with 3-methyl-4-nitro-5-alkenylisoxazole 1b with indenoquinoxaline 2b and l-proline 3 as substrates at 10 mmol.

To assess the efficiency of the catalyst, we performed a scale up experiment using 3-methyl-4-nitro-5-alkenylisoxazole 1b with 2b and 1b with 5a as substrates on 10 mmol scale (Scheme 2). Both indenoquinoxalinone 2b and isatin 5a with 3-methyl-4-nitro-5-alkenylisoxazole 1a and l-proline 3 performed well in gram scale reaction. As the catalytic loading was very low, the recycling experiment was done at 10 mmol scale to obtain considerable amount of GO to recycle. At the end of the experiment GO was recovered by dissolving the solid product in ethyl acetate followed by centrifugation at 15 000 rpm for 2 hours GO was washed by repeating the process for five times to remove all the traces of organic compounds. After four successive cycles we observed there was no considerable change in yields at the end of 4^th^ catalytic cycle also proving the effectiveness and efficiency of the catalyst (Fig. 5).

## 3. Control experiment and plausible mechanism

**Fig. 6:**
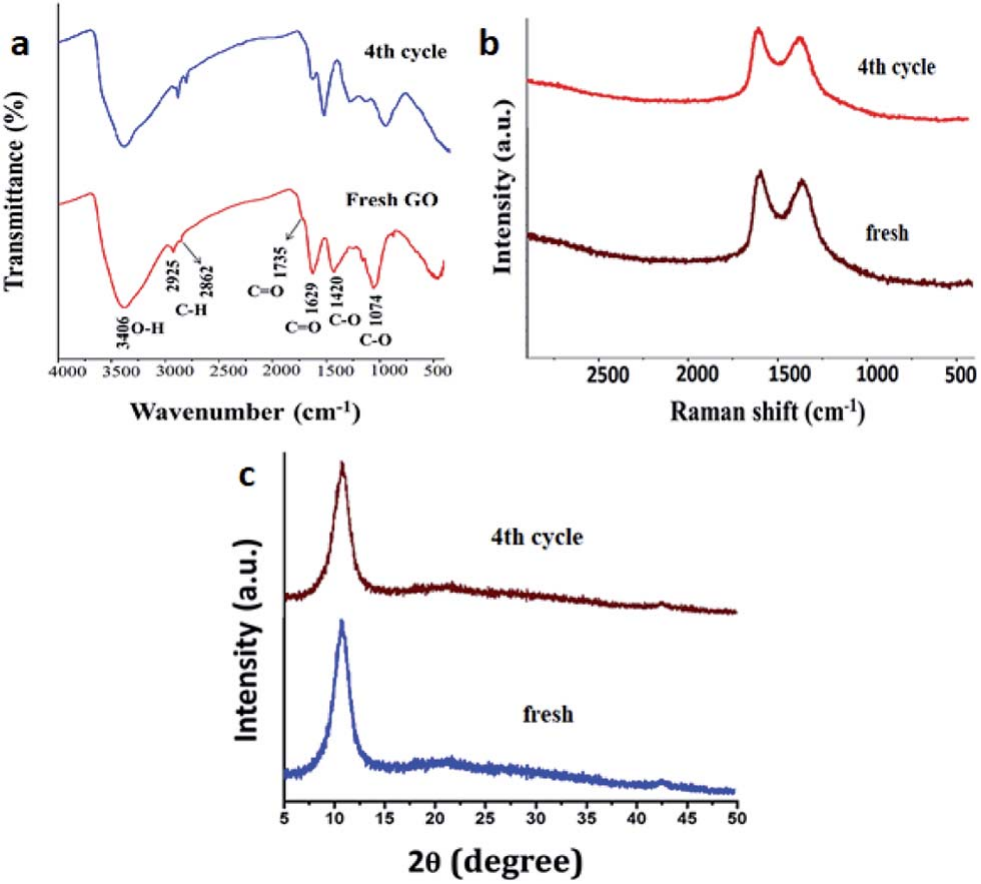
Characterization of fresh and reused after the catalytic cycle. (a) IR spectra (b) Raman spectra and (c) powder XRD.

To assess any structural changes in GO catalyst, the recovered catalyst at 4^th^ catalytic cycle was analysed with IR, Raman spectroscopy and powder XRD compared with the fresh sample of GO. There was not much change in the values in all the three analyses for both fresh and recycled GO. Especially, the characteristic peaks of –OH, –C=O, C–O stretching frequencies remained intact in IR spectroscopy and values of G and D band in Raman spectroscopy (1609 and 1360 cm^−1^ for fresh and 1593 and 1371 cm^−1^ for re-used respectively) confirms that there has been no change in surface functionalities which contributed to the catalytic efficiency of the GO (Fig. 6).

From previous reports and above observation, it is reasonable to assume the mechanism of the reaction as involves aromatic π-stacking interactions and hydrogen bonding are playing vital for the catalytic activity.^21^ As the reactants possess aromatic rings, the aromatic frame work with large surface area help in anchoring the substrates to GO surface *via* π-stacking. To confirm the involvement of hydrogen bonding of polar groups (both –OH and –COOH groups) in the catalysis, we performed some control experiments with other carbon materials. The effect can be seen from Table 4. 3-Methyl-4-nitro-5-alkenylisoxazole 1b with indenoquinoxaline 2b and l-proline 3 were used for the control experiments against various carbon catalysts.

**Table 4:** Control experiments with Carbon materials

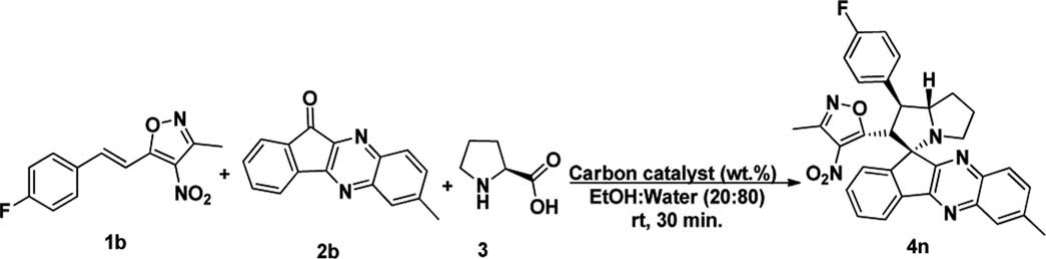				
Entry^a^	Carbon material (catalyst)	Quantity (wt%)	Time (h)	Yield^b^ (%)
1	GO	0.5	0.5	93
2	Reduced graphene oxide (rGO)	1.0	4.0	88
3	Graphite powder	1.0	24	50
4	HOPG	1.0	16	70
5	Activated charcoal	2.0	24	45

a Reaction conditions: all the reactions were carried out at 0.5 mmol scale with 1 eq. of 2b, 1.5 eq. of 3 and 1.2 eq. of 1b in ethanolic solution of ethanol to water in (2 : 8) ratio. All the catalysts were weighed and dispersed through sonication in the reaction solvent before conducting the experiment.

b Isolated yield. HOPG (Highly Oriented Pyrolytic Graphite).

GO with high polar surface functionalities along with large surface area has outperformed all other carbon catalysts in very low time and catalytic loading (Table 4, entry 1). On the other hand with lesser polar groups on its surface, rGO (reduced graphene oxide) also performed well but took longer reaction (Table 4, entry 2). HOPG (Highly Oriented Pyrolytic Graphite) performed well over commercially available graphite possibly due to higher surface area (Table 4, entry 3 and 4), but were found to be less efficient than GO catalyst. This observation proves that the π-stacking and secondary interactions are playing major role in the catalysis. Moreover, activated charcoal afforded the product in low yields (Table 3, entry 5). These observations strengthen the hypothesis of involvement of polar groups like –OH and –COOH groups of the catalyst *via* hydrogen bonding and proves their essentiality in the catalysis.

**Fig. 7:**
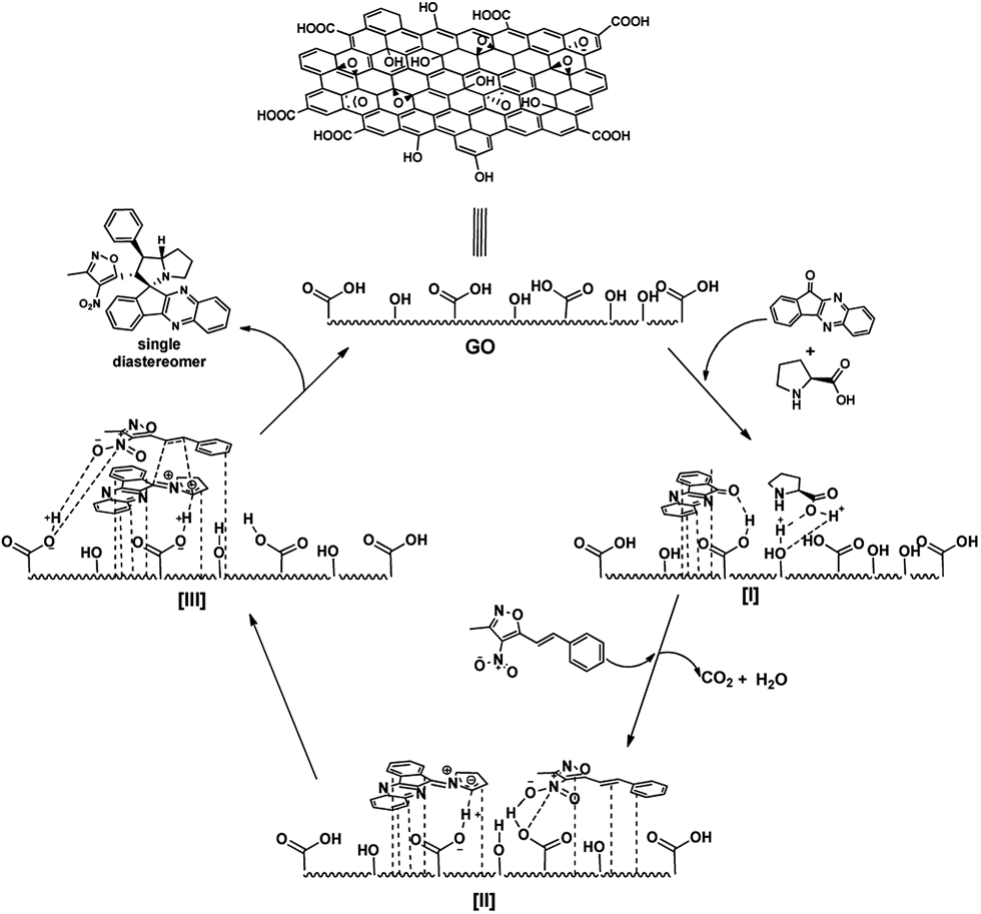
Plausible mechanism GO catalysed spiro pyrrolizidines synthesis.

From all these observation, we assumed the following plausible mechanistic path for the reaction (Fig. 7). Initially the reactants are localized onto the surface of the catalyst due π-stacking and hydrogen bonding capability of the catalyst I & II. It is well established that the GO possess acidic nature, it facilitates the imine formation and decarboxylation to generated reactive azomethine ylide II. The dipolarophile approaches the reactive ylide exclusively from one side due to complete shielding of one face by GO sheet, yielding single diastereomer as the product III. The regioselectivity can be reasonable assumed as, possible additional interaction between heteroatoms in the isoxazole motif with the azomethine ylide dipole generated (zwitter ion) during the reaction drives the orientation of incoming dipolarophile (3-methyl-4-nitro-5-alkenylisoxazoles) onto the dipole III.

## Conclusions

In conclusion, we developed a robust, rapid, aqueous, green, efficient catalytic method for the synthesis of polyheterocyclic spiro-indenoquinoxaline pyrrolizidines and spiro-oxindoles pyrrolizidine using 3-methyl-4-nitro-5-alkenylisoxazoles 1a–l as dipolarophile. Good to excellent yields were obtained with excellent regio and diastereoselectivities, without using column chromatography. Heterogenous and highly economical GO was used as recyclable catalyst with ultra-low catalytic loading (0.50 wt%). The efficiency of the methodology was established with a gram scale reaction (10 mmol) using 1b/2b and 1b/5a in two separate experiments and it was observed to be equally efficient in both cases. The recyclability experiment was done simultaneously and it was found that GO catalyst could be reused successfully with minimal loss of activity. The recycled catalyst was subjected to various analytical techniques like IR, Raman and Powder XRD to investigate the structural changes in the catalyst and found that no considerable changes could be seen from the results. To understand the importance of surface polar groups of GO, we performed control experiments with various carbon catalysts using 1b, 2b and 3 as substrates and found that polar functionalities including OH and COOH and the surface area are playing the vital in the catalytic activity. From these observations we proposed plausible mechanism to explain the diastereoselectivity and regioselectivity. To best of our knowledge this the first report of aqueous 1,3 dipolar cycloaddition reactions involving azomethine ylides using GO as heterogeneous catalysis. We believe that this study will help researcher to develop possible asymmetric catalyst by functionalising GO.

## Conflicts of interest

There are no conflicts to declare.

## Supplementary Material

RA-008-C8RA06714G-s001

RA-008-C8RA06714G-s002
