# Supplementary material for: Heterogeneous graphene oxide as recyclable catalyst for azomethine ylide mediated 1,3 dipolar cycloaddition reaction in aqueous medium
Source: RSC Adv. 2018 Oct 17;8(62):35587–93. doi: 10.1039/c8ra06714g (PMC9088037; doi:10.1039/c8ra06714g)
Supplement: RA-008-C8RA06714G-s001 [file RA-008-C8RA06714G-s001.pdf]

Electronic Supplementary Material (ESI) for RSC Advances.
This journal is © The Royal Society of Chemistry 2018

Supporting Information

Heterogeneous Graphene Oxide as Recyclable Catalyst for Azomethine Ylide Mediated 1, 3 dipolar Cycloaddition Reaction in Aqueous Medium

Marri Sameer Reddy, Nandigama Satish Kumar, L. Raju Chowhan*

Contents

General Experimental procedure S2

General procedure S2

Analytical data of Spectral data of Spiro-Indenoquinoxaline Pyrrolizidines (4a-u) S3- S14

Analytical data of Spirooxindoles Pyrrolizidines (6a-n) S14- S23

^{1}H NMR and ^{13}C of all compounds S24-S60

1. General Experimental Details

All commercially available chemicals were used without further purification. ^{1}H NMR spectra were obtained on Bruker 500 MHz FT-NMR and 400 MHz FT-NMR spectrometers. ^{13}C NMR spectra were recorded at 125 MHz and 100 MHz. Chemical shifts are reported in relative to the solvent signal. Multiplicity is indicated as follows: s (singlet); bs (broad singlet); d (doublet); t (triplet); q (quartet); m (multiplet); dd (doublet of doublets), etc. TOF and quadrupole mass analyzer types are used for the HRMS measurements IR spectra were recorded on FT/IR-5700 instrument.3,5-dimethyl-4-nitroisoxazoles were synthesized as described in the literature[1].

1.1 General procedure

A mixture of indenoquinoxalinone **2a-c** (0.5 mmol) or isatins **5a-e** and L-proline **3** (0.6 mmol) and 3-methyl-4-nitro-5-alkenyl-isoxazoles **1a-l** (0.6 mmol) to stirring suspension of graphene oxide (0.50 wt.% in water) in water:ethanol (80:20) solvent system and stirred under air atmosphere for the 30 min. reaction can be visualized by the change of color change in the reaction pot. Once the starting material was found to be consumed (by TLC), EtOH was removed under reduced pressure and the aqueous layer was diluted with ethyl acetate, leading to formation of two distinct layer with catalyst GO at inter phase of the water/ethyl acetate layers. Then this mixture is filtered through filter paper and the organic layer is separated followed by extracting water layers with EtOAc (5 x 3 mL). The organic layer was washed with brine, dried over anhydrous $NaSO_4$ and passed through pad of celite to trap any particulate impurities of catalyst. Combined organic layer was concentrating under vacuum to afford crude solid which was further purified by washing with 2 x 5 mL cold methanol to afford pure products **4a–u** and **6a-l** as colorless to pale yellow solids.

Recyclability experiment was done on 10 mmol scale; at the end of the experiment GO was recovered by dissolving the solid product in ethyl acetate followed by centrifugation at 15000 rpm for 1 hours and washed by repeating the process for five times to remove all the traces of organic compounds on GO catalyst.

1.2 Spectral data of Spiro-Indenoquinoxaline Pyrrolizidines

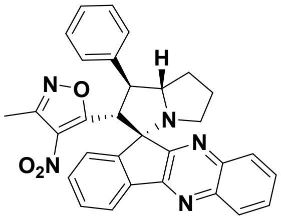

4a

3-methyl-4-nitro-5-((1'S,2'R,7a'S,11S)-1'-phenyl-1',2',5',6',7',7a'-hexahydrospiro[indeno[1,2-b]quinoxaline-11,3'-pyrrolizin]-2'-yl)isoxazole (4a): yellow solid. M.P. 202- 203 °C. IR (neat): 3023, 2939, 1595, 1513, 1415, 1358, 1337, 1143, 1102, 1000, 826, 755, 703 cm^{-1}. ^{1}H NMR (400 MHz, CDCl3) δ 8.35–8.30 (m, 1H), 8.15–8.09 (m, 2H), 7.79 – 7.73 (m, 2H), 7.67–7.62 (m, 2H), 7.52 – 7.42 (m, 3H), 7.37 (dd, J = 10.4, 4.7 Hz, 2H), 7.30 – 7.24 (m, 1H), 5.59 (d, J = 11.6 Hz, 1H), 4.71 (dt, J = 9.8, 6.1 Hz, 1H), 4.18 (dd, J = 11.5, 9.9 Hz, 1H), 2.99–2.88 (m, 1H), 2.58 (ddd, J = 13.7, 7.9, 4.3 Hz, 1H), 2.19 (s, 3H), 2.18–2.07 (m, 2H), 2.06–1.94 (m, 1H), 1.92–1.82 (m, 1H). ^{13}C NMR (100 MHz, $CDCl_3$) δ 171.40, 164.40, 155.72, 152.42, 143.54, 142.98, 142.27, 138.76, 138.15, 130.73, 130.16, 130.09, 129.79, 129.27, 129.22, 129.13, 128.21, 127.69, 127.08, 122.65, 75.91, 72.44, 57.32, 53.62, 47.80, 29.40, 27.14, 11.45. HRMS (ESI+): *m/z* calculated for $[C_{31}H_{25}N_5O_3+H^+]$: 516.2030; found: 516.2033.

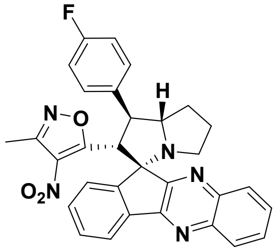

4b

5-((1'S,2'R,7a'S,11S)-1'-(4-fluorophenyl)-1',2',5',6',7',7a'-hexahydrospiro[indeno[1,2-b]quinoxaline-11,3'-pyrrolizin]-2'-yl)-3-methyl-4-nitroisoxazole (4b): Pale yellow solid. M.P. 204-205°C. IR (neat): 3439, 3019, 2963, 2801, 1603, 1512, 1407, 1373, 1334, 1226, 827, 756. ^{1}H NMR (400 MHz, $CDCl_3$) δ 8.33–8.28 (m, 1H), 8.16–8.09 (m, 2H), 7.79–7.74 (m, 2H), 7.64–7.58 (m, 2H), 7.52–7.43 (m, 3H), 7.10–7.02 (m, 2H), 5.51 (d, J = 11.5 Hz, 1H), 4.67 (dt, J = 9.8, 6.2 Hz, 1H), 4.16 (dd, J = 11.4, 9.9 Hz, 1H), 2.93 (dd, J = 15.8, 8.3 Hz, 1H), 2.60–2.53 (m, 1H), 2.21 (s, 3H), 2.18–2.07 (m, 2H), 2.05–1.93 (m, 1H), 1.89–1.79 (m, 1H). ^{13}C NMR (125 MHz, $CDCl_3$) δ 171.30, 164.35, 163.35, 161.39, 155.80, 152.43, 143.46, 143.00, 142.20, 138.17,

134.49, 130.76, 130.22, 130.02, 129.84, 129.75, 129.68, 129.32, 129.27, 125.98, 122.70, 116.15, 115.98, 75.74, 72.37, 57.56, 52.84, 47.66, 29.26, 27.10, 11.44. ^{19}F NMR: (471 MHz, CDCl$_3$) δ -115.33. HRMS (ESI+): *m/z* calculated for [$C_{31}H_{24}FN_5O_4$+H^+]: 534.1936; found: 534.1942.

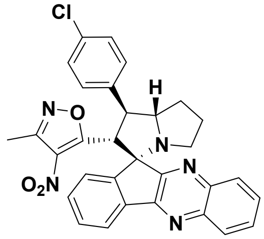

4c

5-((1'S,2'R,7a'S,11S)-1'-(4-chlorophenyl)-1',2',5',6',7',7a'-hexahydrospiro[indeno[1,2-b]quinoxaline-11,3'-pyrrolizin]-2'-yl)-3-methyl-4-nitroisoxazole (4c): Pale yellow solid. M. P. 200-201 °C. IR (neat): 3030, 2965, 1597, 1517, 1416, 1363, 1091, 1004, 828, 761, 739 cm^{-1}. ^{1}H NMR (400 MHz, CDCl$_3$) δ 8.26–8.20 (m, 1H), 8.08–8.01 (m, 2H), 7.72–7.66 (m, 2H), 7.54–7.50 (m, 2H), 7.44 – 7.34 (m, 3H), 7.30 – 7.25 (m, 2H), 5.44 (d, *J* = 11.5 Hz, 1H), 4.60 (dt, *J* = 9.8, 6.1 Hz, 1H), 4.08 (dd, *J* = 11.4, 9.9 Hz, 1H), 2.86 (dd, *J* = 16.0, 8.2 Hz, 1H), 2.50 (ddd, *J* = 9.0, 6.5, 3.6 Hz, 1H), 2.14 (s, 3H), 2.11–2.00 (m, 2H), 1.98 – 1.86 (m, 1H), 1.81 – 1.71 (m, 1H). ^{13}C NMR (101 MHz, CDCl$_3$) δ 171.12, 164.27, 155.77, 152.37, 143.34, 142.96, 142.14, 138.13, 137.30, 133.52, 130.74, 130.21, 129.97, 129.83, 129.52, 129.31, 129.28, 129.26, 126.92, 122.67, 75.72, 72.27, 57.39, 52.94, 47.64, 29.21, 27.06, 11.40. HRMS (ESI+): *m/z* calculated for [$C_{32}H_{24}ClN_5O_3$+H^+]: 550.1646; found: 550.1647.

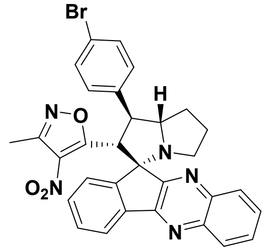

4d

5-((1'S,2'R,7a'S,11S)-1'-(4-bromophenyl)-1',2',5',6',7',7a'-hexahydrospiro[indeno[1,2-b]quinoxaline-11,3'-pyrrolizin]-2'-yl)-3-methyl-4-nitroisoxazole (4d): pale yellow solid. M. P. 205-206 °C. IR (neat): 3024, 1597, 1518, 1416, 1372, 1112, 1008, 831, 762 cm^{-1}. ^{1}H NMR (500 MHz, CDCl$_3$) δ 8.25–8.20 (m, 1H), 8.09–8.01 (m, 2H), 7.69 (dd, *J* = 6.2, 3.5 Hz, 2H), 7.49–7.40 (m, 5H), 7.38–7.33 (m, 2H), 5.44 (d, *J* = 11.5 Hz, 1H), 4.59 (dt, *J* = 9.7, 6.1 Hz, 1H), 4.09–4.04 (m, 1H), 2.86 (dd, *J* = 15.9, 8.2 Hz, 1H), 2.50 (ddd, *J* = 12.3, 8.2, 3.9 Hz, 1H), 2.14 (s,

3H), 2.09 – 1.99 (m, 2H), 1.98 – 1.86 (m, 1H), 1.81 – 1.71 (m, 1H). ^{13}C NMR (125 MHz, $CDCl_3$) δ 171.15, 164.28, 155.82, 152.41, 143.36, 143.01, 142.18, 138.18, 137.88, 132.30, 130.77, 130.69, 130.25, 130.01, 129.93, 129.87, 129.33, 129.30, 125.95, 122.71, 121.64, 75.77, 72.28, 57.38, 53.04, 47.68, 29.25, 27.09, 11.45. HRMS (ESI+): *m/z* calculated for $[C_{31}H_{24}BrN_5O_3+H^+]$: 594.1141; found: 594.1135.

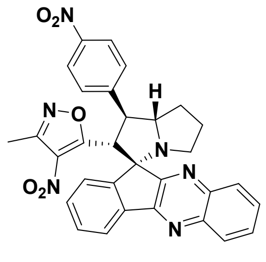

4e

3-methyl-4-nitro-5-((1'S,2'R,7a'S,11S)-1'-(4-nitrophenyl)-1',2',5',6',7',7a'-hexahydrospiro[indeno[1,2-b]quinoxaline-11,3'-pyrrolizin]-2'-yl)isoxazole (4e): pale yellow solid. M. P. 203-204 °C. IR (neat): 3018, 2930, 1597, 1514, 1415, 1343, 1343, 1174, 1145, 1105,831, 767 cm^{-1}. ^{1}H NMR (500 MHz, $CDCl_3$) δ 8.25–8.21 (m, 1H), 8.21–8.17 (m, 2H), 8.10–8.04 (m, 2H), 7.84–7.80 (m, 2H), 7.75–7.69 (m, 2H), 7.43 (td, J = 7.5, 1.0 Hz, 1H), 7.36 (td, J = 7.5, 1.2 Hz, 1H), 7.28 (d, J = 7.7 Hz, 1H), 5.45 (d, J = 11.2 Hz, 1H), 4.70 (dt, J = 9.7, 6.0 Hz, 1H), 4.22 (dd, J = 10.9, 10.0 Hz, 1H), 2.91 (dd, J = 16.0, 8.2 Hz, 1H), 2.55–2.48 (m, 1H), 2.16 (s, 3H), 2.13–2.01 (m, 2H), 1.99–1.89 (m, 1H), 1.83–1.73 (m, 1H) . ^{13}C NMR (125 MHz, $CDCl_3$) δ 171.04, 164.29, 156.01, 152.42, 147.64, 146.81, 143.07, 143.04, 142.01, 138.29, 130.83, 130.41, 129.99, 129.92, 129.43, 129.25, 125.73, 124.44, 122.81, 75.66, 72.35, 57.59, 53.14, 47.55, 28.91, 26.88, 11.43. HRMS (ESI+): *m/z* calculated for $[C_{31}H_{24}N_6O_5+H^+]$: 561.1886; found: 561.1885.

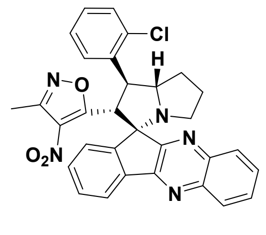

4f

5-((1'S,2'R,7a'S,11S)-1'-(2-chlorophenyl)-1',2',5',6',7',7a'-hexahydrospiro[indeno[1,2-b]quinoxaline-11,3'-pyrrolizin]-2'-yl)-3-methyl-4-nitroisoxazole (4f): pale yellow solid. M. P.

219-220 °C. IR (neat): 3070, 2961, 2827, 1592, 1511, 1415, 1361, 1144, 1034, 1009, 901, 827, 757, 699 cm^{-1}. ^{1}H NMR (400 MHz, CDCl3) δ 8.34–8.29 (m, 1H), 8.16–8.06 (m, 3H), 7.80–7.73 (m, 2H), 7.55–7.44 (m, 3H), 7.37 (m, 2H), 7.19 (m, 1H), 5.64 (d, *J* = 11.5 Hz, 1H), 4.64 (dt, *J* = 9.7, 5.9 Hz, 1H), 3.00 (dd, *J* = 16.4, 7.6 Hz, 1H), 2.61 (ddd, *J* = 9.1, 7.2, 3.5 Hz, 1H), 2.20 (s, 3H), 2.18 – 2.07 (m, 2H), 2.07–1.92 (m, 2H). ^{13}C NMR (100 MHz, $CDCl_3$) δ 171.16, 164.30, 155.66, 152.47, 143.39, 143.05, 142.23, 138.21, 136.48, 134.92, 130.91, 130.25, 130.04, 129.87, 129.33, 129.27, 128.82, 128.58, 127.80, 127.21, 122.70, 76.22, 73.40, 56.66, 48.38, 47.99, 29.15, 27.07, 11.46. HRMS (ESI+): *m/z* calculated for $[C_{31}H_{24}ClN_5O_4+H^+]$: 550.1640; found: 550.1639.

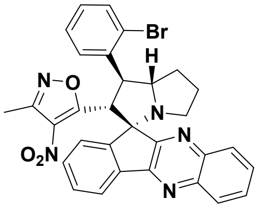

4g

5-((1'S,2'R,7a'S,11S)-1'-(2-bromophenyl)-1',2',5',6',7',7a'-hexahydrospiro[indeno[1,2-b]quinoxaline-11,3'-pyrrolizin]-2'-yl)-3-methyl-4-nitroisoxazole (4g): yellow solid. M.P. 206-207 °C. IR (neat): 3070, 2961, 2832, 1592, 1511, 1471, 1415, 1361, 1226, 1021, 826, 758 cm^{-1}. ^{1}H NMR (400 MHz, $CDCl_3$) δ 8.35–8.29 (m, 1H), 8.16–8.09 (m, 3H), 7.80–7.74 (m, 2H), 7.58 (dd, *J* =8.0, 1.2 Hz, 1H), 7.55–7.44 (m, 3H), 7.43–7.38 (m, 1H), 7.12 (td, *J* = 8.0, 1.6 Hz, 1H), 5.62 (d, *J* = 11.5 Hz, 1H), 5.00 (dd, *J* = 11.5, 9.8 Hz, 1H), 4.63 (dt, *J* = 9.8, 6.0 Hz, 1H), 3.05–2.97 (m, 1H), 2.62 (ddd, *J* = 9.2, 7.3, 3.4 Hz, 1H), 2.21 (s, 3H), 2.18–1.92 (m, 4H). ^{13}C NMR (100 MHz, $CDCl_3$) δ 171.11, 164.24, 155.66, 152.40, 143.27, 142.98, 142.17, 138.16, 133.34, 130.92, 130.26, 129.99, 129.88, 129.27, 128.93, 128.43, 127.19, 125.63, 122.68, 76.27, 73.73, 56.82, 51.04, 48.15, 28.94, 26.97, 11.49. HRMS (ESI+): *m/z* calculated for $[C_{31}H_{24}BrN_5O_3+H^+]$: 594.1141; found: 594.1135.

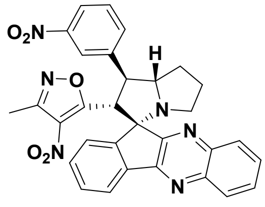

4h

3-methyl-4-nitro-5-((1'S,2'R,7a'S,11S)-1'-(3-nitrophenyl)-1',2',5',6',7',7a'-hexahydrospiro[indeno[1,2-b]quinoxaline-11,3'-pyrrolizin]-2'-yl)isoxazole (4h): pale yellow solid. M. P. 229-230°C.IR (neat): 3440, 3038, 2950, 1594, 1526, 1416, 1376, 1351,1144, 1107, 828, 767, 737 cm^{-1}. ^{1}H NMR (400 MHz, $CDCl_3$) δ 8.86 (t, *J* = 1.9 Hz, 1H), 8.49 (t, *J* = 1.9 Hz, 1H), 8.41 (dt, *J* = 5.6, 3.2 Hz, 3H), 8.16 (m, 3H), 8.00 (d, *J* = 7.7 Hz, 1H), 7.84–7.76 (m, 2H), 7.56 (t, *J* = 7.9 Hz, 1H), 7.50 (dt, *J* = 7.6, 3.8 Hz, 1H), 7.40 (td, *J* = 7.6, 1.1 Hz, 1H), 7.24 (m, 1H), 5.44 (d, *J* = 10.6 Hz, 1H), 4.84 (ddd, *J* = 9.9, 6.2, 5.5 Hz, 1H), 4.30 (t, *J* = 10.2 Hz, 1H), 3.02 (dd, *J* = 15.8, 8.3 Hz, 1H), 2.64–2.56 (m, 1H), 2.27 (s, 3H), 2.20–2.09 (m, 2H), 2.05–1.94 (m, 1H), 1.91–1.81 (m, 1H). ^{13}C NMR (100 MHz, $CDCl_3$) δ 171.73, 164.50, 156.12, 149.06, 143.04, 141.93, 141.85, 138.46, 134.60, 130.74, 130.40, 130.09, 130.01, 129.54, 129.36, 125.58, 123.67, 122.82, 75.69, 72.46, 57.81, 52.80, 47.78, 28.57, 26.53, 11.47. HRMS (ESI+): *m/z* calculated for $[C_{31}H_{24}N_6O_5+H^+]$: 561.1886; found: 561.1884.

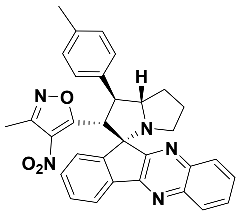

4i

3-methyl-4-nitro-5-((1'S,2'R,7a'S,11S)-1'-(p-tolyl)-1',2',5',6',7',7a'-hexahydrospiro[indeno[1,2-b]quinoxaline-11,3'-pyrrolizin]-2'-yl)isoxazole (4i): yellow solid. M. P. 202-203°C. IR (neat): 3015, 2945, 2863, 1588, 1512, 1463, 1415, 1358, 1338, 1144, 1001, 906, 825, 764 cm^{-1}. ^{1}H NMR (400 MHz, $CDCl_3$) δ 8.36–8.28 (m, 1H), 8.16–8.07 (m, 2H), 7.81–7.70 (m, 2H), 7.59–7.40 (m, 5H), 7.18 (d, *J* = 7.8 Hz, 2H), 5.57 (d, *J* = 11.7 Hz, 1H), 4.68 (dt, *J* = 9.8, 6.2 Hz, 1H), 4.15 (dd, *J* = 11.6, 9.9 Hz, 1H), 2.99–2.88 (m, 1H), 2.62–2.54 (m, 1H), 2.32 (s, 3H), 2.18 (s, 3H), 2.17–2.07 (m, 1H), 2.05–1.94 (m, 1H), 1.91–1.81 (m, 1H). ^{13}C NMR (125 MHz, $CDCl_3$) δ 171.40, 164.40, 155.67, 152.44, 143.64, 143.00, 142.32, 138.15, 137.39, 135.65, 130.72, 130.12, 129.83, 129.77, 129.27, 129.20, 128.06, 127.13, 122.64, 75.93, 72.44, 57.34, 53.36, 47.78, 29.47, 27.21, 21.20, 11.43. HRMS (ESI+): *m/z* calculated for $[C_{32}H_{27}N_5O_3+H^+]$: 530.2192; found: 530.2191.

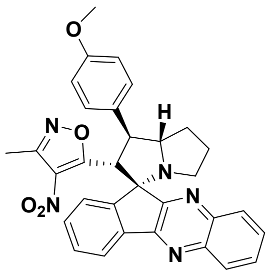

4j

5-((1'S,2'R,7a'S,11S)-1'-(4-methoxyphenyl)-1',2',5',6',7',7a'-hexahydrospiro[indeno[1,2-b]quinoxaline-11,3'-pyrrolizin]-2'-yl)-3-methyl-4-nitroisoxazole (4j): pale yellow solid. M. P. 199-201 °C. IR (neat): 3054, 2967, 2941, 2832, 1587, 1505, 1453, 1412, 1365, 1247, 1169, 1086, 1030, 823, 756 cm^{-1}. ^{1}H NMR (500 MHz, $CDCl_3$) δ 8.34–8.31 (m, 1H), 8.14–8.08 (m, 2H), 7.78–7.74 (m, 2H), 7.57–7.53 (m, 2H), 7.51–7.43 (m, 3H), 6.92–6.88 (m, 2H), 5.54 (d, *J* = 11.7 Hz, 1H), 4.66 (dt, *J* = 9.8, 6.2 Hz, 1H), 4.14 (dd, *J* = 11.6, 9.9 Hz, 1H), 3.79 (s, 3H), 2.93 (dt, *J* = 8.4, 7.0 Hz, 1H), 2.61–2.56 (m, 1H), 2.19 (s, 3H), 2.17–2.08 (m, 2H), 2.04–1.95 (m, 1H), 1.90–1.81 (m, 1H). ^{13}C NMR (125 MHz, $CDCl_3$) δ 171.40, 164.36, 159.17, 155.69, 152.42, 143.60, 142.99, 142.29, 138.12, 130.73, 130.09, 129.78, 129.21, 127.12, 122.65, 114.54, 75.89, 72.37, 57.42, 55.39, 53.01, 47.81, 29.48, 27.20, 11.45. HRMS (ESI+): *m/z* calculated for $[C_{32}H_{27}N_5O_4+H^+]$: 546.2136; found: 546.2140.

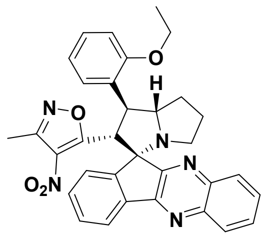

4k

5-((1'S,2'R,7a'S,11S)-1'-(2-ethoxyphenyl)-1',2',5',6',7',7a'-hexahydrospiro[indeno[1,2-b]quinoxaline-11,3'-pyrrolizin]-2'-yl)-3-methyl-4-nitroisoxazole (4k): yellow solid. M. P. 182-183 °C.IR (neat): 3015, 2970, 2895, 2851, 1600, 1523, 1495, 1452, 1252, 1124, 1100, 1040, 825, 756 cm^{-1}. ^{1}H NMR (400 MHz, $CDCl_3$) δ 8.27–8.22 (m, 1H), 8.06–7.97 (m, 2H), 7.71 – 7.64 (m, 2H), 7.63 – 7.54 (m, 2H), 7.46 – 7.37 (m, 2H), 7.12 (td, *J* = 8.2, 1.6 Hz, 1H), 6.88 (td, *J* = 7.5, 0.8 Hz, 1H), 6.78 (d, *J* = 8.1 Hz, 1H), 5.88 (d, *J* = 11.6 Hz, 1H), 4.67– 4.55 (m, 2H), 4.07–3.97 (m, 2H), 2.87 – 2.79 (m, 1H), 2.57–2.49 (m, 1H), 2.10–1.98 (m, 5H), 1.96–1.84 (m, 2H), 1.54 (t, *J* = 7.0 Hz, 3H). ^{13}C NMR (100 MHz, $CDCl_3$) δ 171.25, 164.18, 157.49, 155.41, 152.35, 143.80, 142.95, 142.49, 137.94, 130.71, 130.11, 130.04, 129.75, 129.14, 128.84, 128.42, 127.46,

125.33, 122.54, 120.99, 111.87, 76.54, 71.53, 64.01, 54.76, 48.02, 47.55, 30.26, 27.51, 15.16, 11.48. HRMS (ESI+): *m/z* calculated for [$C_{31}H_{29}N_5O_4+H^+$]: 560.2298; found: 560.2293.

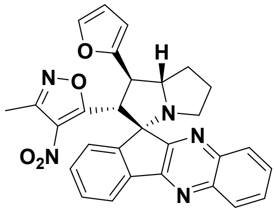

4l

5-((1'R,2'R,7a'S,11S)-1'-(furan-2-yl)-1',2',5',6',7',7a'-hexahydrospiro[indeno[1,2-b]quinoxaline-11,3'-pyrrolizin]-2'-yl)-3-methyl-4-nitroisoxazole (4l): pale yellow solid.R_f = 0.35 (20%EtOAc/Hexane). M.P. 190-191°C.IR (neat): 3050, 1614, 1516, 1413, 1324, 1164, 801, 707, 676 cm^{-1}. ^1H NMR (400 MHz, CDCl3) δ 8.22–8.18 (m, 1H), 8.05–7.99 (m, 2H), 7.69–7.63 (m, 2H), 7.43–7.37 (m, 3H), 7.27 (dd, *J* = 1.8, 0.7 Hz, 1H), 6.25–6.18 (m, 2H), 5.52 (d, *J* = 11.8 Hz, 1H), 4.63–4.57 (m, 1H), 4.24 (dd, *J* = 11.7, 9.8 Hz, 1H), 2.80–2.73 (m, 1H), 2.54–2.45 (m, 1H), 2.24–2.14 (m, 4H), 2.10–1.83 (m, 3H). ^{13}C NMR (125 MHz, $CDCl_3$) δ 170.61, 163.75, 155.66, 152.47, 152.32, 143.41, 142.98, 142.37, 138.02, 130.77, 130.20, 130.12, 129.82, 129.20, 127.15, 122.63, 110.49, 106.54, 75.79, 69.48, 54.58, 47.47, 46.70, 30.05, 27.45, 11.47. HRMS (ESI+): *m/z* calculated for [$C_{29}H_{23}N_5O_4+H^+$]: 506.1823; found: 506.1823.

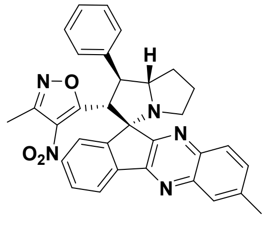

4m

3-methyl-5-((1'S,2'R,7a'S,11S)-7-methyl-1'-phenyl-1',2',5',6',7',7a'-hexahydrospiro[indeno[1,2-b]quinoxaline-11,3'-pyrrolizin]-2'-yl)-4-nitroisoxazole (4m): pale yellow solid. M. P. 189-190°C.IR (neat): 3070,2961, 2827, 1592, 1511, 1511, 1415, 1361, 1144, 1102, 1034, 1009, 827, 757, 699 cm^{-1}. ^1H NMR (400 MHz, $CDCl_3$) δ 8.21 (d, *J* = 8.5 Hz, 1H), 8.11–8.06 (m, 1H), 7.90 (s, 1H), 7.67–7.56 (m, 3H), 7.52–7.40 (m, 3H), 7.40–7.32 (m, 2H), 7.30 –7.24 (m, 1H), 5.59 (d, *J* = 11.7 Hz, 1H), 4.69 (dt, *J* = 9.7, 6.2 Hz, 1H), 4.18 (dd, *J* = 11.5, 9.9 Hz, 1H), 2.93 (dd, *J* = 16.0, 8.2 Hz, 1H), 2.66–2.55 (m, 4H), 2.18 (s, 3H), 2.16–2.07 (m, 2H), 2.05–1.95 (m, 1H), 1.93–1.82 (m, 1H). ^{13}C NMR (125 MHz, $CDCl_3$) δ 171.25, 163.33,

155.67, 152.26, 143.52, 143.02, 140.74, 140.26, 138.77, 138.27, 131.41, 130.55, 130.09, 129.58, 129.11, 128.36, 128.19, 127.66, 127.09, 122.54, 76.00, 72.49, 57.29, 53.66, 47.75, 29.58, 27.26, 21.91, 11.43. HRMS (ESI+): *m/z* calculated for [$C_{32}H_{27}ClN_5O_4$+H^+]: 530.2187; found: 530.2186.

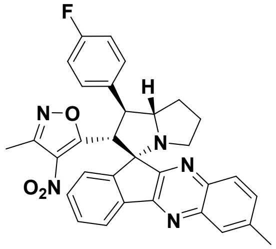

4n

5-((1'S,2'R,7a'S,11S)-1'-(4-fluorophenyl)-7-methyl-1',2',5',6',7',7a'-hexahydrospiro[indeno[1,2-b]quinoxaline-11,3'-pyrrolizin]-2'-yl)-3-methyl-4-nitroisoxazole (4n): pale yellow solid. M.P. 218- 219 °C.IR (neat): 3042, 2970, 2843, 1593, 1507, 1376, 1224, 1148, 1108, 1009, 829, 767 cm^{-1}. ^{1}H NMR (400 MHz, $CDCl_3$) δ 8.12 (d, *J* = 8.5 Hz, 1H), 8.03–7.99 (m, 1H), 7.83 (s, 1H), 7.56–7.50 (m, 3H), 7.44–7.34 (m, 3H), 7.02–6.95 (m, 2H), 5.44 (d, *J* = 11.6 Hz, 1H), 4.58 (dt, *J* = 9.8, 6.2 Hz, 1H), 4.09 (dd, *J* = 11.5, 9.9 Hz, 1H), 2.85 (dd, *J* = 16.0, 8.1 Hz, 1H), 2.58–2.48 (m, 4H), 2.13 (s, 3H), 2.09–2.01 (m, 2H), 1.99–1.89 (m, 1H), 1.82–1.73 (m, 1H). ^{13}C NMR (100 MHz, $CDCl_3$) δ 171.16, 163.27, 155.77, 152.26, 143.39, 143.05, 140.68, 140.34, 138.31, 134.52, 131.47, 130.58, 130.16, 129.74, 129.67, 129.53, 128.41, 127.00, 122.59, 116.16, 115.95, 75.89, 72.45, 57.49, 52.90, 47.72, 29.46, 27.20, 21.93, 11.46. ^{19}F NMR: (471 MHz, $CDCl_3$) δ -114.94 ppm. HRMS (ESI+): *m/z* calculated for [$C_{32}H_{26}FN_5O_4$+H^+]: 548.2098; found: 548.2092.

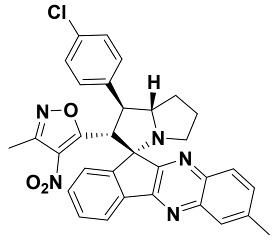

4o

5-((1'S,2'R,7a'S,11S)-1'-(4-bromophenyl)-7-methyl-1',2',5',6',7',7a'-hexahydrospiro[indeno[1,2-b]quinoxaline-11,3'-pyrrolizin]-2'-yl)-3-methyl-4-nitroisoxazole (4o): pale yellow solid. M.P. 210-211°C. IR (neat): 3058, 2969, 1596, 1518, 1416, 1378, 1335, 1091, 1004, 829, 738 cm^{-1}. ^{1}H NMR (500 MHz, $CDCl_3$) δ 8.11 (d, *J* = 8.5 Hz, 1H), 8.01 (d, *J* =

7.2 Hz, 1H), 7.83 (s, 1H), 7.54–7.48 (m, 3H), 7.42–7.33 (m, 3H), 7.27 (d, J = 8.4 Hz, 2H), 5.44 (d, J = 11.6 Hz, 1H), 4.61–4.55 (m, 1H), 4.07 (dd, J = 14.2, 7.2 Hz, 1H), 2.84 (dd, J = 15.9, 8.1 Hz, 1H), 2.58–2.48 (m, 4H), 2.13 (s, 3H), 2.09–2.01 (m, 2H), 1.98–1.88 (m, 1H), 1.78 (d, J = 6.2 Hz, 1H). ^{13}C NMR (100 MHz, $CDCl_3$) δ 171.01, 163.18, 155.78, 152.21, 143.27, 143.03, 140.65, 140.36, 138.28, 137.31, 133.52, 131.48, 130.58, 130.18, 129.54, 129.33, 128.39, 125.98, 75.93, 72.39, 57.33, 53.03, 47.78, 29.45, 27.18, 21.94, 11.48. HRMS (ESI+): *m/z* calculated for $[C_{32}H_{26}ClN_5O_3+H^+]$: 564.1802; found: 564.1804.

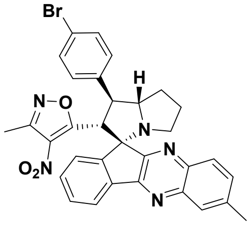

4p

5-((1'S,2'R,7a'S,11S)-1'-(4-bromophenyl)-7-methyl-1',2',5',6',7',7a'-hexahydrospiro[indeno[1,2-b]quinoxaline-11,3'-pyrrolizin]-2'-yl)-3-methyl-4-nitroisoxazole (4p): pale yellow solid. M. P. 210-211 °C. IR (neat):3045, 2960, 1596, 1517, 1416, 1375, 1251, 1179, 1001, 829, 735 cm^{-1}. ^1H NMR (400 MHz, $CDCl_3$) δ 8.11 (d, J = 8.5 Hz, 1H), 8.03–7.99 (m, 1H), 7.83 (s, 1H), 7.52 (dd, J = 8.5, 1.6 Hz, 1H), 7.46 –7.34 (m, 7H), 5.44 (d, J = 11.6 Hz, 1H), 4.57 (tt, J = 10.4, 5.0 Hz, 1H), 4.06 (dd, J = 11.5, 9.9 Hz, 1H), 2.85 (dd, J = 16.0, 8.2 Hz, 1H), 2.57–2.48 (m, 4H), 2.13 (s, 3H), 2.09–1.98 (m, 2H), 1.98–1.87 (m, 1H), 1.82–1.71 (m, 1H). ^{13}C NMR (100 MHz, $CDCl_3$) δ 171.01, 163.19, 155.79, 152.23, 143.30, 143.05, 140.66, 140.38, 138.31, 137.88, 132.29, 131.49, 130.59, 130.19, 129.92, 129.52, 128.41, 125.97, 122.60, 121.62, 75.92, 72.36, 57.32, 53.10, 47.73, 29.44, 27.18, 21.94, 11.46. HRMS (ESI+): *m/z* calculated for $[C_{32}H_{24}BrN_5O_3+H^+]$: 608.1292; found: 608.1292.

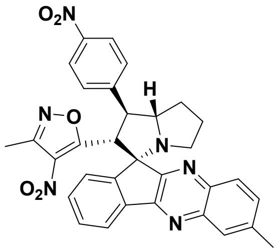

4q

3-methyl-5-((1'S,2'R,7a'S,11S)-7-methyl-1'-(4-nitrophenyl)-1',2',5',6',7',7a'-hexahydrospiro[indeno[1,2-b]quinoxaline-11,3'-pyrrolizin]-2'-yl)-4-nitroisoxazole (4q): yellow solid. M. P. 213-214 °C. IR (neat): 3059, 2995, 1599, 1520, 1415, 1345, 1004, 830, 735 cm^{-1}. ^1H NMR (400 MHz, $CDCl_3$) δ 8.19–8.14 (m, 2H), 8.10 (d, J = 8.5 Hz, 1H), 8.02 (d, J =

7.5 Hz, 1H), 7.84 (s, 1H), 7.81 (t, J = 7.0 Hz, 2H), 7.53 (dd, J = 8.5, 1.7 Hz, 1H), 7.41 (td, J = 7.4, 1.1 Hz, 1H), 7.34 (td, J = 7.5, 1.1 Hz, 1H), 7.29 (d, J = 7.6 Hz, 1H), 5.45 (d, J = 11.3 Hz, 1H), 4.67 (dt, J = 9.7, 6.1 Hz, 1H), 4.24–4.17 (m, 1H), 2.89 (dd, J = 15.9, 8.2 Hz, 1H), 2.57–2.48 (m, 4H), 2.14 (s, J = 2.9 Hz, 3H), 2.10–2.00 (m, 2H), 1.98–1.89 (m, 1H), 1.83–1.72 (m, 1H). ^{13}C NMR (100 MHz, $CDCl_3$) δ 170.90, 163.21, 155.95, 152.22, 147.58, 146.81, 143.04, 142.98, 140.49, 140.45, 138.39, 131.58, 130.62, 130.32, 129.39, 129.22, 128.48, 125.72, 124.39, 122.66, 75.76, 72.39, 57.49, 53.16, 47.59, 29.03, 26.93, 21.92, 11.41. HRMS (ESI+): *m/z* calculated for [$C_{31}H_{26}N_6O_5+H^+$]: 575.2043; found: 575.2046.

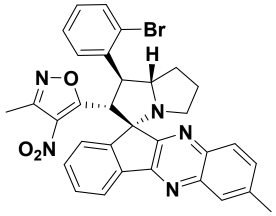

4r

5-((1'S,2'R,7a'S,11S)-1'-(2-bromophenyl)-7-methyl-1',2',5',6',7',7a'-hexahydrospiro[indeno[1,2-b]quinoxaline-11,3'-pyrrolizin]-2'-yl)-3-methyl-4-nitroisoxazole (4r): pale yellow solid. M.P. 206-207 °C. IR (neat): 3048, 2959, 1596, 1517, 1414, 1378, 1334, 1106, 1022, 87, 755 cm^{-1}. ^{1}H NMR (500 MHz, $CDCl_3$) δ 8.13 (d, J = 8.4 Hz, 1H), 8.01 (dd, J = 10.4, 7.2 Hz, 2H), 7.84 (s, 1H), 7.55–7.44 (m, 3H), 7.41 (td, J = 13.0, 7.4 Hz, 2H), 7.33 (dd, J = 14.6, 7.1 Hz, 1H), 7.04 (t, J = 7.6 Hz, 1H), 5.55 (d, J = 11.5 Hz, 1H), 4.92 (t, J = 10.6 Hz, 1H), 4.58–4.50 (m, 1H), 2.91 (dd, J = 16.1, 8.2 Hz, 1H), 2.60–2.50 (m, 4H), 2.15–1.85 (m, 7H). ^{13}C NMR (125 MHz, $CDCl_3$) δ 170.96, 163.14, 155.78, 152.18, 143.22, 143.00, 140.64, 140.39, 138.24, 137.26, 133.51, 131.50, 130.59, 130.19, 129.54, 129.50, 129.32, 128.36, 125.99, 122.59, 75.98, 72.40, 57.28, 53.05, 47.85, 29.49, 27.19, 21.96, 11.51. HRMS (ESI+): *m/z* calculated for [$C_{32}H_{26}BrN_5O_3+H^+$]: 608.1297; found: 608.1297.

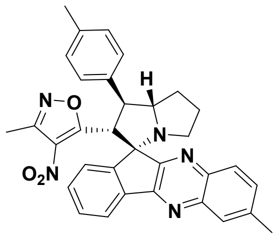

4s

3-methyl-5-((1'S,2'R,7a'S,11S)-7-methyl-1'-(p-tolyl)-1',2',5',6',7',7a'-hexahydrospiro[indeno[1,2-b]quinoxaline-11,3'-pyrrolizin]-2'-yl)-4-nitroisoxazole (4s): pale yellow solid. M. P. 197-198 °C. IR (neat): 3059, 2948, 1590, 1506, 1448, 1414, 1376, 1360, 1333, 1143, 1008, 892, 830, 754, 668 cm^{-1}. ^{1}H NMR (400 MHz, $CDCl_3$) δ 8.13 (d, *J* = 8.5 Hz, 1H), 8.02–7.98 (m, 1H), 7.82 (s, 1H), 7.51 (dd, *J* = 8.5, 1.8 Hz, 1H), 7.46–7.34 (m, 5H), 7.09 (d, *J* = 7.9 Hz, 2H), 5.50 (d, *J* = 11.8 Hz, 1H), 4.58 (dt, *J* = 9.7, 6.2 Hz, 1H), 4.06 (dt, *J* = 7.0, 6.5 Hz, 1H), 2.84 (dd, *J* = 16.1, 8.2 Hz, 1H), 2.57–2.49 (m, 4H), 2.24 (s, 3H), 2.09 (s, 3H), 2.07–1.99 (m, 2H), 1.98–1.86 (m, 1H), 1.84–1.73 (m, 1H). ^{13}C NMR (100 MHz, $CDCl_3$) δ 171.21, 163.27, 155.62, 152.23, 143.52, 143.01, 140.77, 140.24, 138.23, 137.36, 135.61, 131.39, 130.53, 130.06, 129.80, 129.59, 128.33, 128.03, 127.15, 122.51, 76.09, 72.51, 57.23, 53.40, 47.86, 29.67, 27.30, 21.92, 21.20, 11.45. HRMS (ESI+): *m/z* calculated for [$C_{33}H_{29}N_5O_3$+H+]: 544.2343; found: 544.2408.

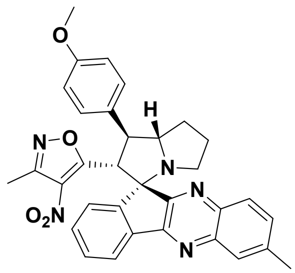

4t

5-((1'S,2'R,7a'S,11S)-1'-(4-methoxyphenyl)-7-methyl-1',2',5',6',7',7a'-hexahydrospiro[indeno[1,2-b]quinoxaline-11,3'-pyrrolizin]-2'-yl)-3-methyl-4-nitroisoxazole (4t): pale yellow solid. M.P. 206-207 °C. IR (neat): 3062, 2961, 2920, 2843, 1587, 1507, 1469, 1412, 1360, 1329, 1241, 1179, 1143, 1030, 828, 740 cm^{-1}. ^{1}H NMR (400 MHz, $CDCl_3$) δ 8.21 (d, *J* = 8.5 Hz, 1H), 8.10–8.06 (m, 1H), 7.89 (s, 1H), 7.61–7.42 (m, 6H), 6.91–6.86 (m, 2H), 5.54 (d, *J* = 11.8 Hz, 1H), 4.64 (dt, *J* = 9.7, 6.2 Hz, 1H), 4.13 (dd, *J* = 11.6, 10.0 Hz, 1H), 3.78 (s, 3H), 2.91 (dd, *J* = 16.1, 8.2 Hz, 1H), 2.65–2.55 (m, 4H), 2.18 (s, *J* = 2.6 Hz, 3H), 2.16–2.07 (m, 2H), 2.05–1.95 (m, 1H), 1.90–1.80 (m, 1H). ^{13}C NMR (125 MHz, $CDCl_3$) δ 171.26, 159.16, 155.66,152.25, 143.04, 140.74, 140.27, 138.25, 131.41, 130.55, 130.08, 129.60, 129.16, 128.36, 127.15, 122.54, 114.54, 76.03, 72.45, 57.38, 55.40, 53.06, 47.84, 29.68, 27.31, 21.93, 11.47. HRMS (ESI+): *m/z* calculated for [$C_{33}H_{29}N_5O_4+H^+$]: 560.2291; found: 560.2292.

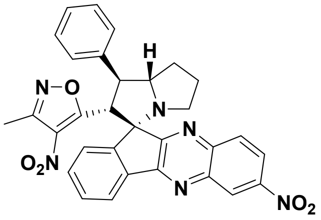

4u

3-methyl-4-nitro-5-((1'S,2'R,7a'S,11S)-7-nitro-1'-phenyl-1',2',5',6',7',7a'-hexahydrospiro[indeno[1,2-b]quinoxaline-11,3'-pyrrolizin]-2'-yl)isoxazole (4u): yellow solid. M. P. 219-220 °C. IR (neat):3080, 2946, 2853, 1587, 1510, 1448, 1417, 1340, 1148, 1071, 823, 735, 694 cm^{-1}. ^{1}H NMR (500 MHz, CDCl3) δ 9.22 (d, *J* = 1.7 Hz, 1H), 8.57–8.52 (m, 1H), 8.25 (d, *J* = 9.1 Hz, 1H), 8.14 (d, *J* = 6.2 Hz, 1H), 7.63 (d, *J* = 7.4 Hz, 2H), 7.56–7.45 (m, 3H), 7.41 (t, *J* = 7.4 Hz, 2H), 7.30 (t, *J* = 7.2 Hz, 1H), 5.51 (d, *J* = 11.6 Hz, 1H), 4.72–4.66 (m, 1H), 4.17 (t, *J* = 10.7 Hz, 1H), 2.97 (dd, *J* = 15.7, 7.9 Hz, 1H), 2.59–2.52 (m, 1H), 2.21 (s, 3H), 2.18–2.09 (m, 2H), 2.04–1.94 (m, 1H), 1.92–1.82 (m, 1H). ^{13}C NMR (100 MHz, $CDCl_3$) δ 171.42, 167.43, 155.84, 155.49, 147.30, 145.98, 144.33, 141.02, 138.28, 137.15, 132.20, 130.64, 130.56, 129.29, 128.16, 127.90, 127.14, 125.27, 123.52, 123.35, 77.36, 75.39, 72.26, 57.58, 53.40, 47.81, 29.02, 26.99, 11.44. HRMS (ESI+): *m/z* calculated for [$C_{31}H_{24}N_6O_5$+H^+]: 561.1881; found: 561.1881.

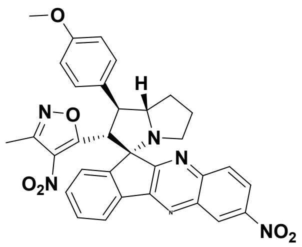

4v

5-((1'S,2'R,7a'S,11S)-1'-(4-methoxyphenyl)-7-nitro-1',2',5',6',7',7a'-hexahydrospiro[indeno[1,2-b]quinoxaline-11,3'-pyrrolizin]-2'-yl)-3-methyl-4-nitroisoxazole (4v): yellow solid. M.P. 194-195 °C.IR (neat):3059, 2964, 1592, 1515, 1416, 137,2 1342, 1248, 1179, 1074, 1037, 906, 829, 736 cm^{-1}. ^{1}H NMR (400 MHz, $CDCl_3$) δ 9.14 (d, *J* = 2.5 Hz, 1H), 8.46 (dd, *J* = 9.1, 2.5 Hz, 1H), 8.17 (d, *J* = 9.1 Hz, 1H), 8.08–8.03 (m, 1H), 7.49–7.38 (m, 5H), 6.88–6.82 (m, 2H), 5.38 (d, *J* = 11.6 Hz, 1H), 4.62–4.53 (m, 1H), 4.06 (dd, *J* = 11.5, 10.1 Hz, 1H), 3.73 (s, 3H), 2.88 (dd, *J* = 15.9, 8.0 Hz, 1H), 2.53–2.44 (m, 1H), 2.13 (s, 3H), 2.10–2.01 (m, 2H), 1.92 (m, 1H), 1.83–1.71 (m, 1H). ^{13}C NMR (100 MHz, $CDCl_3$) δ 171.45, 167.42, 159.31, 155.80, 155.48, 147.29, 145.98, 144.37, 141.03, 137.11, 132.18, 130.63, 130.53, 130.06,

129.13, 127.17, 125.27, 123.50, 123.33, 114.68, 75.37, 72.18, 57.68, 55.43, 52.77, 47.84, 29.07, 27.02, 11.44. HRMS (ESI+): *m/z* calculated for [$C_{32}H_{26}N_6O_6$+H^+]: 591.1987; found: 591.1985.

1.3 Spectral data of Spirooxindoles Pyrrolizidines

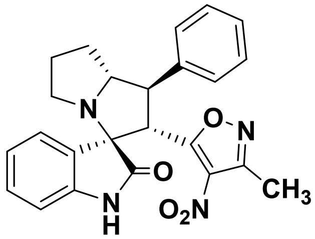

6a

(1'R,2'S,3R,7a'R)-2'-(3-methyl-4-nitroisoxazol-5-yl)-1'-phenyl-1',2',5',6',7',7a'-hexahydrospiro[indoline-3,3'-pyrrolizin]-2-one: White solid. M.P. 127-129 °C. IR (KBr) ν_{max} (cm^{-1}) 3150, 3087, 3032, 2925, 1723, 1598, 1514, 1472, 1417, 1360, 1218, 1145, 1014, 906, 827, 752, 624, 538; ^{1}H NMR (300 MHz, $CDCl_3$+DMSO- d_6) δ 10.12 (s, 1H), 7.54 – 7.48 (m, 3H), 7.36 – 7.27 (m, 2H), 7.24 (t, *J* = 7.3 Hz, 1H), 7.13 (td, *J* = 7.7, 1.4 Hz, 1H), 6.87 – 6.77 (m, 3H), 5.06 (d, *J* = 11.3 Hz, 1H), 4.42 – 4.32 (m, 1H), 3.93 (t, *J* = 10.7 Hz, 1H), 3.06 – 2.95 (m, 2H), 2.71 – 2.62 (m, 1H), 2.29 (s, 3H), 2.10 – 1.97 (m, 3H), 1.94 – 1.71 (m, 3H). ^{13}C NMR (75 MHz, $CDCl_3$+DMSO- d_6) δ 179.68, 171.60, 155.11, 142.36, 138.03, 129.34, 128.44, 127.58, 126.96, 125.50, 124.49, 120.62, 110.07, 77.59, 77.16, 76.73, 73.79, 70.83, 55.63, 52.57, 48.31, 29.15, 28.06, 25.40, 10.88. HRMS (ESI+): m/z calculated for [$C_{24}H_{22}N_4O_4$ +H+]: 431.1714; found: 431.1733.

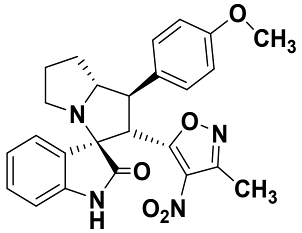

6b

(1'R,2'S,3R,7a'R)-1'-(4-methoxyphenyl)-2'-(3-methyl-4-nitroisoxazol-5-yl)-1',2',5',6',7',7a'-hexahydrospiro[indoline-3,3'-pyrrolizin]-2-one: White solid. M. P. 250-252 °C. IR (KBr) ν_{max} (cm^{-1}) 3147, 3076, 3017, 2936, 1705, 1585, 1508, 1479, 1434, 1401, 1279, 1133, 1001, 901, 816, 744, 668, 536; ^{1}H NMR (300 MHz, $CDCl_3$+DMSO- d_6) δ 10.14 (s, 1H), 7.42 (d, *J* = 8.7 Hz, 2H), 7.13 (td, *J* = 7.6, 1.3 Hz, 1H), 6.84 (m, 5H), 5.00 (d, *J* = 11.4 Hz, 1H), 4.37 – 4.27 (m, 1H), 3.88

(t, J = 10.7 Hz, 1H), 3.77 (s, 3H), 2.98 – 2.94 (m, 1H)2.29 (s, 3H), 2.08 – 1.96 (m, 2H), 1.95 – 1.70 (m, 2H). ^{13}C NMR (75 MHz, $CDCl_3$+DMSO- d_6) δ 178.60, 170.73, 157.56, 154.18, 141.64, 129.06, 128.91, 128.50, 127.62, 124.74, 123.68, 119.71, 113.08, 109.08, 72.78, 69.84, 54.67, 53.91, 50.88, 47.40, 27.18, 24.59, 10.00. HRMS (ESI+): m/z calculated for [$C_{25}H_{24}N_4O_5$ +H+]: 461.1819; found: 461.1835.

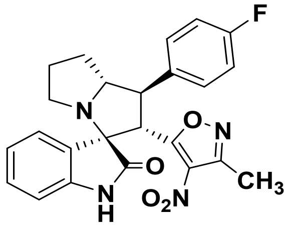

6c

(1'R,2'S,3R,7a'R)-1'-(4-fluorophenyl)-2'-(3-methyl-4-nitroisoxazol-5-yl)-1',2',5',6',7',7a'-hexahydrospiro[indoline-3,3'-pyrrolizin]-2-one; White solid. M. P. 223-225 °C. IR (KBr) ν_{max} (cm^{-1}) 3154, 3083, 3024, 2944, 1715, 1593, 1512, 1488, 1441, 1411, 1295, 1142, 1010, 907, 824, 752, 676, 542; ^{1}H NMR (300 MHz, $CDCl_3$+DMSO- d_6) δ 10.16 (s, 1H), 7.50 (dd, J = 8.3, 5.1 Hz, 2H), 7.13 (tt, J = 8.0, 4.0 Hz, 1H), 7.02 (t, J = 8.6 Hz, 2H), 6.86 – 6.76 (m, 3H), 4.99 (d, J = 11.3 Hz, 1H), 4.39 – 4.30 (m, 1H), 3.92 (t, J = 10.7 Hz, 1H), 3.05 – 2.96 (m, 1H), 2.72 – 2.63 (m, 1H), 2.11 – 1.98 (m, 2H), 1.96 – 1.70 (m, 2H). ^{13}C NMR (75 MHz, $CDCl_3$+DMSO- d_6) δ 178.95, 170.91, 162.56, 159.31, 154.58, 141.93, 133.45, 128.86, 128.67, 128.57, 124.92, 123.85, 120.05, 114.89, 114.61, 109.49, 73.17, 70.17, 55.10, 51.14, 47.72, 27.33, 24.74, 10.30. HRMS (ESI+): m/z calculated for [$C_{24}H_{21}FN_4O_4$ +H+]: 449.1620; found: 449.2138.

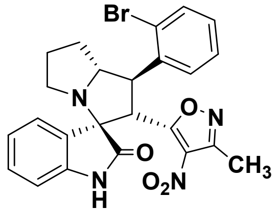

6d

(1'R,2'S,3R,7a'R)-1'-(2-bromophenyl)-2'-(3-methyl-4-nitroisoxazol-5-yl)-1',2',5',6',7',7a'-hexahydrospiro[indoline-3,3'-pyrrolizin]-2-one: White solid. M. P. 219-221 °C. IR (KBr) ν_{max} (cm^{-1}) 3292, 2979, 1729, 1594, 1517, 1471, 1450, 1412, 1246, 1145, 1018, 902, 829, 759, 701, 550; ^{1}H NMR (300 MHz, $CDCl_3$+DMSO-d_6) δ 10.08 (s, 1H), 7.89 (d, J = 6.9 Hz, 1H), 7.54 (d, J = 7.2 Hz, 1H), 7.36 (t, J = 7.2 Hz, 1H), 7.19 – 7.06 (m, 2H), 6.89 – 6.77 (m, 3H), 5.06 (d, J =

11.0 Hz, 1H), 4.74 (t, J = 10.5 Hz, 1H), 4.34 (dt, J = 10.1, 5.2 Hz, 1H), 3.09 (dd, J = 16.5, 8.0 Hz, 1H), 2.75 – 2.66 (m, 1H), 2.31 (s, 3H), 2.13 – 1.93 (m, 4H). ^{13}C NMR (75 MHz, $CDCl_3$+DMSO- d_6) δ 179.65, 171.68, 155.18, 142.49, 137.73, 132.62, 129.50, 128.62, 128.33, 127.96, 125.61, 124.91, 124.45, 120.80, 110.19, 74.29, 72.30, 55.54, 50.03, 48.60, 27.46, 25.07, 10.98. HRMS (ESI+): m/z calculated for [$C_{24}H_{21}BrN_4O_4$+H+]: 509.0819; found: 509.0840.

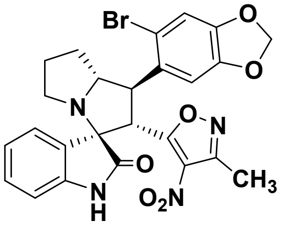

6e

(1'R,2'S,3R,7a'R)-1'-(6-bromobenzo[d][1,3]dioxol-5-yl)-2'-(3-methyl-4-nitroisoxazol-5-yl)-1',2',5',6',7',7a'-hexahydrospiro[indoline-3,3'-pyrrolizin]-2-one: White solid. M. P. 209-211 °C. IR (KBr) ν_{max} (cm^{-1}) 3295, 2982, 1732, 1597, 1520, 1474, 1453, 1415, 1249, 1148, 1021, 905, 832, 762, 703, 553; ^{1}H NMR (300 MHz, $CDCl_3$+DMSO- d_6) δ 10.19 (s, 1H), 7.40 (s, 1H), 7.19 – 7.10 (m, 1H), 6.96 (s, 1H), 6.84 – 6.77 (m, 3H), 5.98 (d, J = 8.2 Hz, 2H), 4.91 (d, J = 10.8 Hz, 1H), 4.65 (t, J = 10.4 Hz, 1H), 4.28 (ddd, J = 9.9, 6.4, 3.7 Hz, 1H), 3.08 (dd, J = 16.4, 7.9 Hz, 1H), 2.73 – 2.66 (m, 1H), 2.32 (s, 3H). 2.14 – 1.82 (m, 4H). ^{13}C NMR (101 MHz, $CDCl_3$+DMSO- d_6) δ 179.71, 171.89, 155.27, 147.92, 147.21, 142.55, 130.66, 129.92, 129.56, 125.62, 124.48, 120.84, 114.92, 112.20, 110.25, 107.89, 101.59, 74.30, 71.80, 55.33, 50.30, 48.77, 27.35, 24.97, 11.07. HRMS (ESI+): m/z calculated for [$C_{25}H_{22}BrN_4O_6$+H+]: 553.0717; found 553.0750.

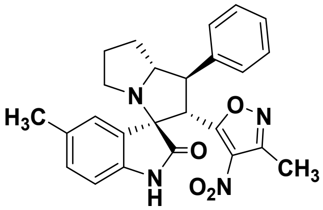

6f

(1'R,2'S,3R,7a'R)-5-methyl-2'-(3-methyl-4-nitroisoxazol-5-yl)-1'-phenyl-1',2',5',6',7',7a'-hexahydrospiro[indoline-3,3'-pyrrolizin]-2-one; White solid. M.P. 209-211 °C. IR (KBr) ν_{max} (cm^{-1}) 3180, 3082, 2969, 1724, 1598, 1521, 1489, 1419, 1378, 1277, 1179, 1147, 915, 828, 708, 599, 528; ^{1}H NMR (300 MHz, $CDCl_3$+DMSO- d_6) δ 9.98 (s, 1H), 7.56 – 7.48 (m, 3H), 7.32 (t, J = 7.4 Hz, 2H), 7.24 (t, J = 7.3 Hz, 1H), 6.93 (d, J = 7.8 Hz, 1H), 6.68 (d, J = 7.9 Hz, 1H), 6.62 (s,

1H), 5.04 (d, J = 11.3 Hz, 1H), 4.38 (ddd, J = 10.3, 6.4, 4.3 Hz, 1H), 3.92 (t, J = 10.7 Hz, 1H), 3.07 – 2.95 (m, 2H), 2.72 – 2.62 (m, 1H), 2.30 (s, 3H), 2.20 (s, 3H), 2.12 – 1.74 (m, 5H).^{13}C NMR (75 MHz, $CDCl_3$+DMSO-d_6) δ 179.76, 171.87, 155.15, 139.89, 138.20, 129.98, 129.68, 128.49, 127.69, 127.00, 126.20, 124.65, 109.75, 74.00, 70.93, 55.83, 52.51, 48.30, 28.06, 25.43, 20.60, 10.87. HRMS (ESI+): m/z calculated for [$C_{25}H_{24}N_4O_4$+H+]: 445.1876; found 445.1891.

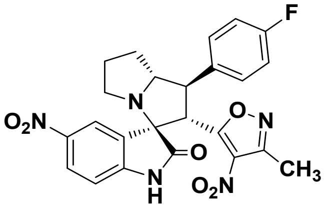

6g

(1'S,2'R,3R,7a'R)-1'-(4-fluorophenyl)-2'-(3-methyl-4-nitroisoxazol-5-yl)-5-nitro-1',2',5',6',7',7a'-hexahydrospiro[indoline-3,3'-pyrrolizin]-2-one; Faint yellow solid. M.P. 212-214 °C. IR (KBr) ν_{max} (cm^{-1}) 3092, 2967, 1755, 1601, 1513, 1474, 1417, 1330, 1260, 1144, 1016, 904, 832, 790, 671, 530; ^{1}H NMR (300 MHz, $CDCl_3$+DMSO-d_6) δ 11.12 (s, 1H), 8.13 (dd, J = 8.7, 2.2 Hz, 1H), 7.71 (d, J = 2.1 Hz, 1H), 7.50 (dd, J = 8.6, 5.3 Hz, 2H), 7.04 (t, J = 8.7 Hz, 2H), 6.95 (d, J = 8.7 Hz, 1H), 5.00 (d, J = 11.3 Hz, 1H), 4.35 – 4.26 (m, 1H), 3.96 (t, J = 10.7 Hz, 1H), 2.93 (dd, J = 16.3, 7.7 Hz, 1H), 2.70 – 2.63 (m, 1H), 2.31 (s, 3H), 2.15 – 1.79 (m, 4H). ^{13}C NMR (75 MHz, $CDCl_3$+DMSO-d_6) δ 179.25, 170.18, 162.97, 159.71, 154.95, 148.58, 141.24, 132.99, 129.64, 128.88, 128.78, 126.17, 125.05, 120.71, 115.28, 115.00, 109.68, 72.52, 70.50, 55.51, 51.38, 47.54, 27.56, 25.35, 10.50. HRMS (ESI+): m/z calculated for [$C_{24}H_{21}FN_5O_6$+H+]: 494.1470; found 494.1473.

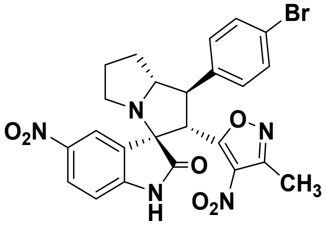

6h

(1'R,2'S,3R,7a'R)-1'-(4-bromophenyl)-2'-(3-methyl-4-nitroisoxazol-5-yl)-5-nitro-1',2',5',6',7',7a'-hexahydrospiro[indoline-3,3'-pyrrolizin]-2-one: White solid. M.P. 218-220 °C. IR (KBr) ν_{max} (cm^{-1}) 3091, 2964, 1760, 1627, 1511, 1475, 1327, 1179, 1142, 1077, 904, 756, 680, 524. ^{1}H NMR (500 MHz, $CDCl_3$) δ 8.19 (d, J = 8.5 Hz, 1H), 7.97 (s, 1H), 7.74 (s, 1H), 7.48 (d, J = 8.2 Hz, 2H), 7.39 (d, J = 7.9 Hz, 2H), 6.94 (d, J = 8.6 Hz, 1H), 5.00 (d, J = 11.3 Hz, 1H),

4.38 – 4.31 (m, 1H), 3.93 (t, J = 10.6 Hz, 1H), 2.93 (dd, J = 16.3, 8.2 Hz, 1H), 2.67 (t, J = 8.6 Hz, 1H), 2.32 (s, 3H), 2.19 – 1.93 (m, 4H). ^{13}C NMR (126 MHz, $CDCl_3$) δ 180.04, 170.73, 155.69, 149.07, 141.99, 136.81, 132.08, 129.69, 126.86, 125.67, 121.51, 121.42, 110.34, 73.29, 71.10, 56.22, 52.37, 48.29, 28.29, 26.01, 11.24. HRMS (ESI+): m/z calculated for [$C_{24}H_{21}BrN_4O_4$+H+]: 509.0819; found: 509.0833.

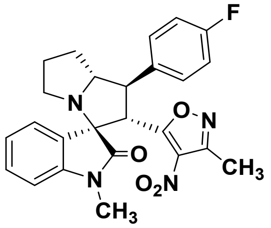

6i

(1'R,2'S,3R,7a'R)-1'-(4-fluorophenyl)-1-methyl-2'-(3-methyl-4-nitroisoxazol-5-yl)-1',2',5',6',7',7a'-hexahydrospiro[indoline-3,3'-pyrrolizin]-2-one; White solid. Yield 80%. M.P. 178-180 °C. IR (KBr) ν_{max} (cm^{-1}) 3431, 2939, 1725, 1604, 1512, 1469, 1418, 1225, 1144, 1105, 906, 830, 782, 695, 537; ^{1}H NMR (400 MHz, $CDCl_3$) δ 7.55 – 7.50 (m, 2H), 7.26 – 7.21 (m, 1H), 7.05 – 6.99 (m, 2H), 6.83 (td, J = 7.5, 1.0 Hz, 1H), 6.77 – 6.74 (m, 2H), 4.89 (d, J = 10.9 Hz, 1H), 4.47 (ddd, J = 10.4, 6.9, 3.7 Hz, 1H), 3.93 (t, J = 10.5 Hz, 1H), 3.23 (s, 3H), 3.06 (td, J = 9.0, 6.8 Hz, 1H), 2.71 – 2.64 (m, 1H), 2.30 (s,3H), 2.09 – 2.01 (m, 2H), 1.96 – 1.86 (m, 1H), 1.80 – 1.71 (m, 1H). ^{13}C NMR (101 MHz, $CDCl_3$) δ 178.58, 172.66, 156.01, 144.81, 134.38, 130.22, 129.93, 129.85, 125.74, 124.71, 121.83, 116.13, 115.92, 108.78, 73.99, 71.33, 57.03, 52.26, 49.29, 28.00, 26.64, 25.44, 11.48. HRMS (ESI+): m/z calculated for [$C_{24}H_{23}FN_5O_4$+H+]: 463.1782; found 463.1790.

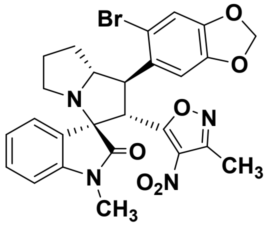

6j

(1'R,2'S,3R,7a'R)-1'-(6-bromobenzo[d][1,3]dioxol-5-yl)-1-methyl-2'-(3-methyl-4-nitroisoxazol-5-yl)-1',2',5',6',7',7a'-hexahydrospiro[indoline-3,3'-pyrrolizin]-2-one: White solid. M.P. 196-198 °C. IR (KBr) ν_{max} (cm^{-1}) 3401, 2962, 1709, 1603, 1501, 1475, 1415, 1376, 1238, 1123, 1102, 923, 751, 684, 538; ^{1}H NMR (400 MHz, $CDCl_3$) δ 7.48 (s, 1H), 7.24 (ddd, J = 9.1, 5.9, 1.3 Hz, 1H), 6.96 (s, 1H), 6.86 – 6.81 (m, 1H), 6.79 – 6.75 (m, 2H), 5.96 (dd, J = 14.2,

1.4 Hz, 2H), 4.83 (d, *J* = 10.5 Hz, 1H), 4.70 (t, *J* = 10.2 Hz, 1H), 4.41 (ddd, *J* = 10.0, 6.9, 3.3 Hz, 1H), 3.24 (s, 3H), 3.21 – 3.12 (m, 1H), 2.72 (ddd, *J* = 9.2, 7.3, 2.8 Hz, 1H), 2.32 (s, 3H), 2.15 – 1.85 (m, 4H). ^{13}C NMR (126 MHz, $CDCl_3$) δ 178.48, 172.77, 155.87, 148.46, 147.69, 144.78, 131.14, 130.21, 125.85, 124.56, 121.86, 115.42, 112.65, 108.76, 108.59, 102.02, 74.41, 72.28, 56.27, 50.58, 49.64, 27.33, 26.61, 25.00, 11.48. HRMS (ESI+): m/z calculated for [$C_{26}H_{23}BrN_4O_6$+H+]: 567.0879; found 567.0900.

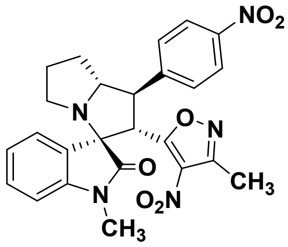

6k

(1'R,2'S,3R,7a'R)-1-methyl-2'-(3-methyl-4-nitroisoxazol-5-yl)-1'-(4-nitrophenyl)-1',2',5',6',7',7a'-hexahydrospiro[indoline-3,3'-pyrrolizin]-2-one; White solid. M. P. 182-184 ^{o}C. IR (KBr) ν_{max} (cm^{-1}) 3433, 3081, 2940, 1724, 1604, 1515, 1494, 1469, 1418, 1349, 1145, 1105, 904, 831, 759, 691, 544; ^{1}H NMR (400 MHz, $CDCl_3$) δ 8.22 – 8.18 (m, 2H), 7.79 – 7.74 (m, 2H), 7.29 – 7.23 (m, 1H), 6.84 (td, *J* = 7.6, 0.9 Hz, 1H), 6.78 (t, *J* = 7.1 Hz, 1H), 6.72 (dd, *J* = 7.5, 0.7 Hz, 1H), 4.94 (d, *J* = 10.7 Hz, 1H), 4.55 (ddd, *J* = 10.3, 7.0, 3.6 Hz, 1H), 4.06 (t, *J* = 10.4 Hz, 1H), 3.25 (s, 3H), 3.09 (td, *J* = 9.0, 6.9 Hz, 1H), 2.74 – 2.68 (m, 1H), 2.32 (s, 3H), 2.13 – 2.03 (m, 1H), 2.00 – 1.89 (m, 1H), 1.81 – 1.72 (m, 1H). ^{13}C NMR (101 MHz, $CDCl_3$) δ 178.39, 172.11, 156.15, 147.55, 146.68, 144.82, 130.44, 129.35, 125.63, 124.38, 121.94, 108.95, 74.23, 71.45, 56.96, 52.74, 49.30, 27.88, 26.66, 25.30, 11.44. HRMS (ESI+): m/z calculated for [$C_{25}H_{23}FN_5O_6$+H+]: 490.1727; found 490.1728.

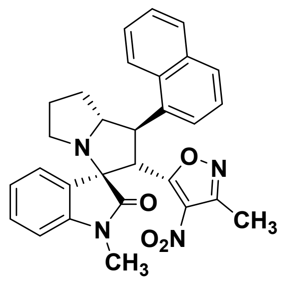

6l

(1'R,2'S,3R,7a'R)-1-methyl-2'-(3-methyl-4-nitroisoxazol-5-yl)-1'-(naphthalen-1-yl)-1',2',5',6',7',7a'-hexahydrospiro[indoline-3,3'-pyrrolizin]-2-one; White solid. M. P. 182-184 °C. IR (KBr) ν_{max} (cm^{-1})^{1}H NMR (500 MHz, $CDCl_3$) δ 8.47 (d, *J* = 7.9 Hz, 1H), 8.12 (d, *J* = 6.5 Hz, 1H), 7.86 (d, *J* = 7.9 Hz, 1H), 7.76 (d, *J* = 8.3 Hz, 1H), 7.61 (t, *J* = 7.2 Hz, 1H), 7.52 (dt, *J* = 25.7, 7.5 Hz, 2H), 7.25 – 7.22 (m, 1H), 6.87 (s, 2H), 6.78 (d, *J* = 7.6 Hz, 1H), 5.20 (d, *J* = 10.5 Hz, 1H), 4.96 (t, *J* = 10.0 Hz, 1H), 4.71 (m, 1H), 3.30 – 3.21 (m, 4H), 2.77 (t, *J* = 7.5 Hz, 1H), 2.24 (s, 3H), 2.20 – 2.09 (m, 1H), 2.05 – 1.89 (m, 2H), 1.83 – 1.74 (m, 1H). ^{13}C NMR (126 MHz, $CDCl_3$) δ 178.71, 173.12, 155.93, 144.82, 134.01, 132.51, 130.22, 129.16, 127.82, 126.66, 126.18, 125.84, 125.81, 124.83, 122.71, 121.89, 108.82, 74.41, 49.44, 28.27, 26.67, 25.49, 11.45. HRMS (ESI+): m/z calculated for [$C_{29}H_{27}N_4O_4$+H+]: 495.2032; found 495.2138.

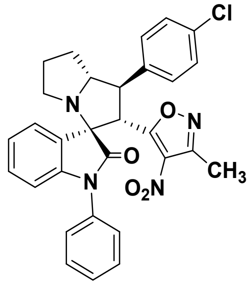

6m

(1'R,2'S,3R,7a'R)-1'-(4-chlorophenyl)-2'-(3-methyl-4-nitroisoxazol-5-yl)-1-phenyl-1',2',5',6',7',7a'-hexahydrospiro[indoline-3,3'-pyrrolizin]-2-one; White solid. M. P. 201-203 °C. IR (KBr) ν_{max} (cm^{-1}) 3059, 2926, 1725, 1598, 1498, 1462, 1374, 1323, 1202, 1147, 1088, 1012, 941, 825, 753, 698, 532; ^{1}H NMR (300 MHz, $CDCl_3$+DMSO-d_6) δ 7.58 – 7.45 (m, 7H), 7.30 (d, *J* = 8.4 Hz, 2H), 7.18 (t, *J* = 7.3 Hz, 1H), 6.88 (t, *J* = 7.5 Hz, 1H), 6.80 (d, *J* = 7.2 Hz, 1H), 6.69 (d, *J* = 7.9 Hz, 1H), 4.97 (d, *J* = 10.8 Hz, 1H), 4.49 – 4.38 (m, 1H), 3.99 (t, *J* = 10.5 Hz, 1H), 3.14 (dd, *J* = 15.9, 8.6 Hz, 1H), 2.81 – 2.70 (m, 1H), 2.34 (s, 3H), 2.19 – 1.76 (m, 4H). ^{13}C NMR (75 MHz, $CDCl_3$+DMSO) δ 177.14, 171.80, 155.25, 143.96, 136.70, 133.51, 132.52, 129.45, 129.07, 129.00, 128.48, 127.79, 126.25, 125.24, 123.63, 121.64, 109.26, 73.31, 70.53, 56.62, 51.34, 48.25, 27.12, 24.72, 10.72. HRMS (ESI+): m/z calculated for [$C_{30}H_{26}ClN_4O_4$+H+]: 541.1643; found 541.1655.

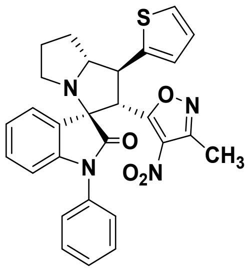

6n

(1'S,2'S,3R,7a'R)-2'-(3-methyl-4-nitroisoxazol-5-yl)-1-phenyl-1'-(thiophen-2-yl)-1',2',5',6',7',7a'-hexahydrospiro[indoline-3,3'-pyrrolizin]-2-one; White solid. M.P. 189-191 ^{o}C. IR (KBr) ν_{max} (cm^{-1}) 3064, 2930, 1739, 1595, 1512, 1462, 1415, 1372, 1283, 1151, 1114, 912, 827, 756, 698, 529; ^{1}H NMR (300 MHz, $CDCl_3$+DMSO-d_6) δ 7.58 – 7.50 (m, 2H), 7.46 (t, *J* = 6.1 Hz, 3H), 7.23 – 7.13 (m, 2H), 7.07 (d, *J* = 3.0 Hz, 1H), 6.97 – 6.92 (m, 1H), 6.91 – 6.86 (m, 2H), 6.69 (d, *J* = 7.9 Hz, 1H), 5.02 (d, *J* = 10.9 Hz, 1H), 4.46 (ddd, *J* = 10.4, 6.8, 3.8 Hz, 1H), 4.32 (t, *J* = 10.5 Hz, 1H), 3.09 (dd, *J* = 15.7, 8.8 Hz, 1H), 2.78 – 2.70 (m, 1H), 2.35 (s, 3H), 2.21 – 2.05 (m, 2H), 2.02 – 1.84 (m, 2H). ^{13}C NMR (75 MHz, $CDCl_3$+DMSO-d_6) δ 176.93, 171.43, 155.35, 144.00, 140.92, 133.70, 130.09, 129.56, 129.15, 127.83, 126.80, 126.34, 125.48, 124.77, 124.04, 123.74, 121.78, 109.36, 73.13, 70.68, 57.10, 48.14, 47.21, 27.69, 25.15, 10.86. HRMS (ESI+): m/z calculated for [$C_{28}H_{24}N_4O_4S$+H+]: 513.1597; found 513.1603.

Reference:

1. P. Disetti, M. Moccia, D.S. Illera, S. Suresh, M.F.A. Adamo. *Org. Biomol. Chem.*, **2015**, *13*, 10609–10612.

2. ^{1}H & ^{13}C Spectra of Spiro-Indenoquinoxaline Pyrrolizidines (4a-u)& Spirooxindoles Pyrrolizidines (6a-n)

^{1}H & ^{13}C Spectra of 4a

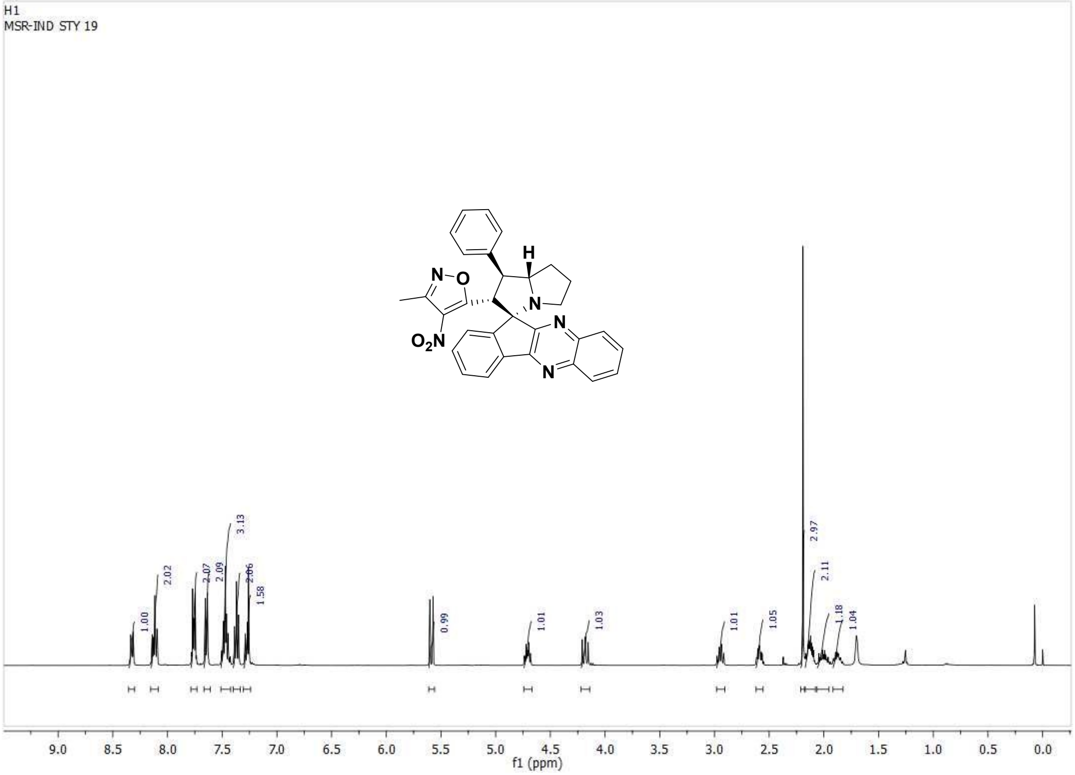

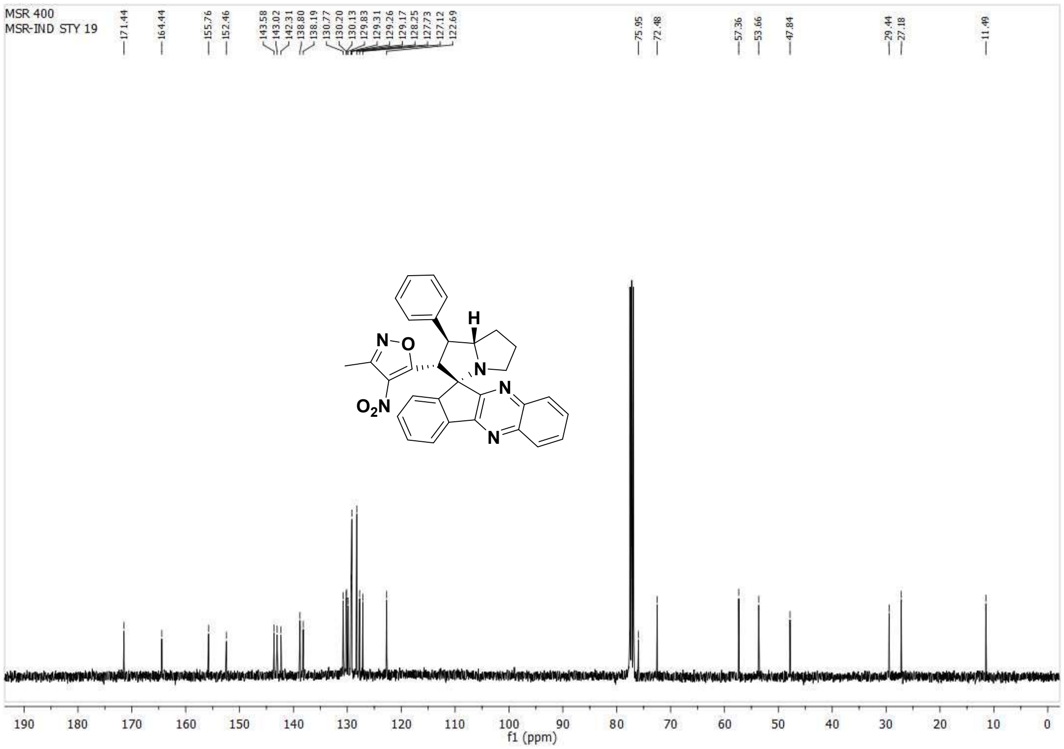

^{1}H & ^{13}C Spectra of 4b

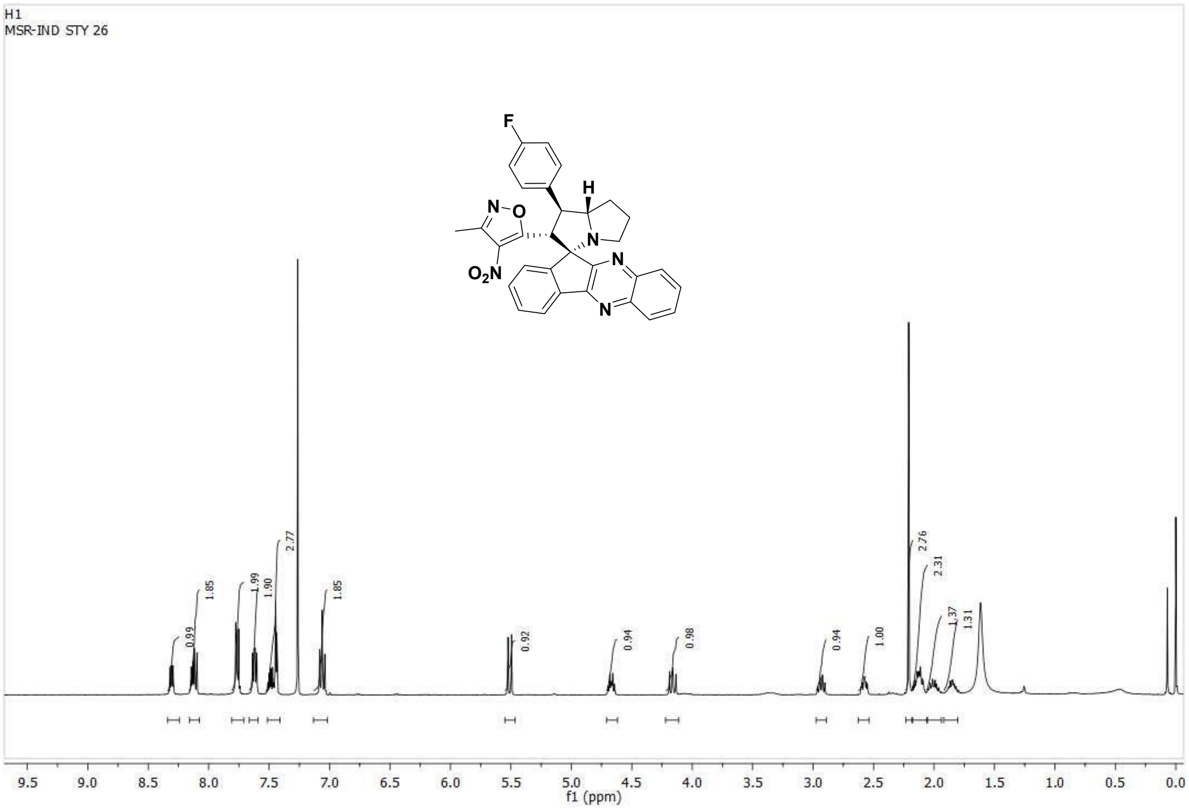

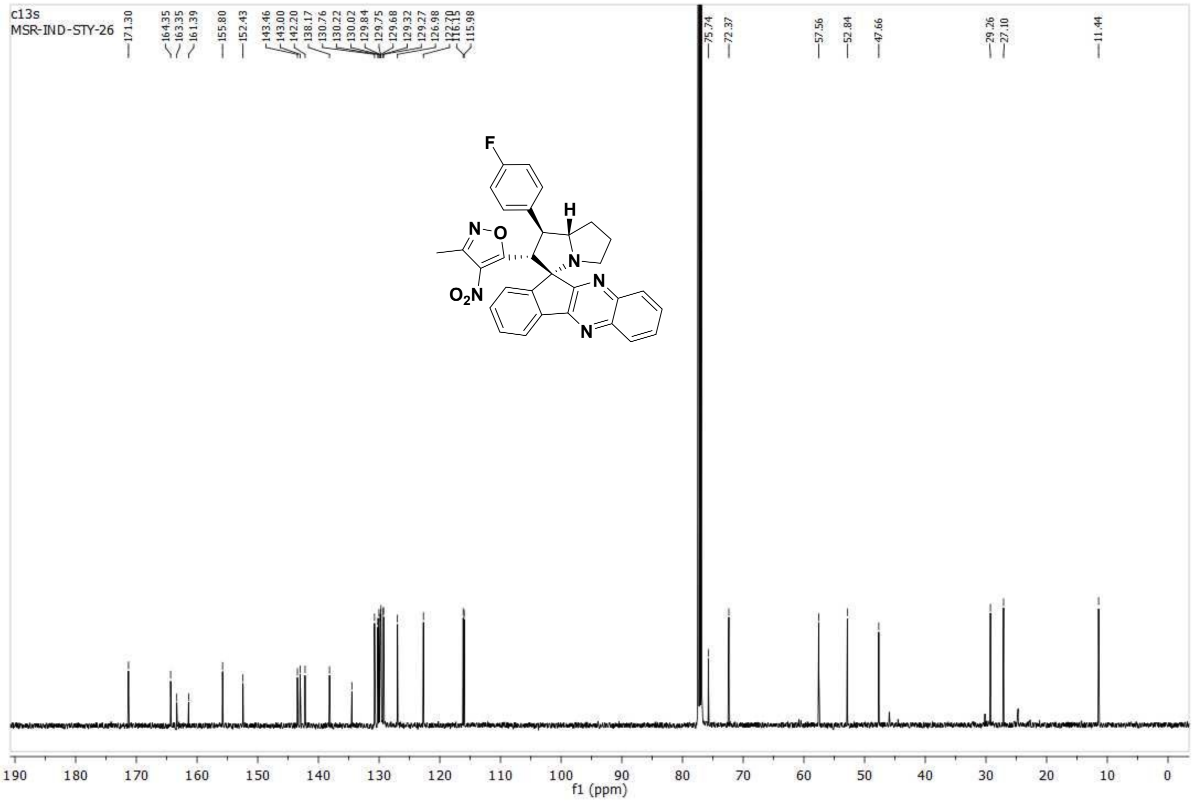

^{19}F Spectra of 4b

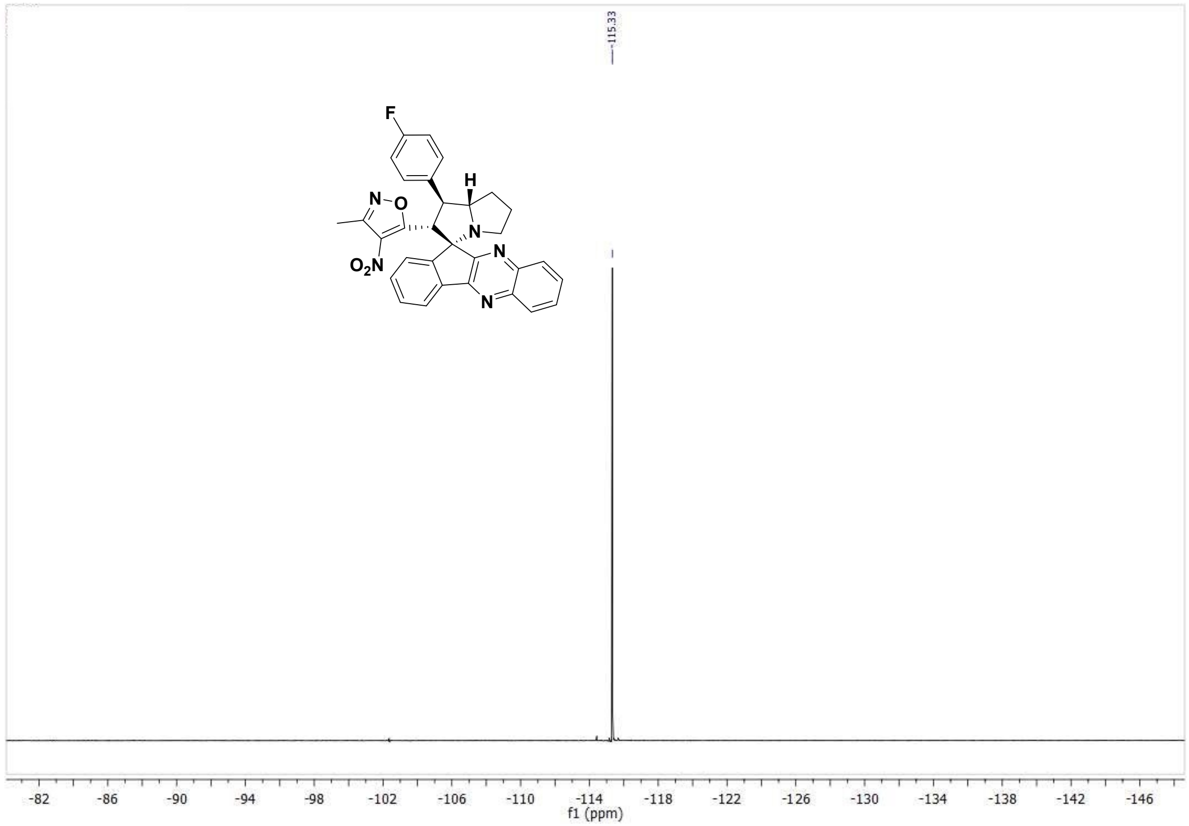

^{1}H & ^{13}C Spectra of 4c

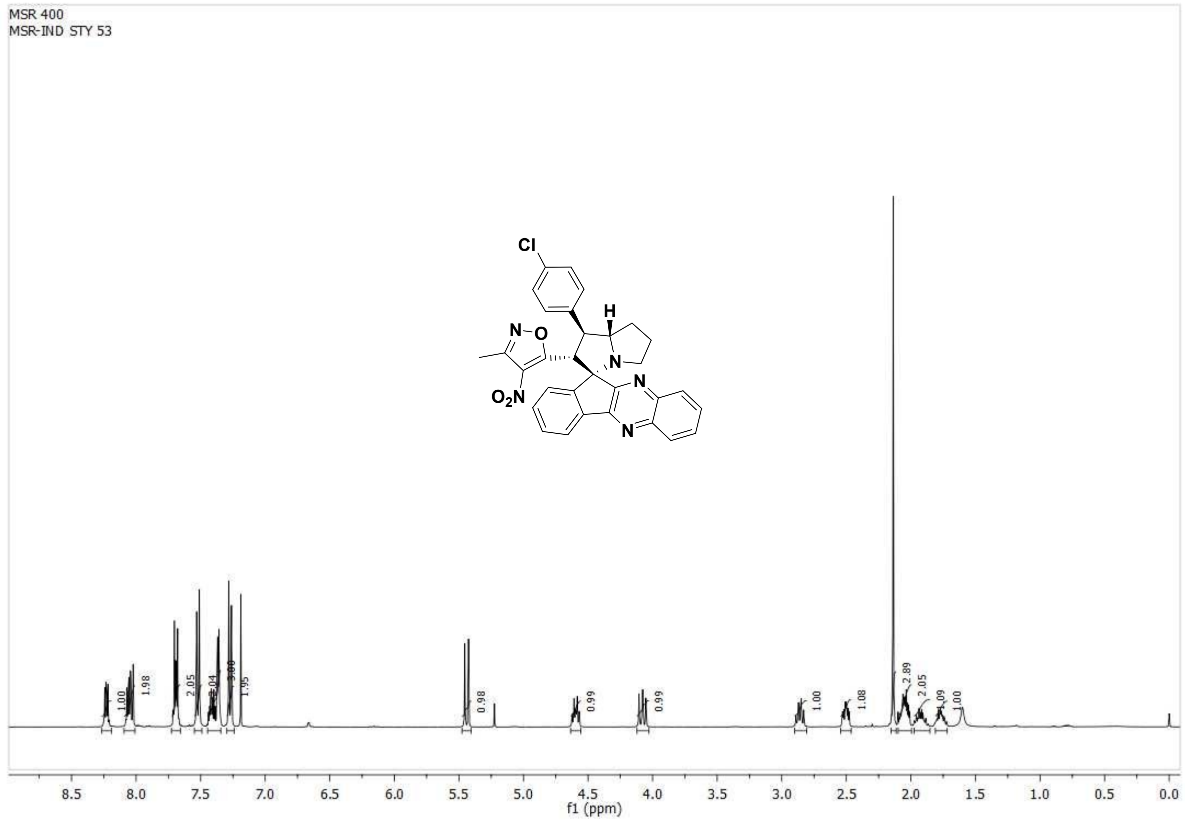

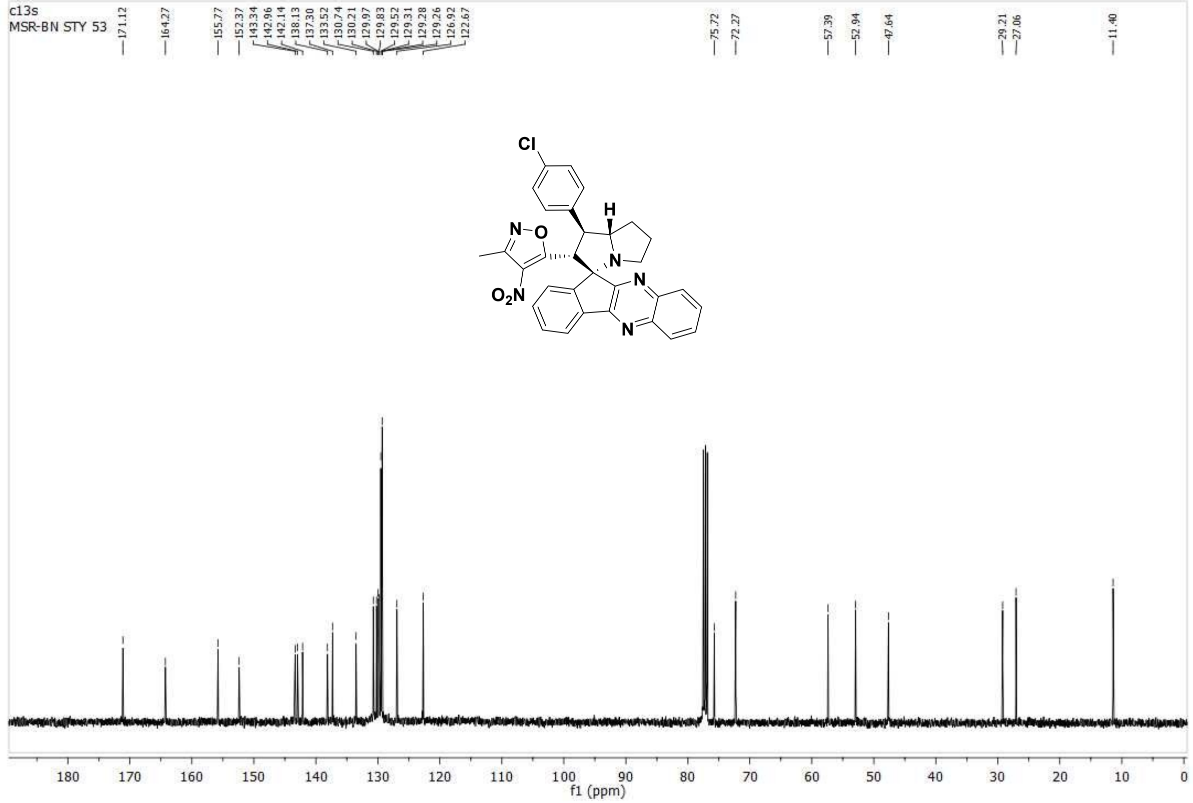

^{1}H &[13]C Spectra of 4d

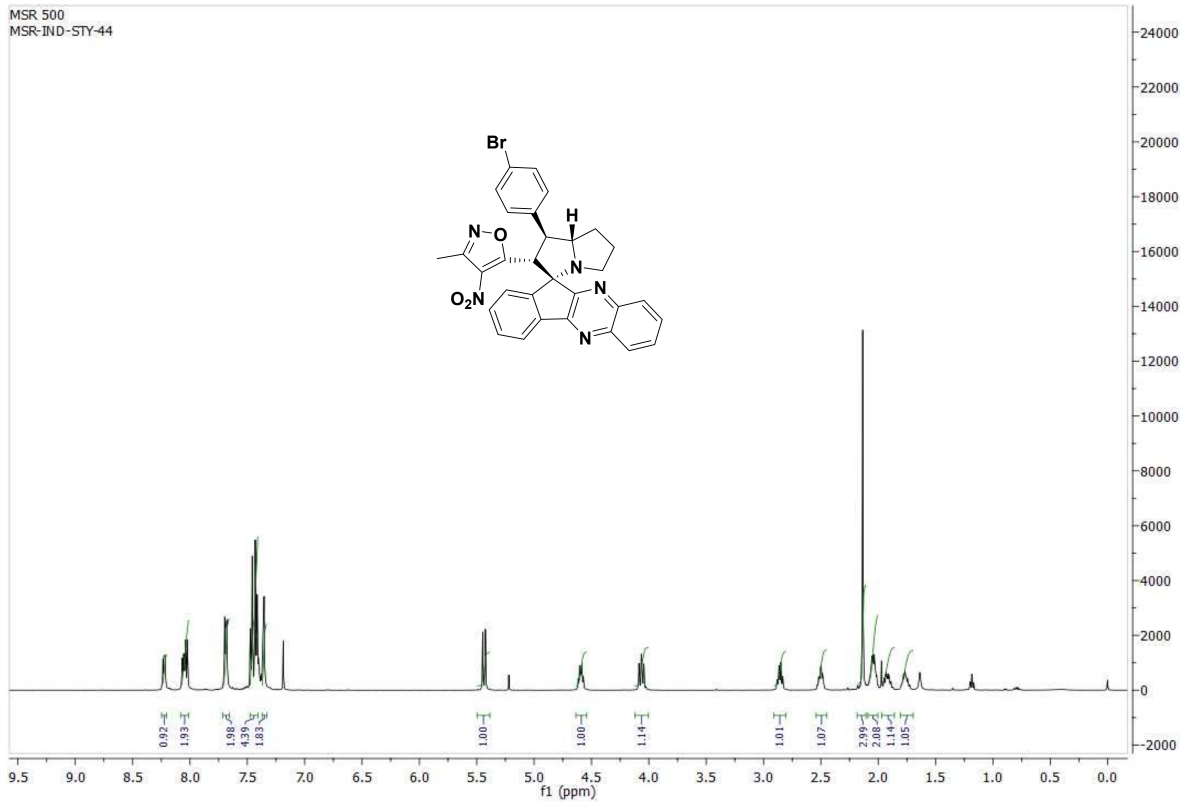

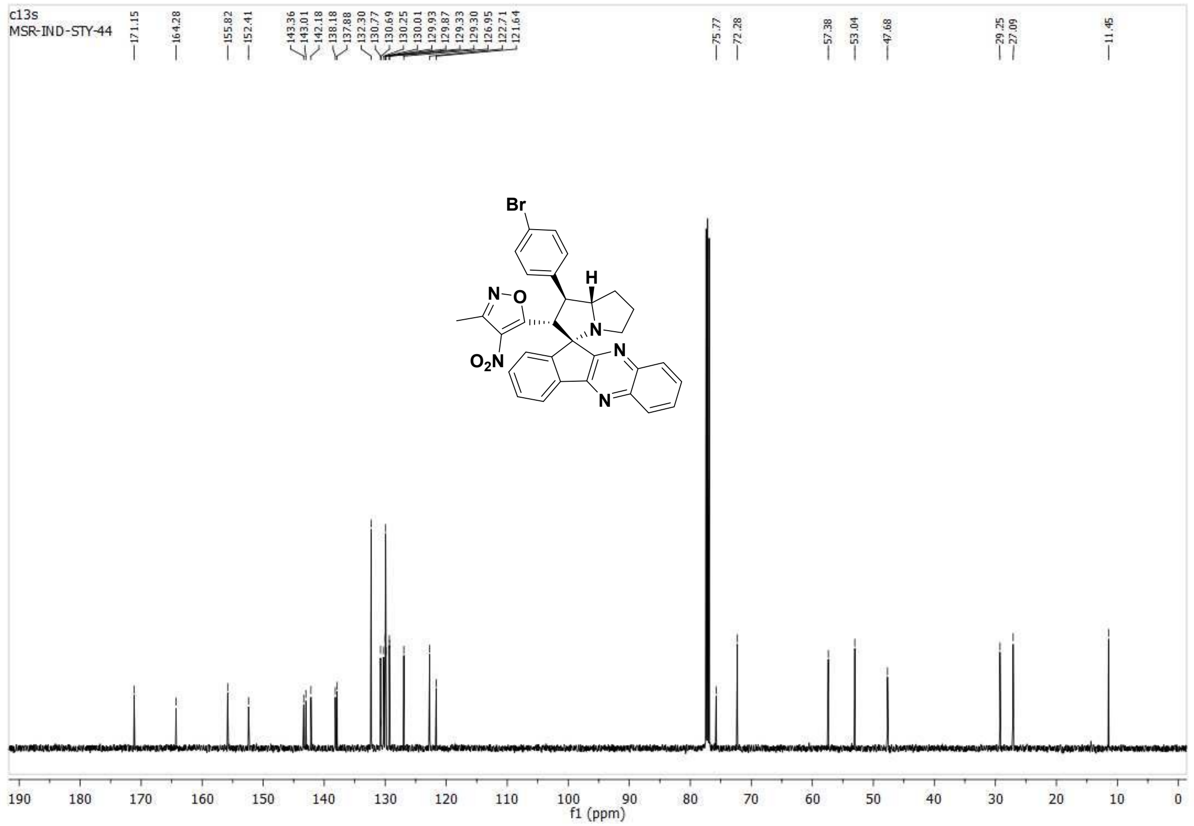

^{1}H & ^{13}C Spectra of 4e

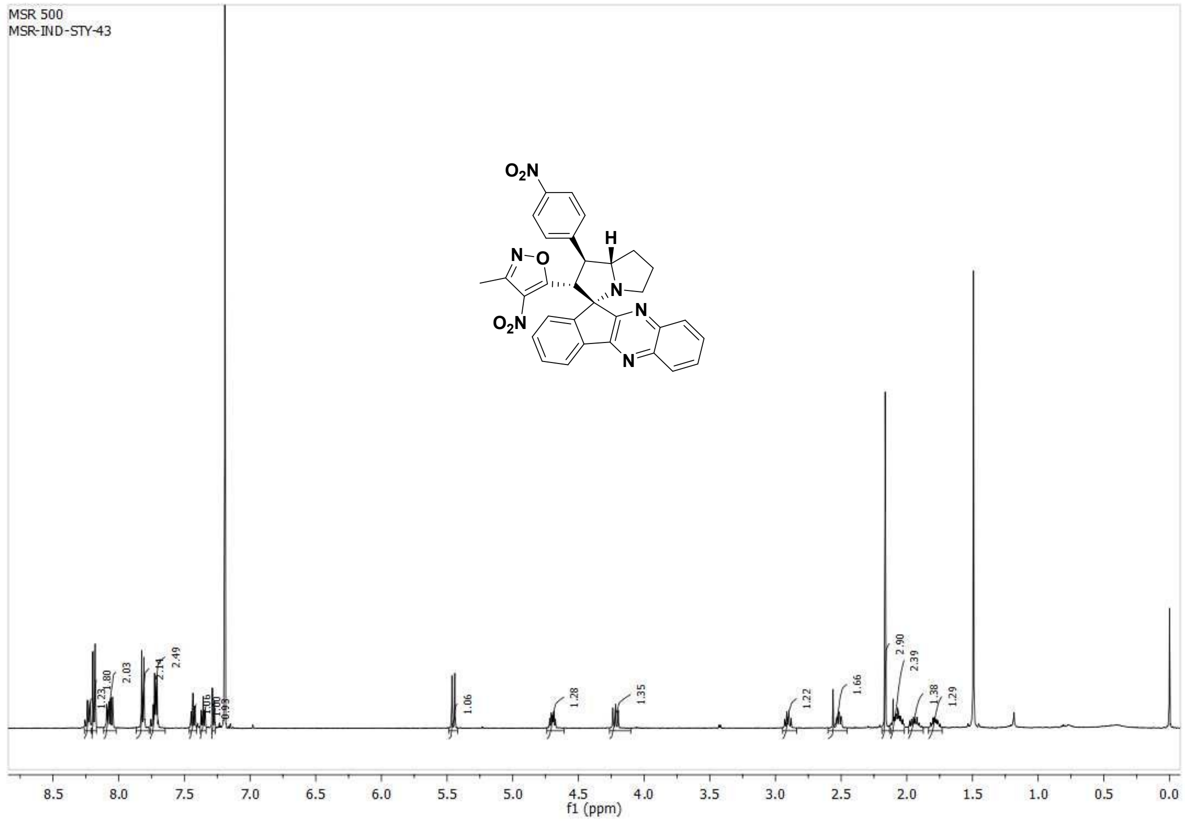

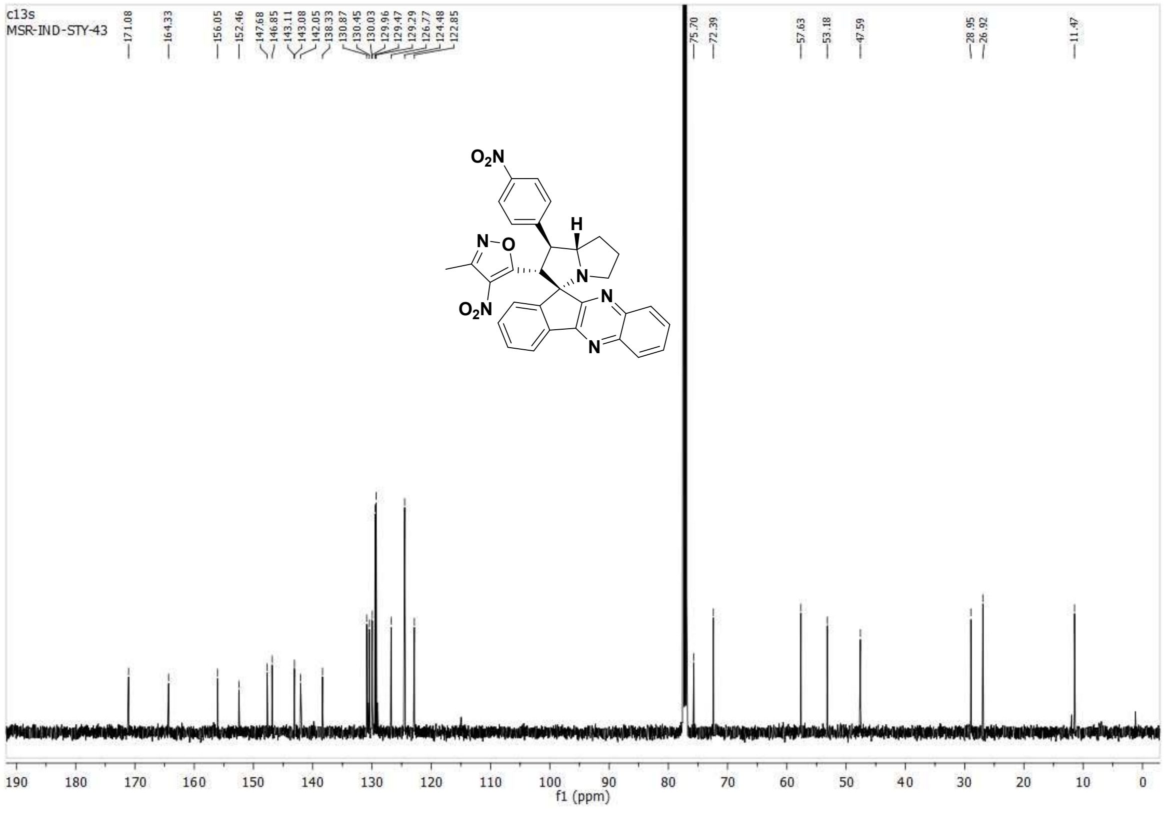

^{1}H &[13]C Spectra of 4f

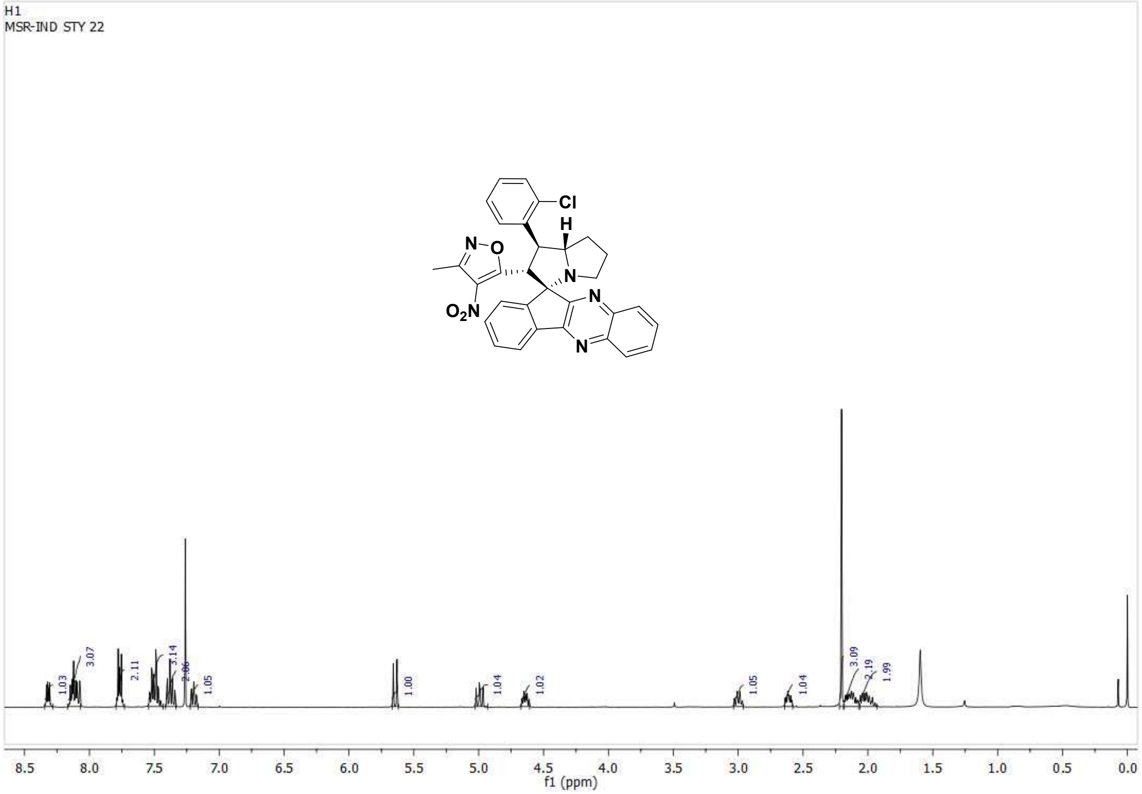

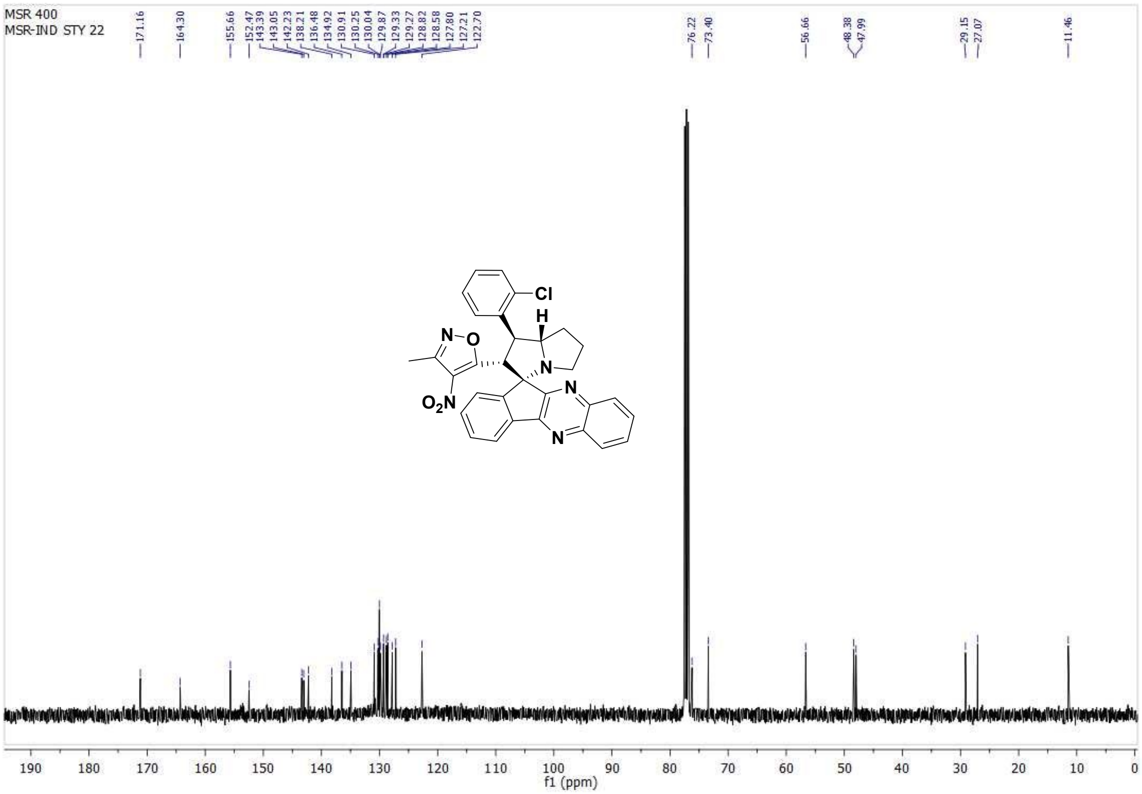

^{1}H &[13]C Spectra of 4g

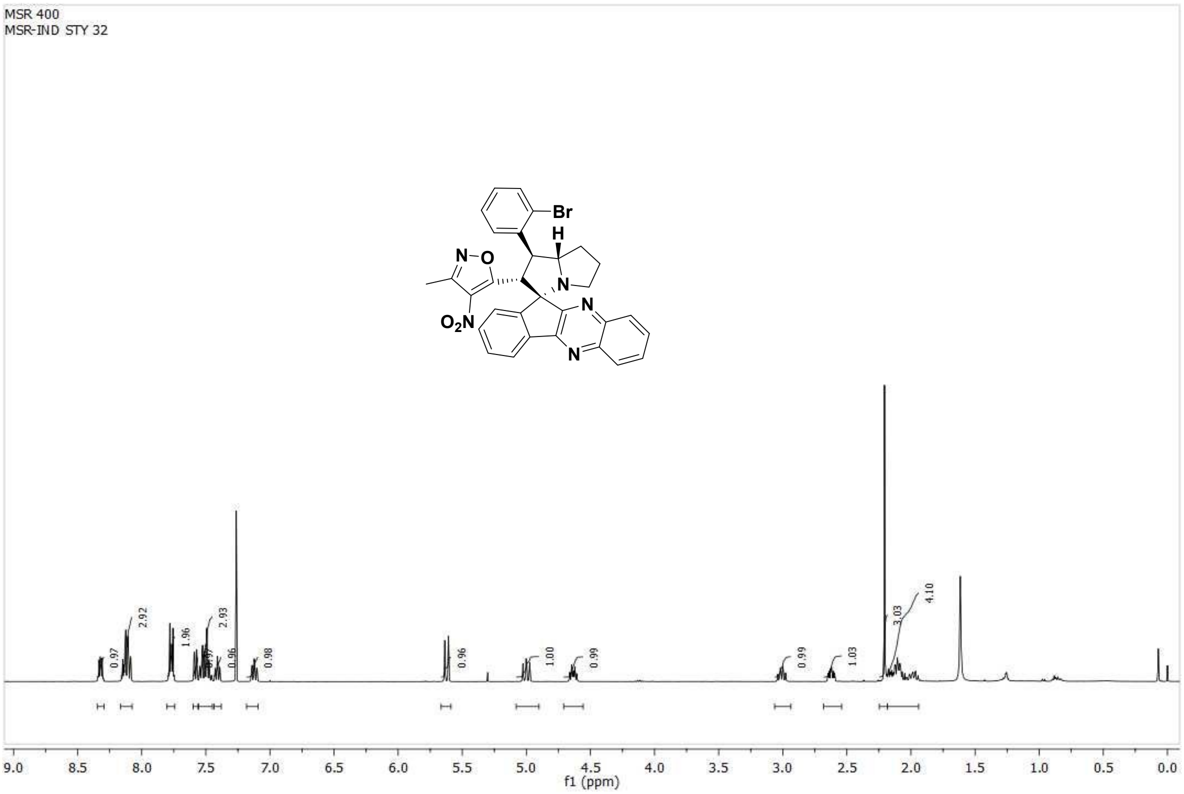

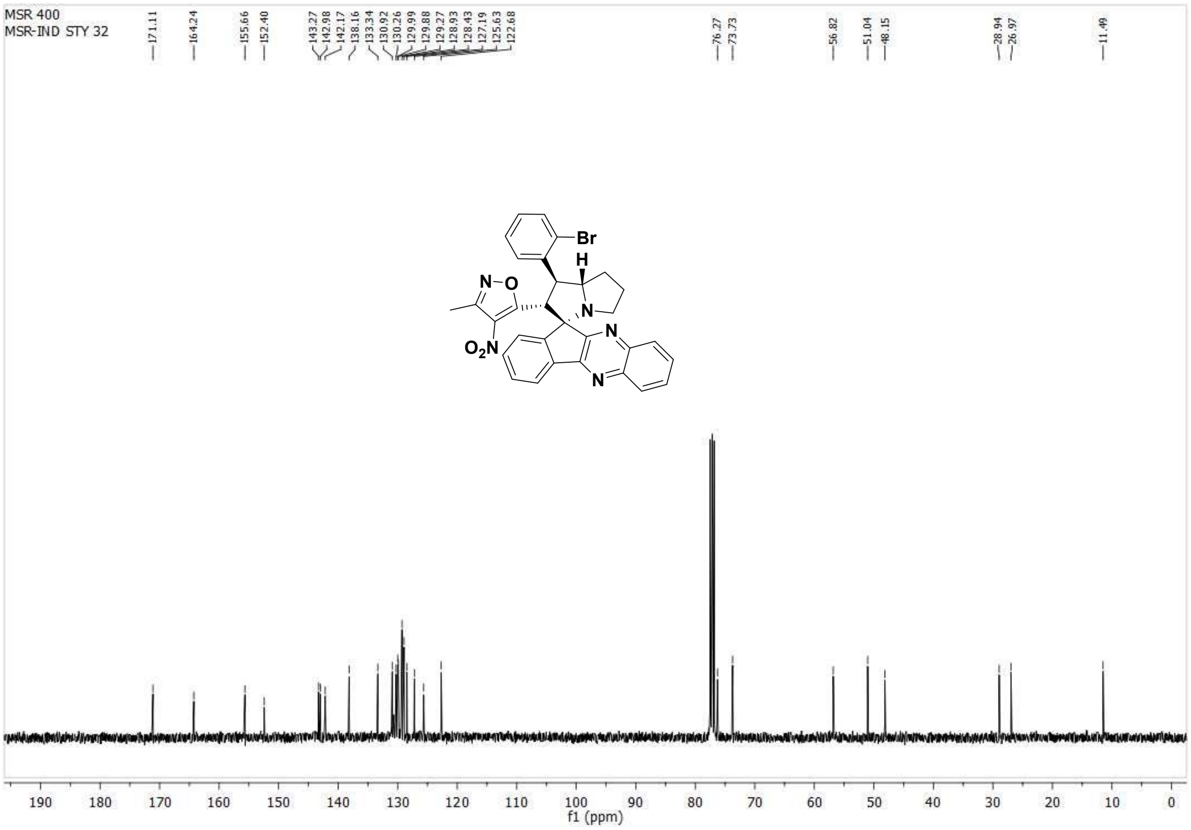

^{1}H &^{13}C Spectra of 4h

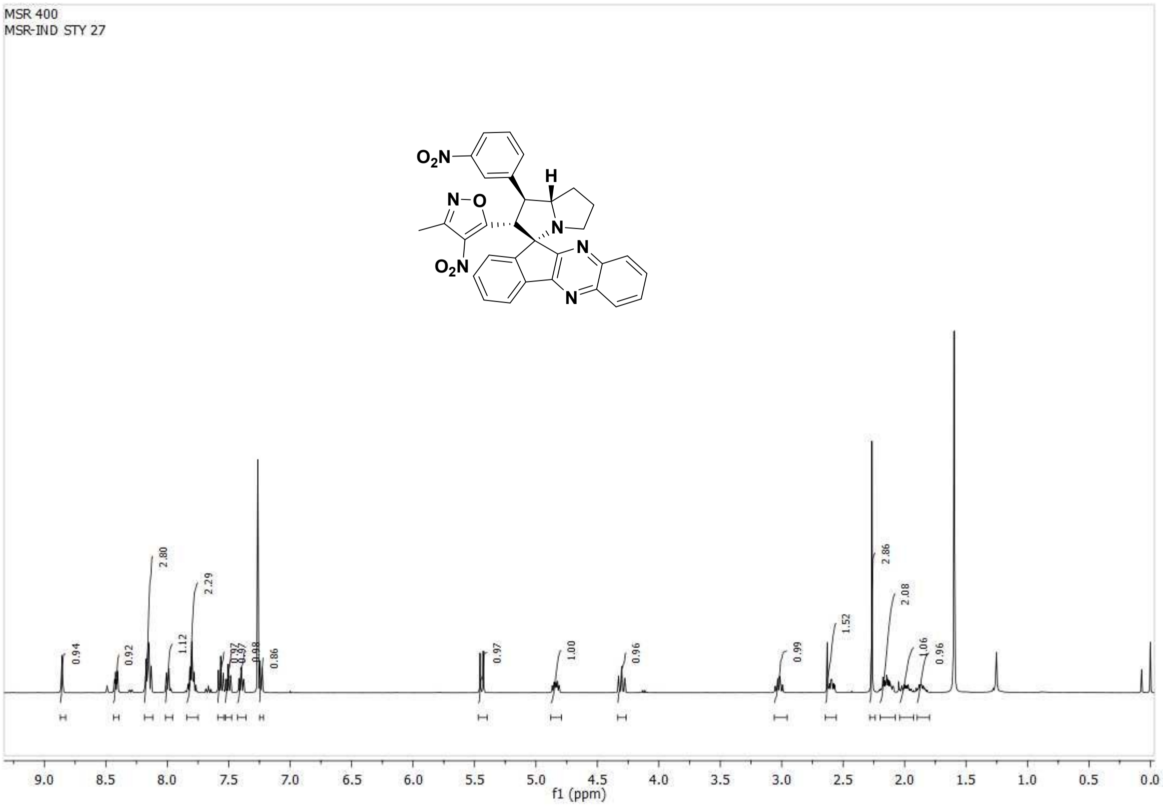

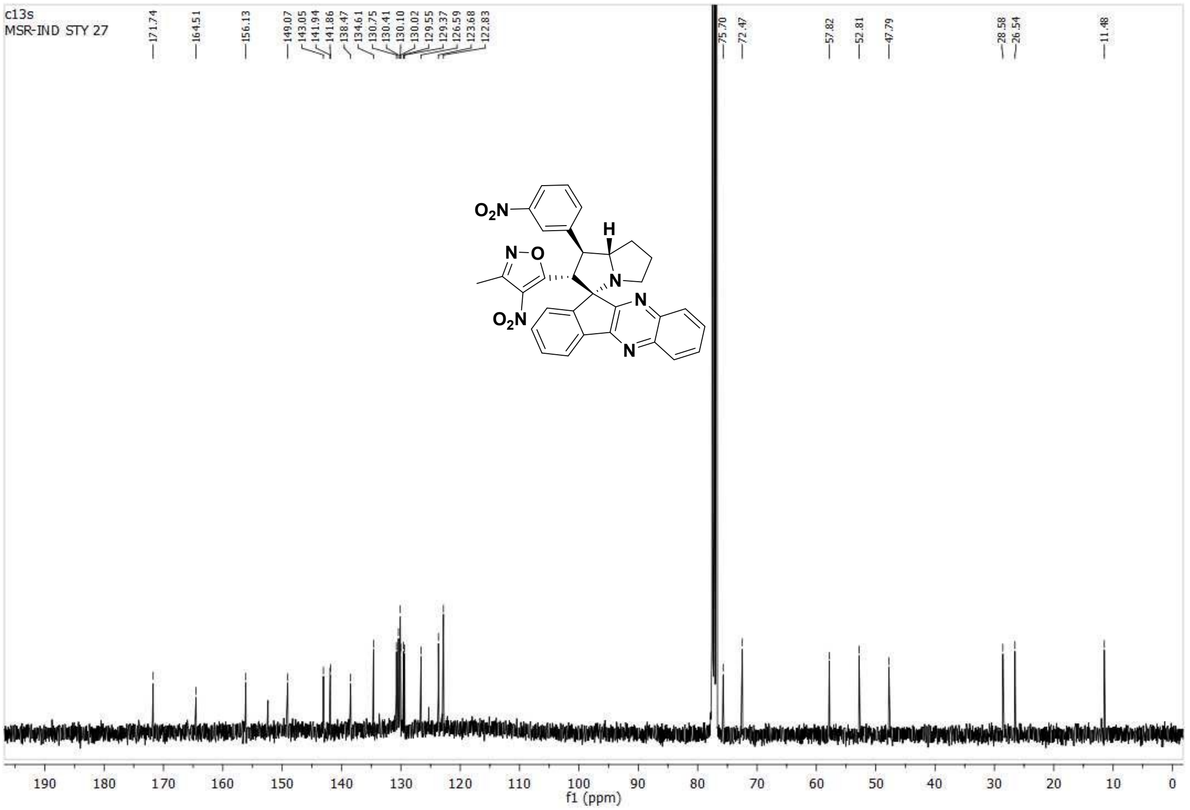

^{1}H & ^{13}C Spectra of 4i

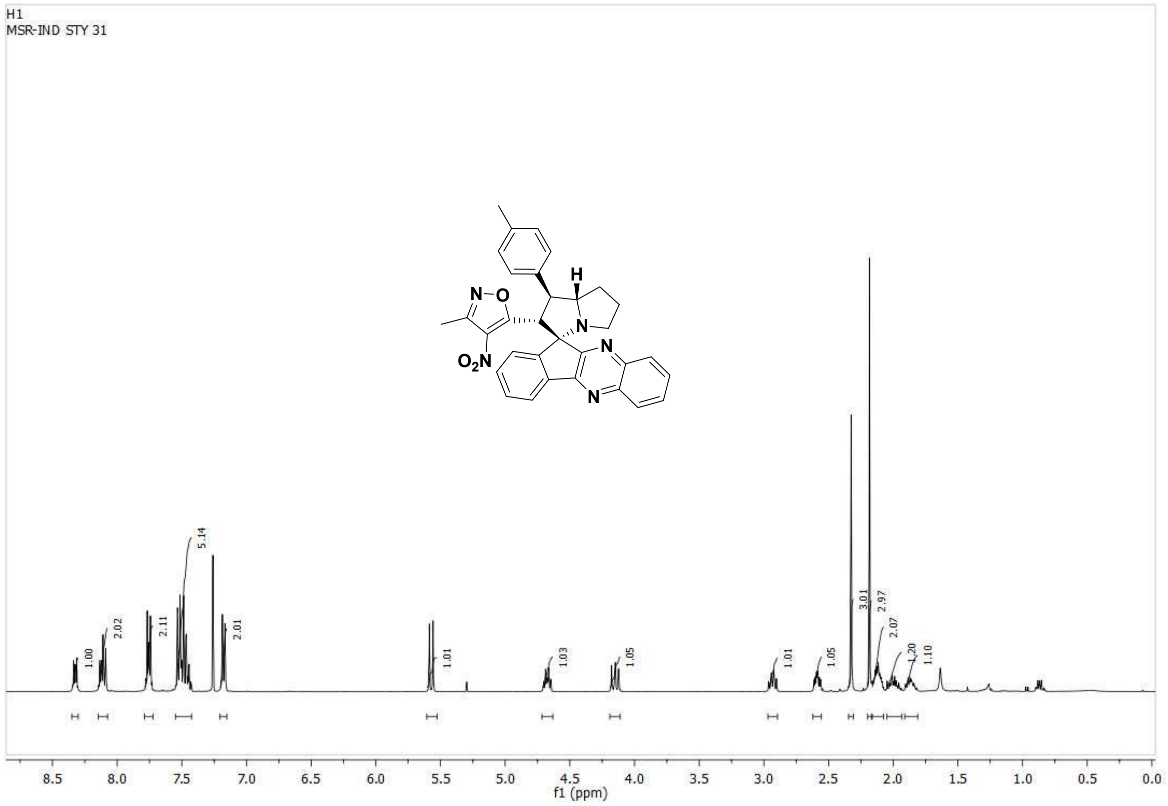

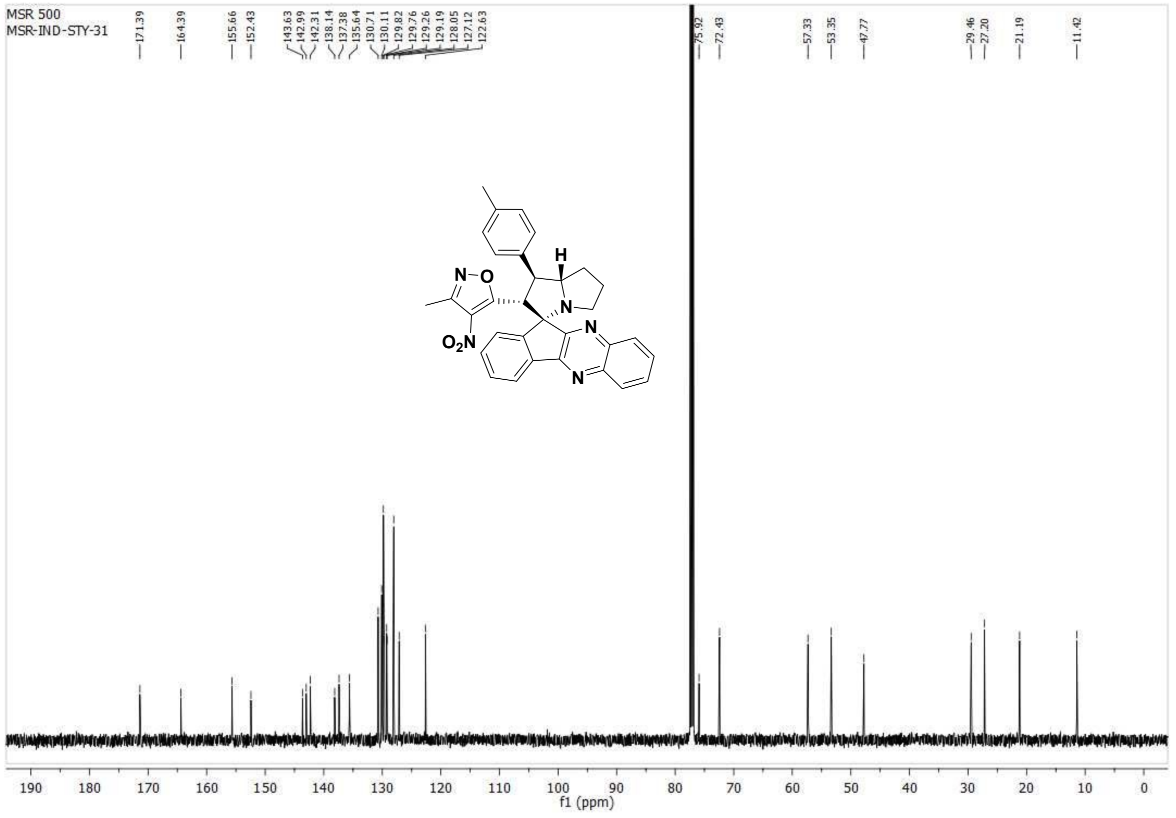

^{1}H & ^{13}C Spectra of 4j

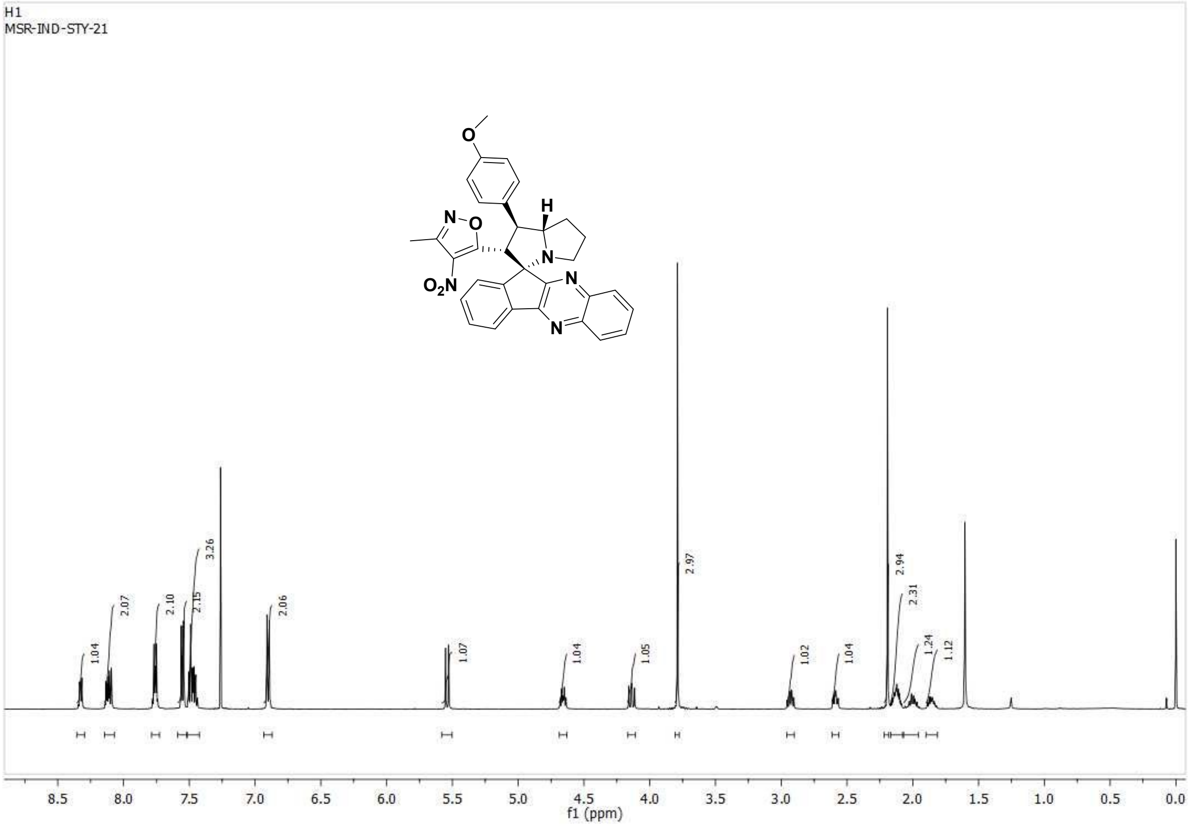

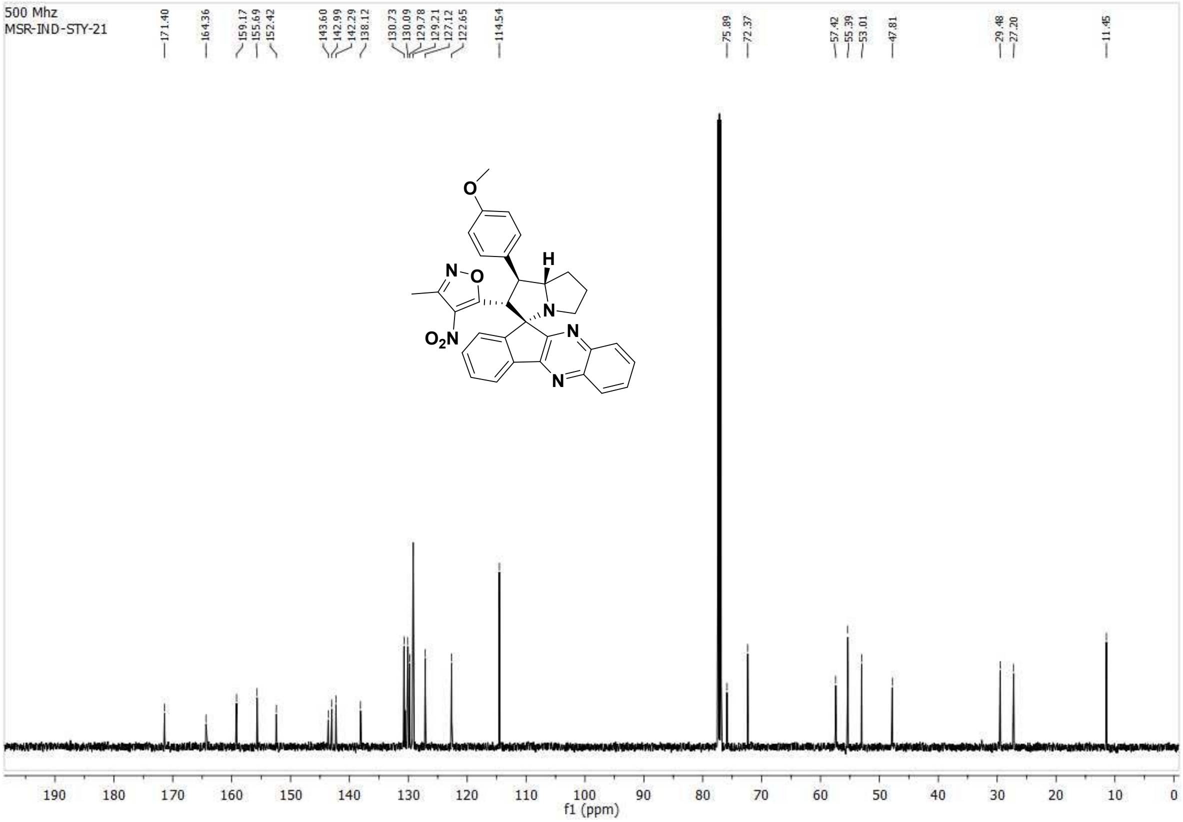

^{1}H & ^{13}C Spectra of 4k

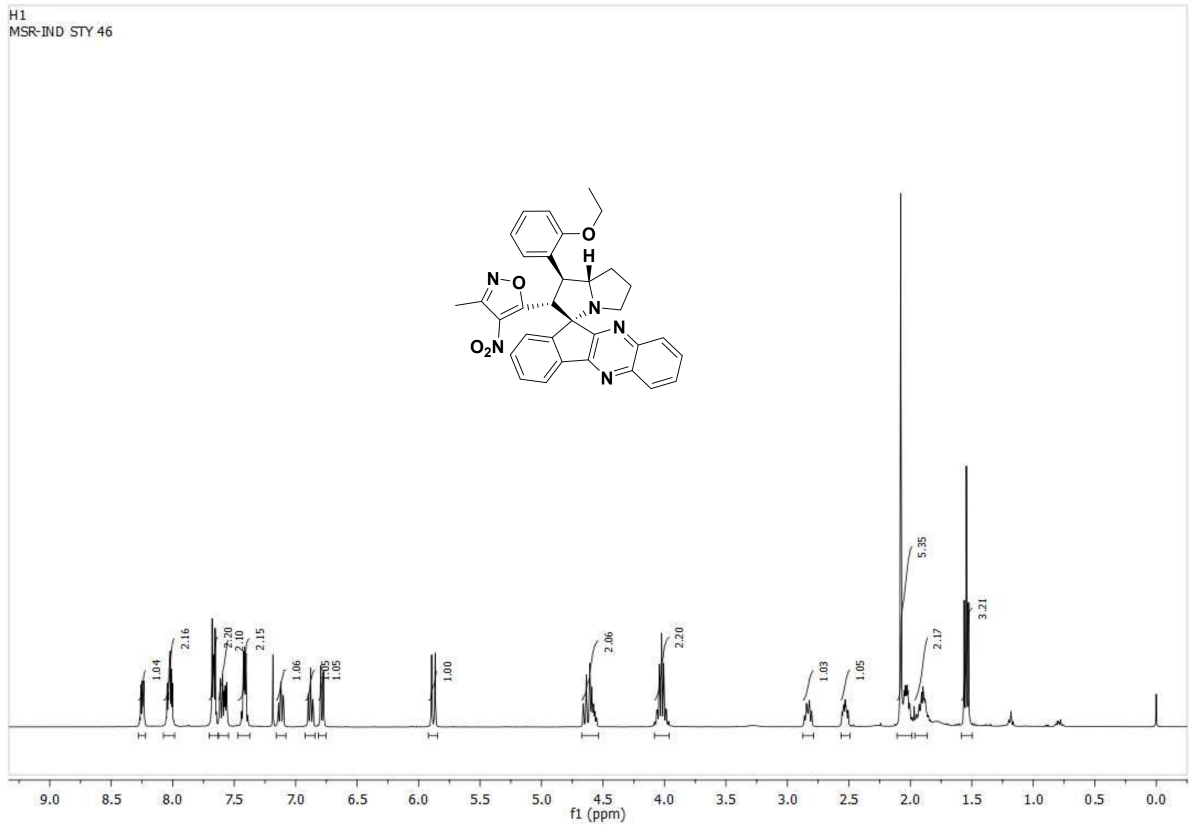

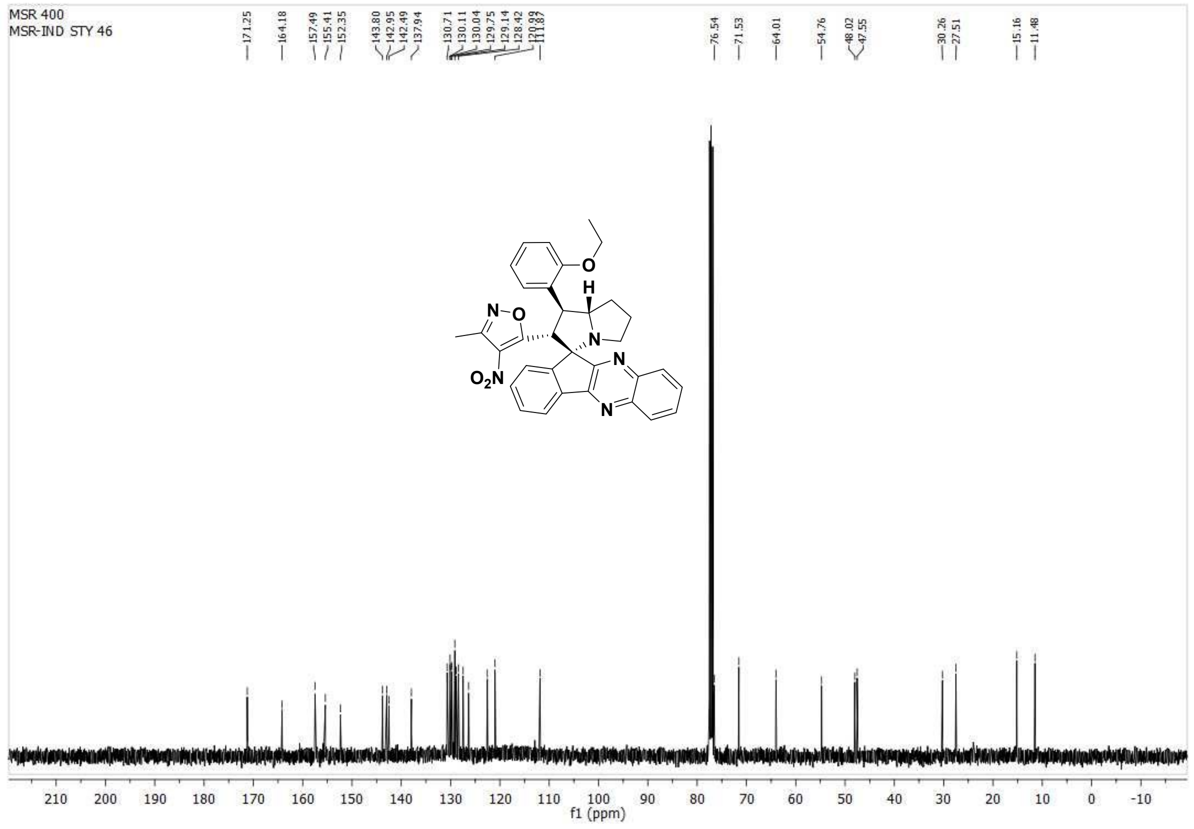

^{1}H & ^{13}C Spectra of 4l

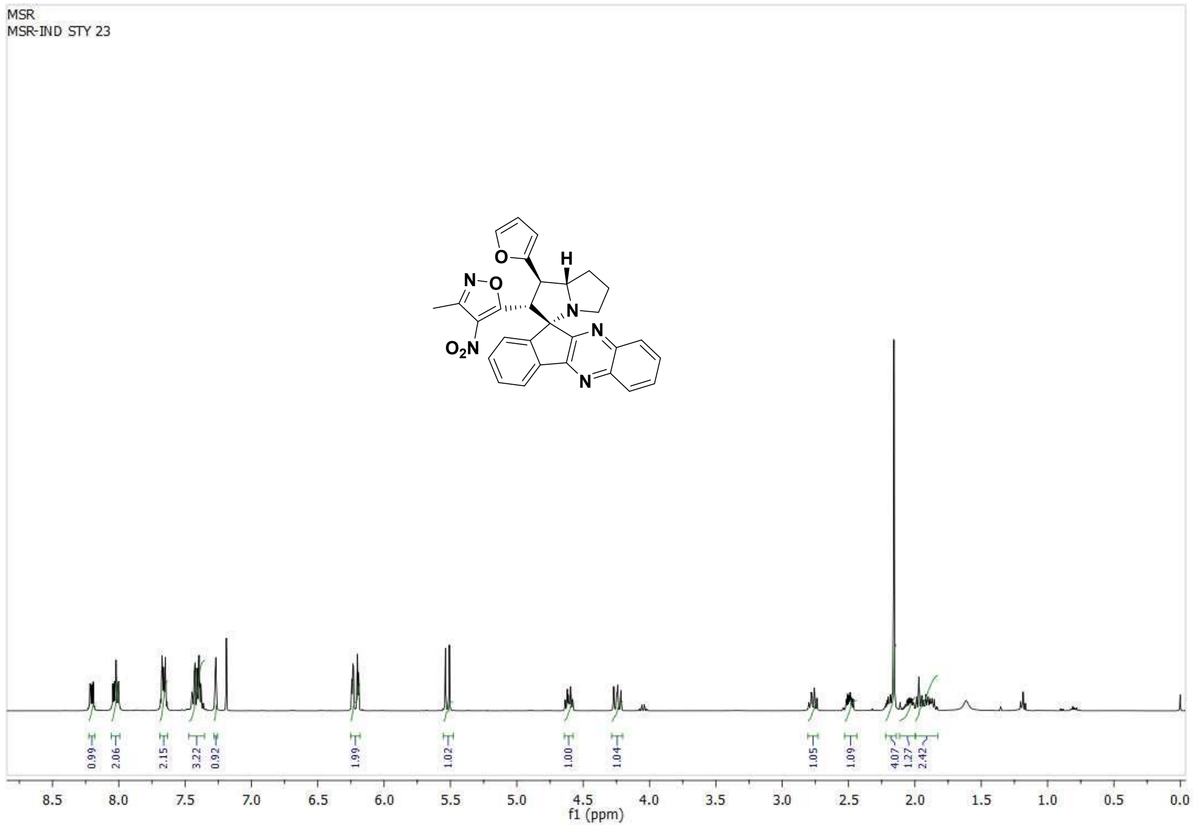

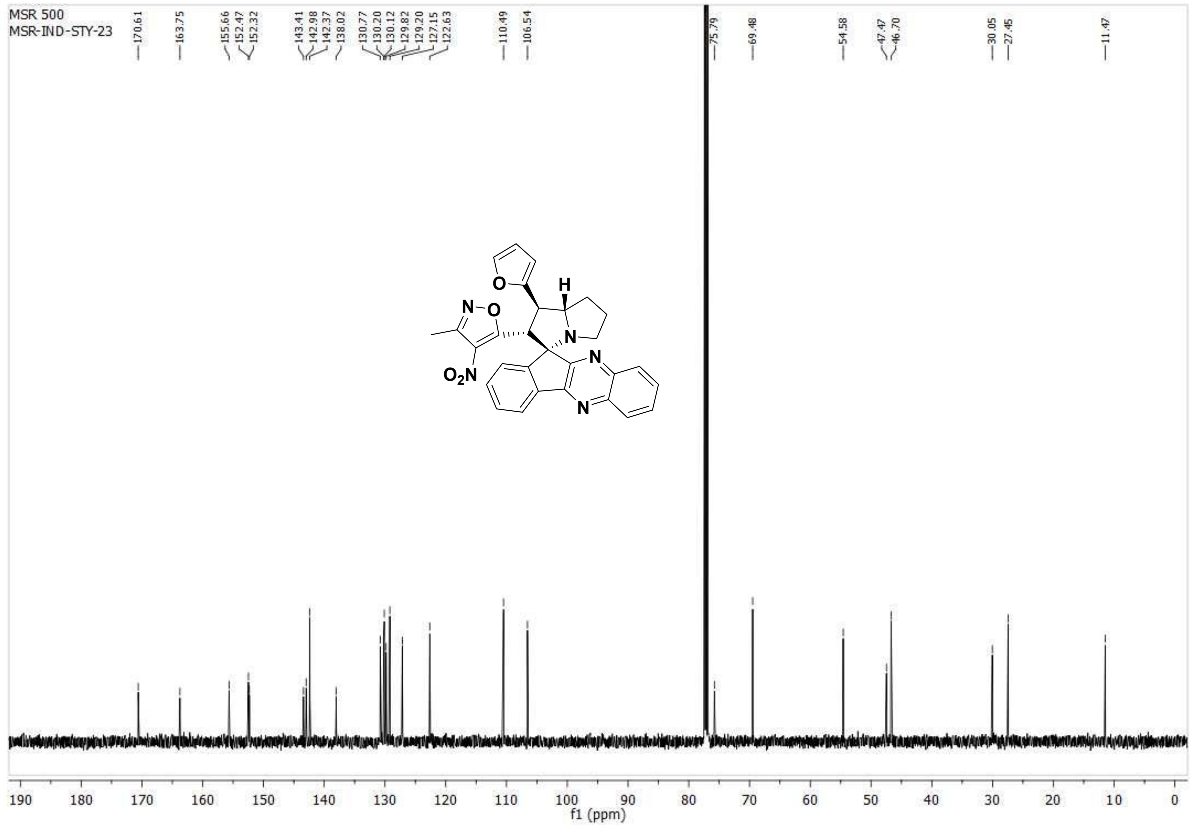

^{1}H & ^{13}C Spectra of 4m

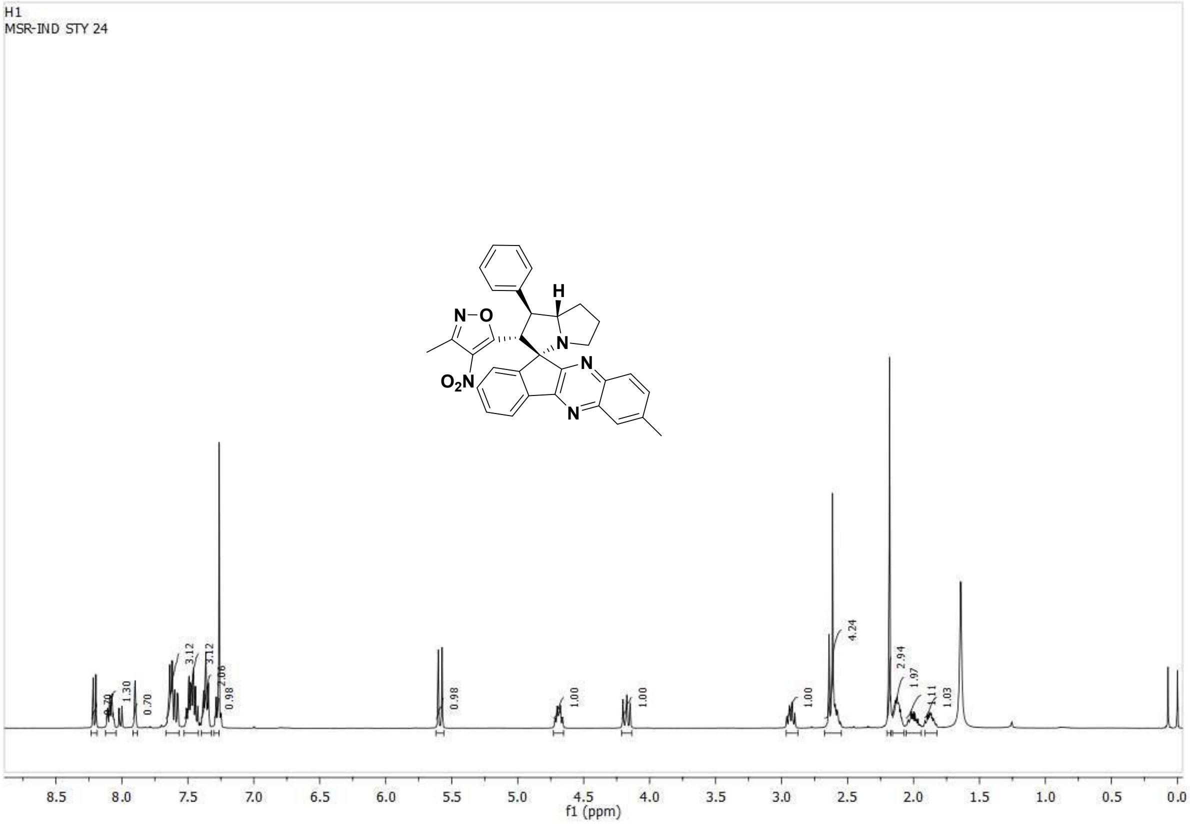

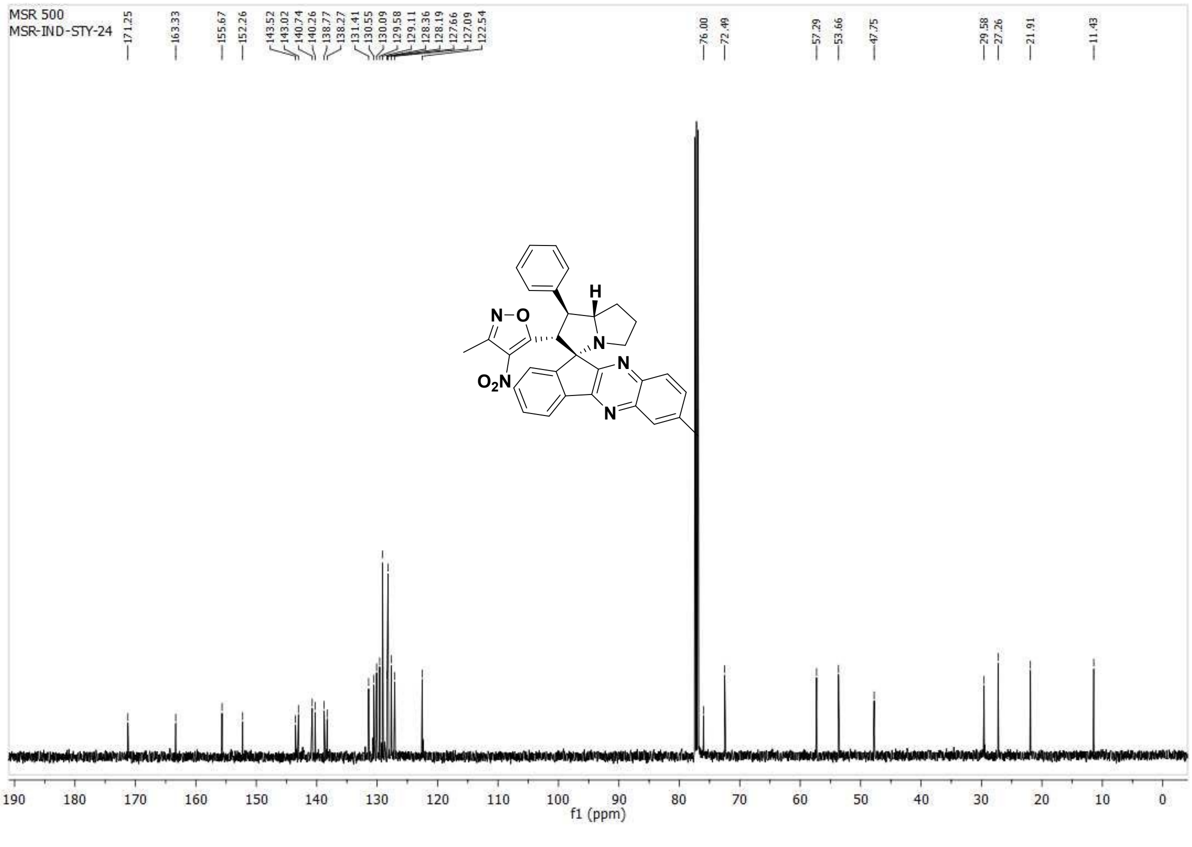

^{1}H &[13]C Spectra of 4n

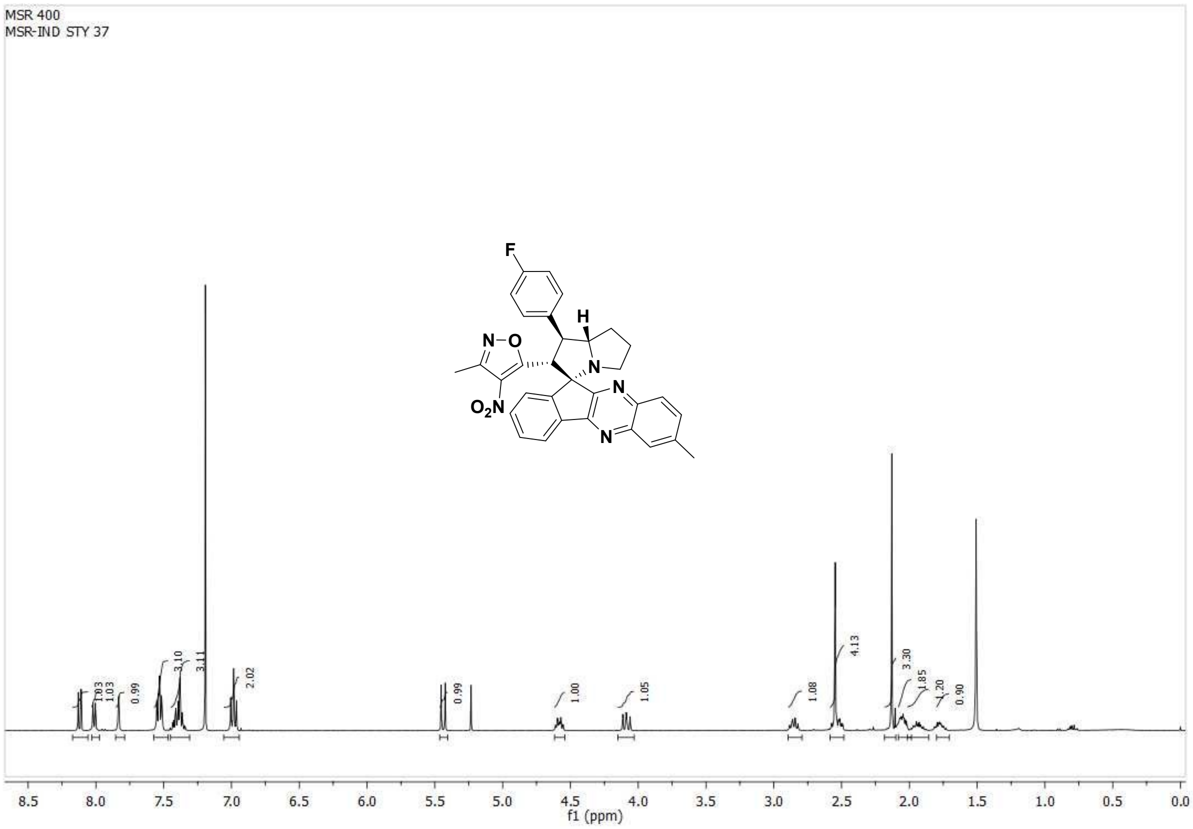

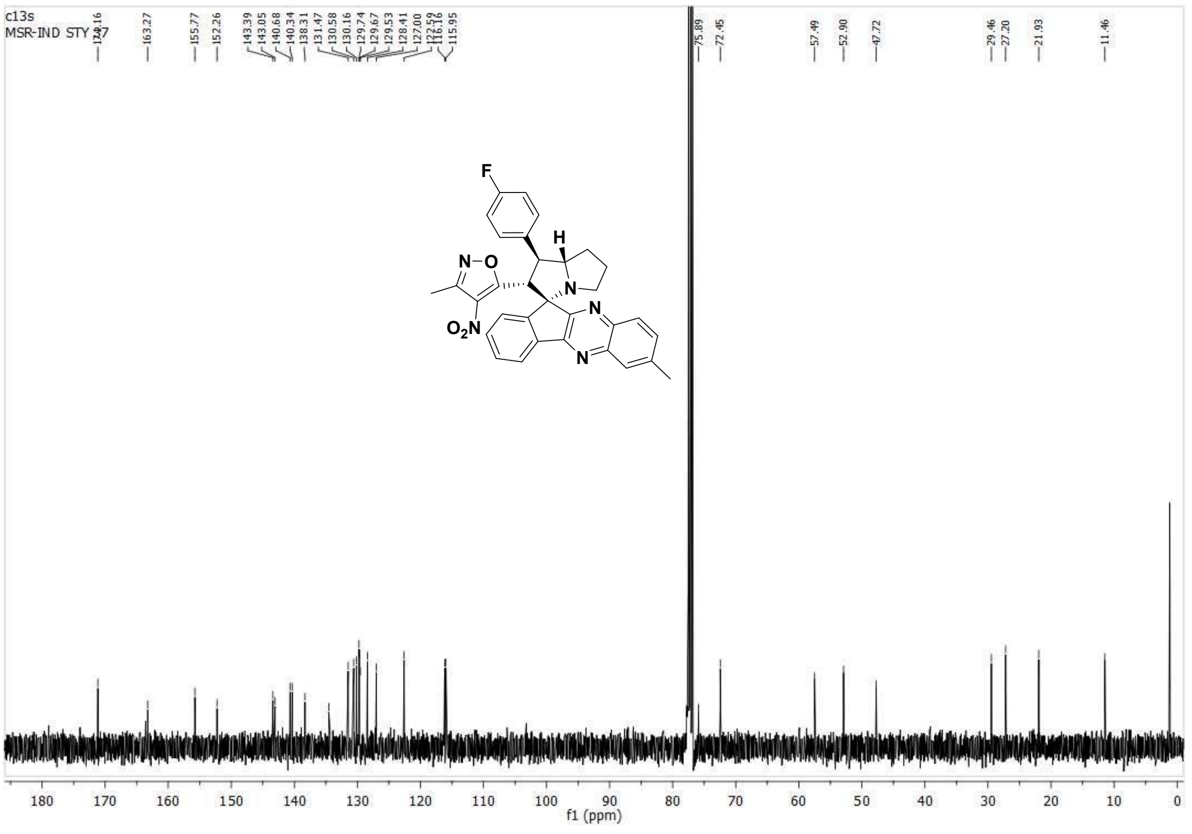

^{19}F Spectra of 4n

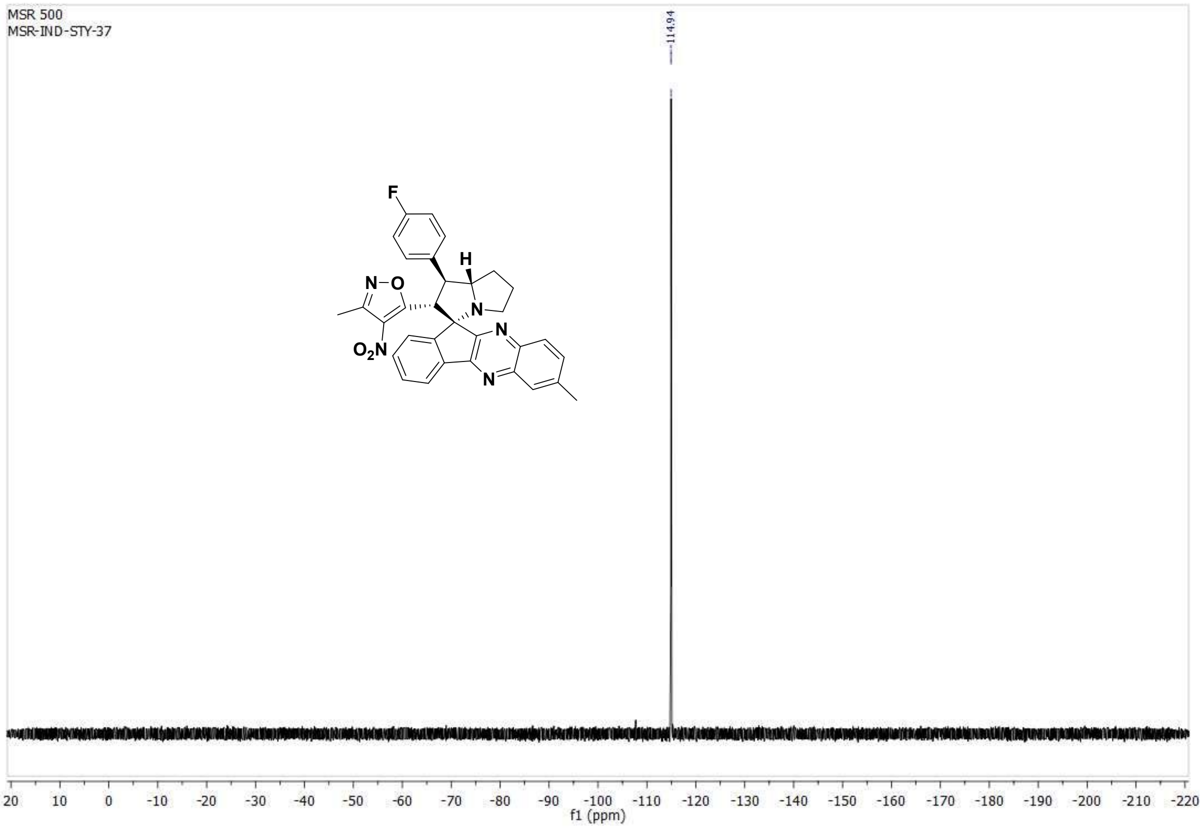

^{1}H & ^{13}C Spectra of 4o

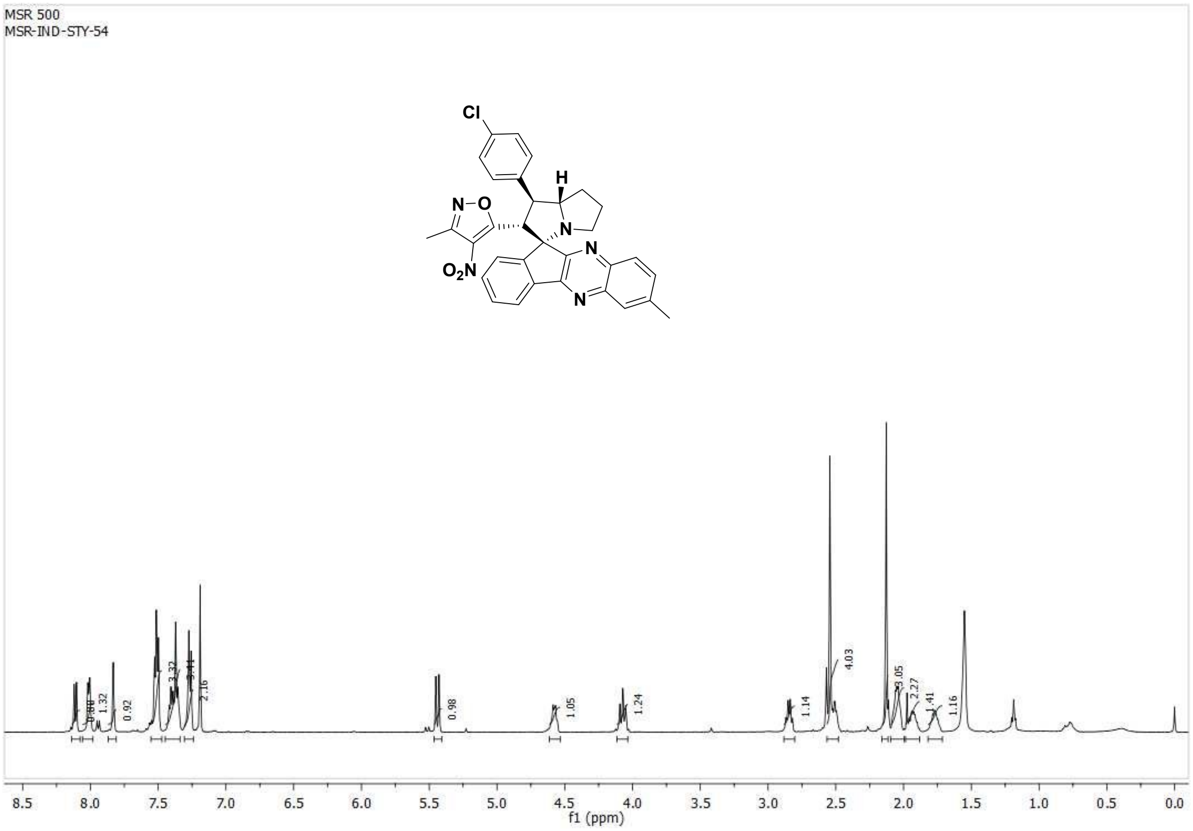

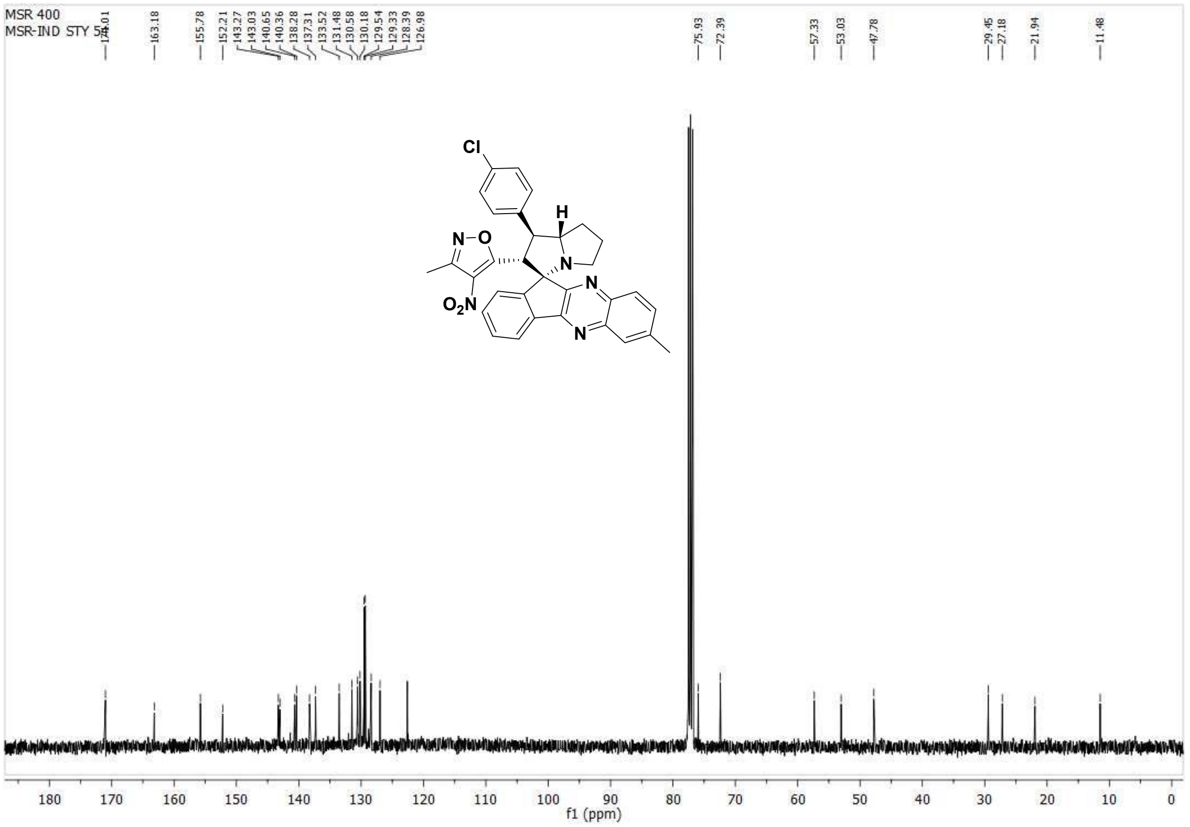

^{1}H & ^{13}C Spectra of 4p

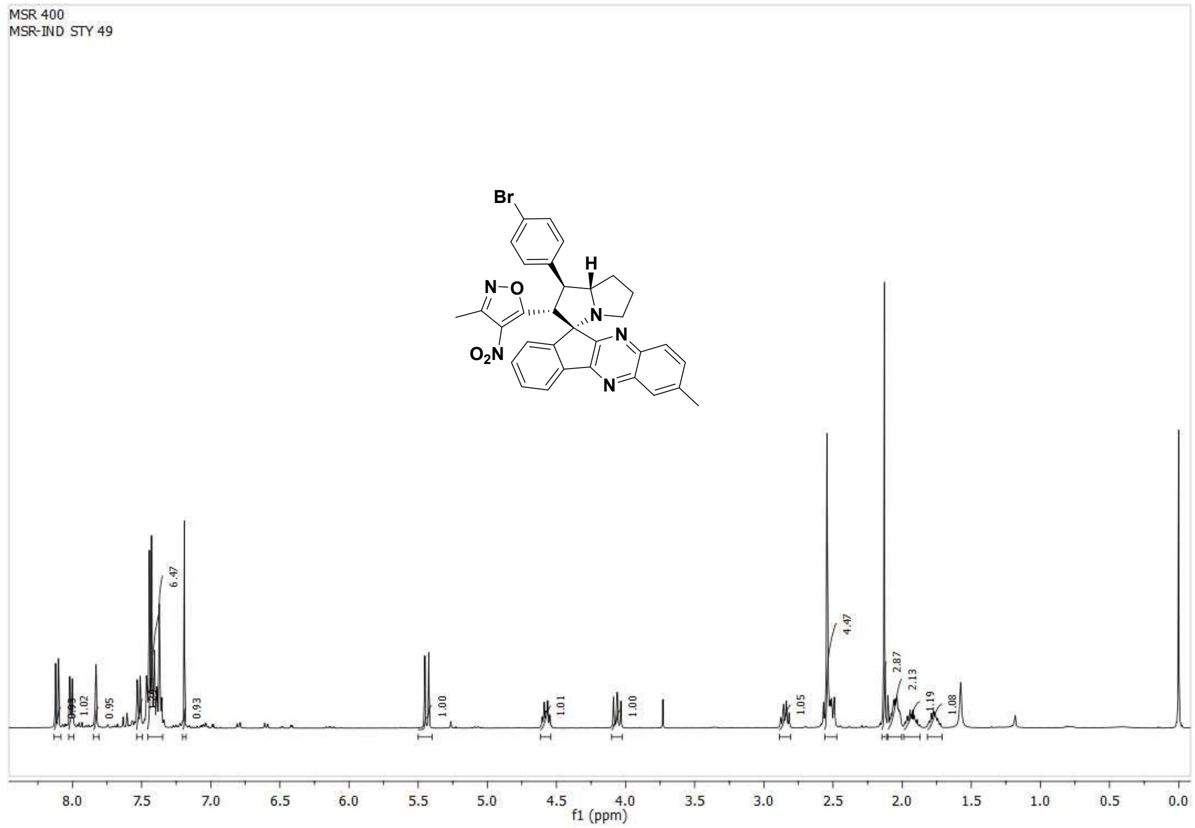

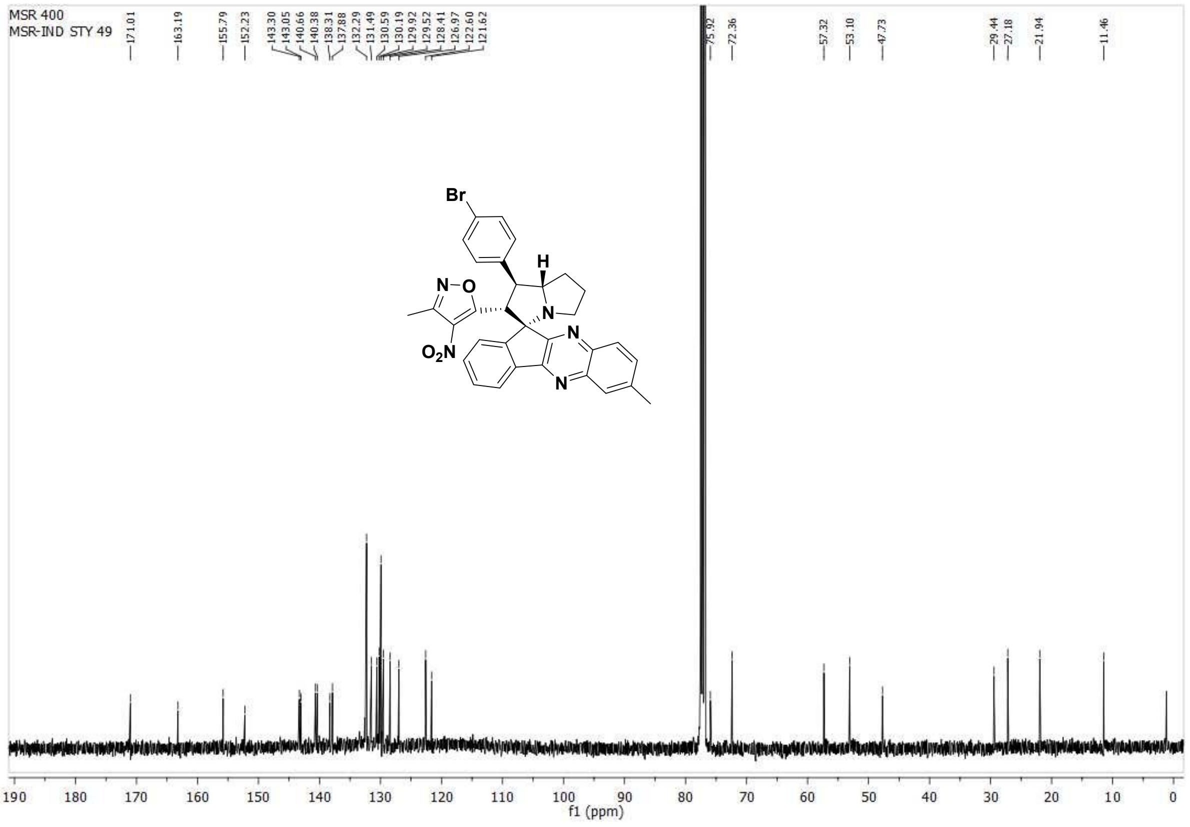

^{1}H &^{13}C Spectra of 4q

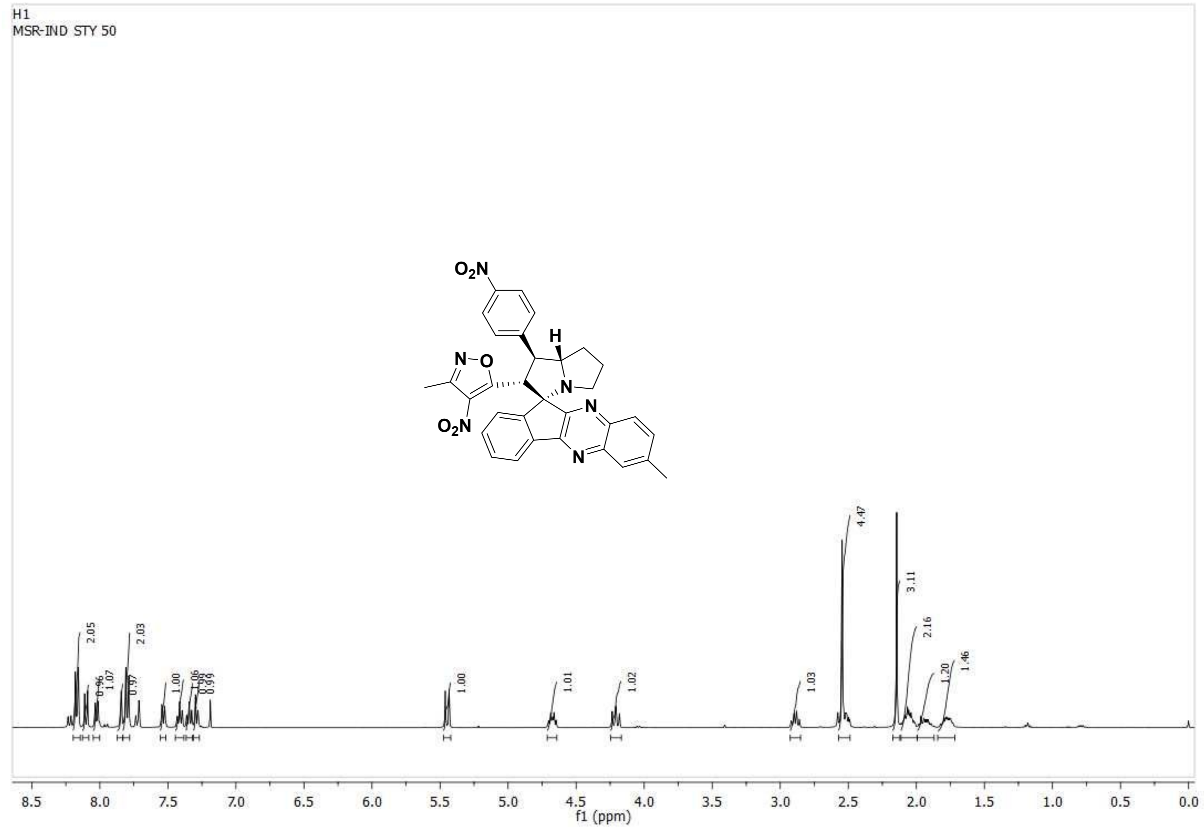

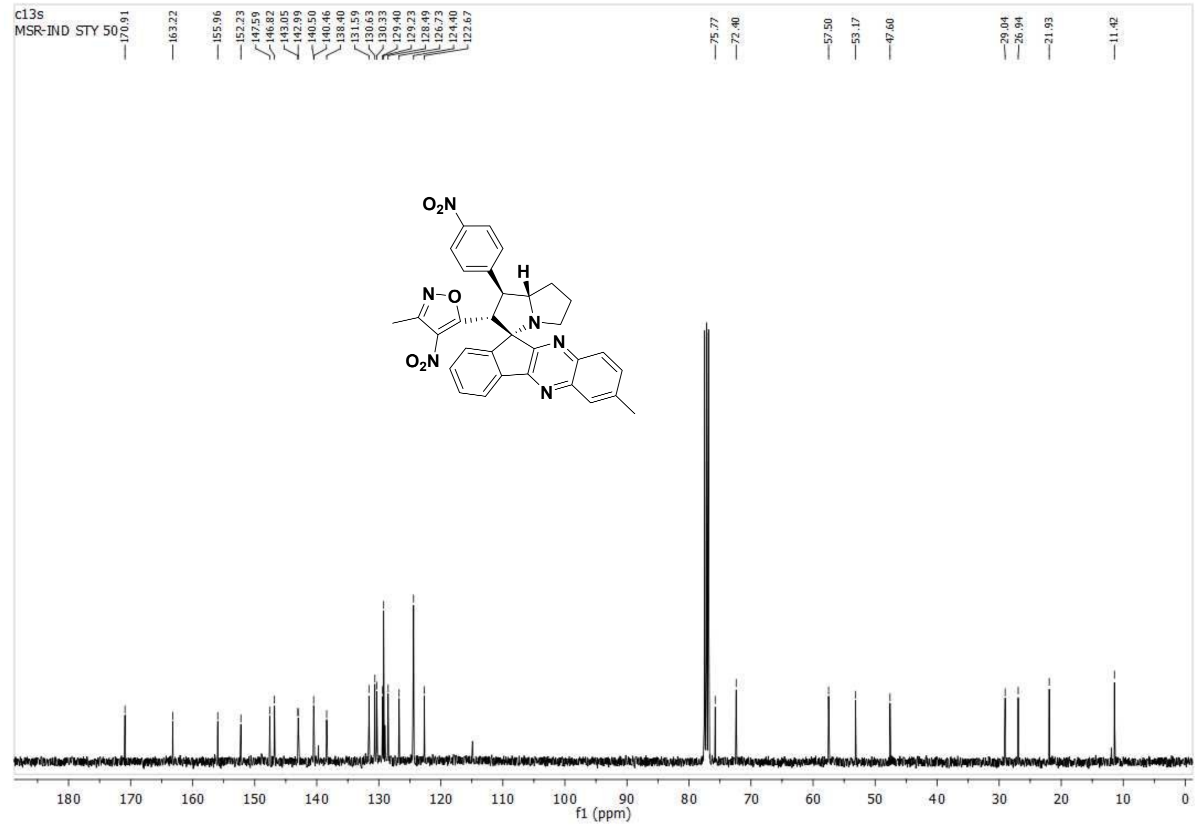

^{1}H &[13]C Spectra of 4r

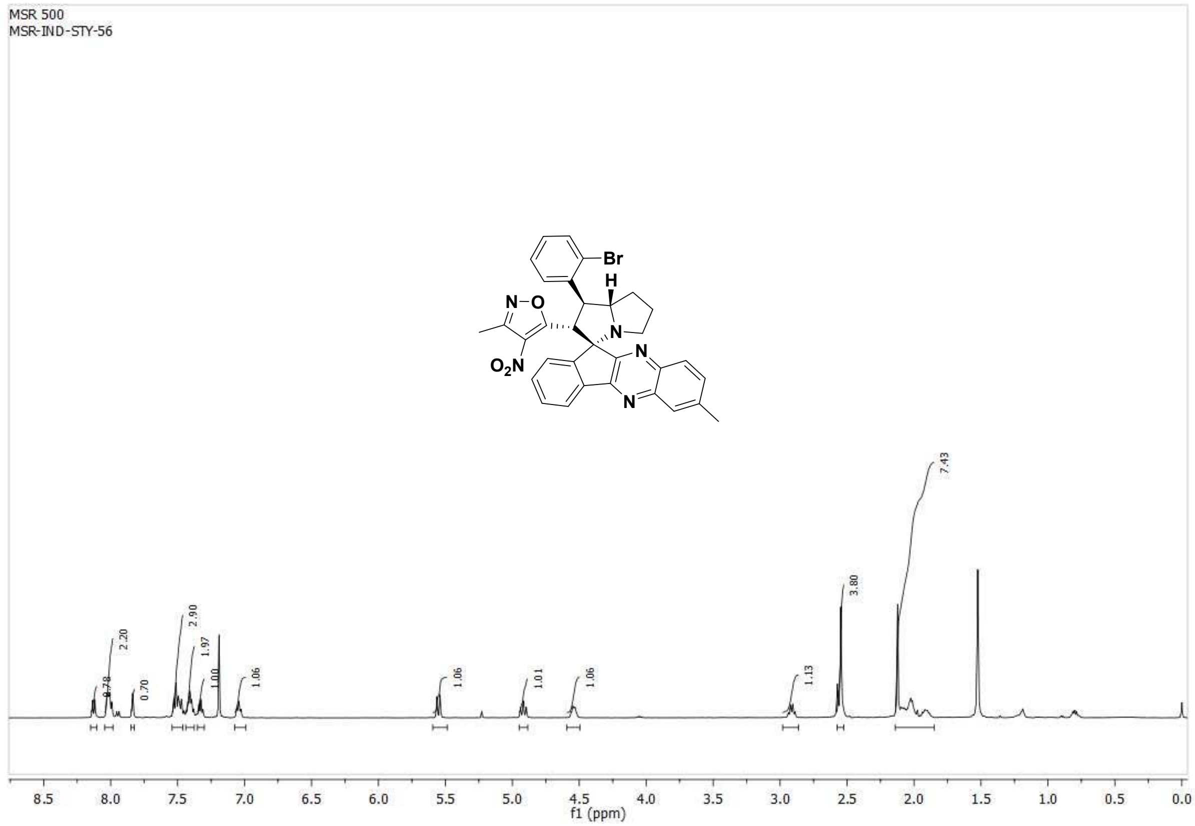

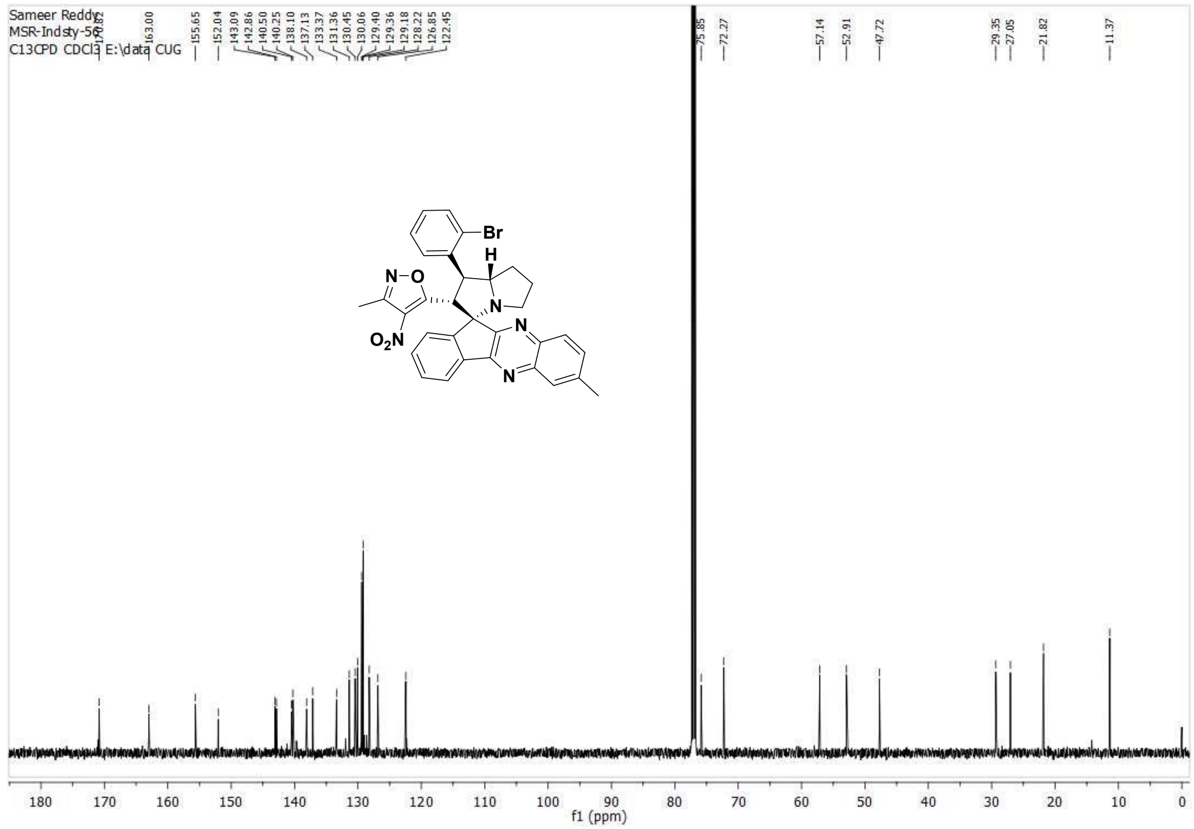

^{1}H & ^{13}C Spectra of 4s

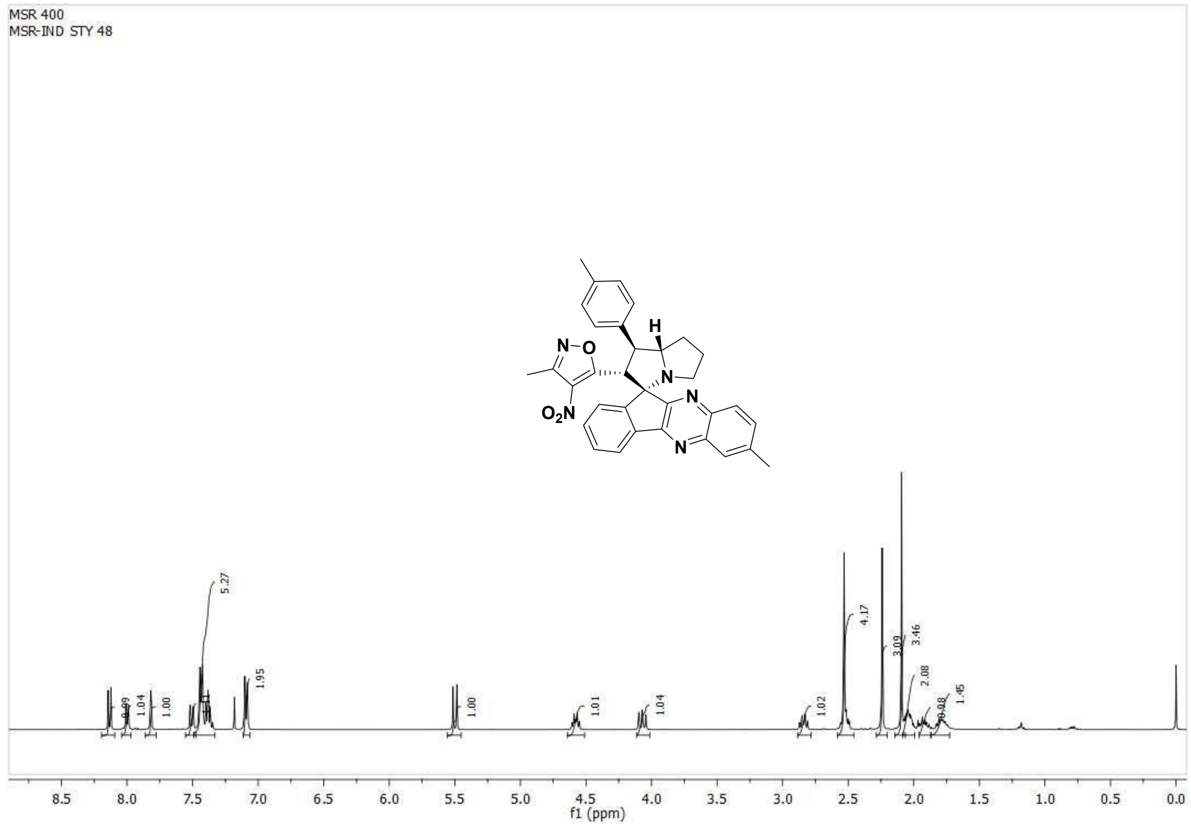

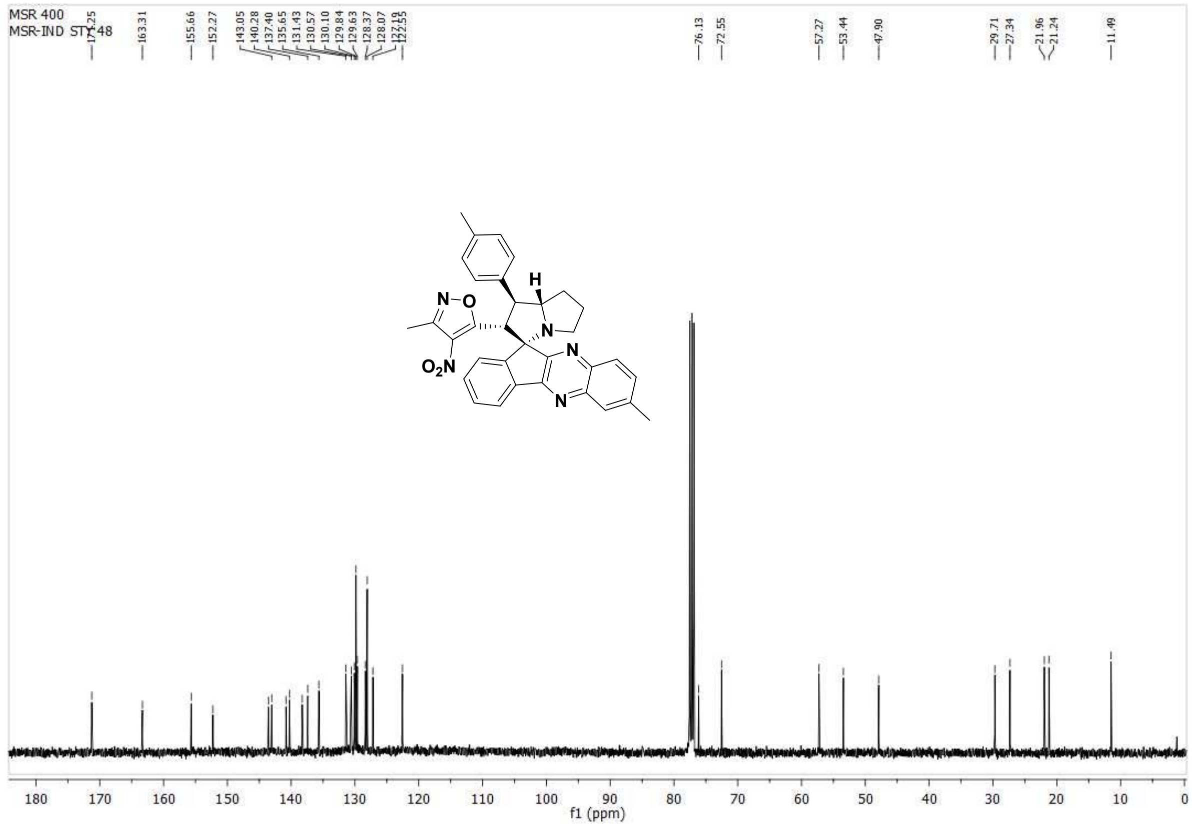

^{1}H & ^{13}C Spectra of 4t

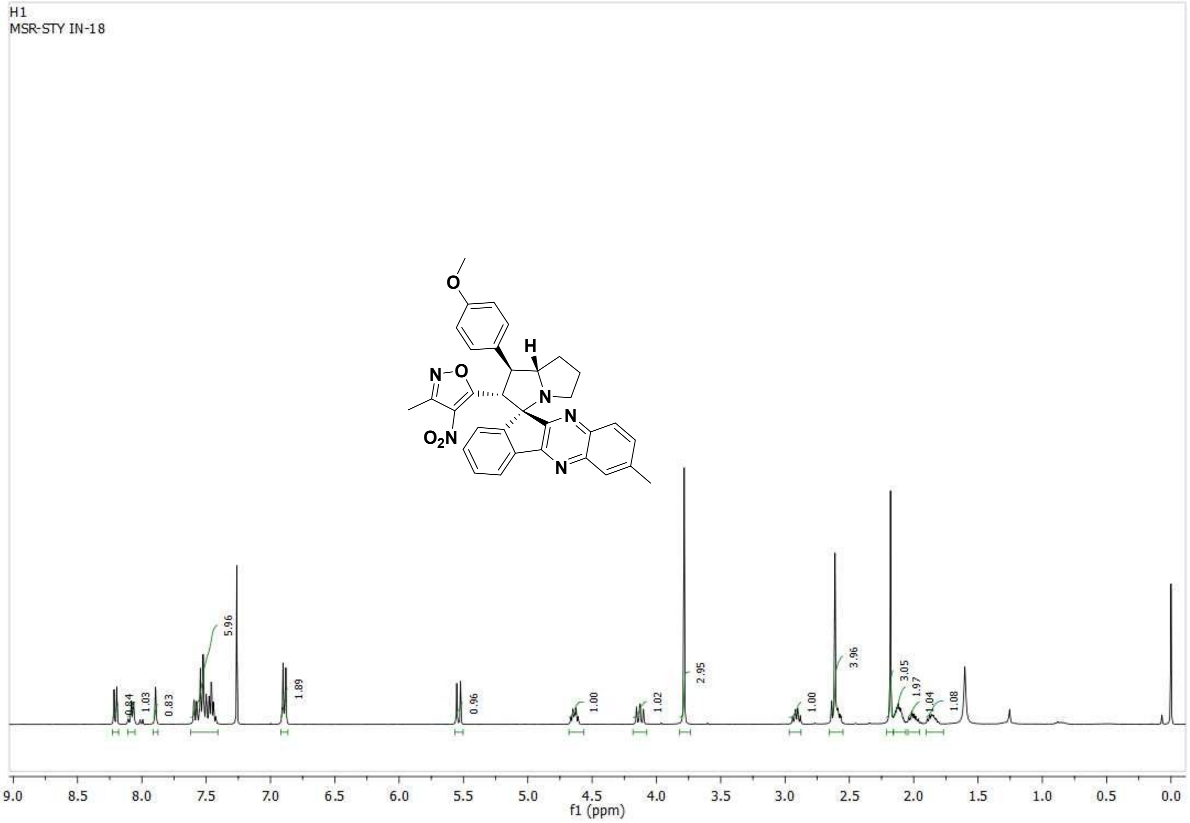

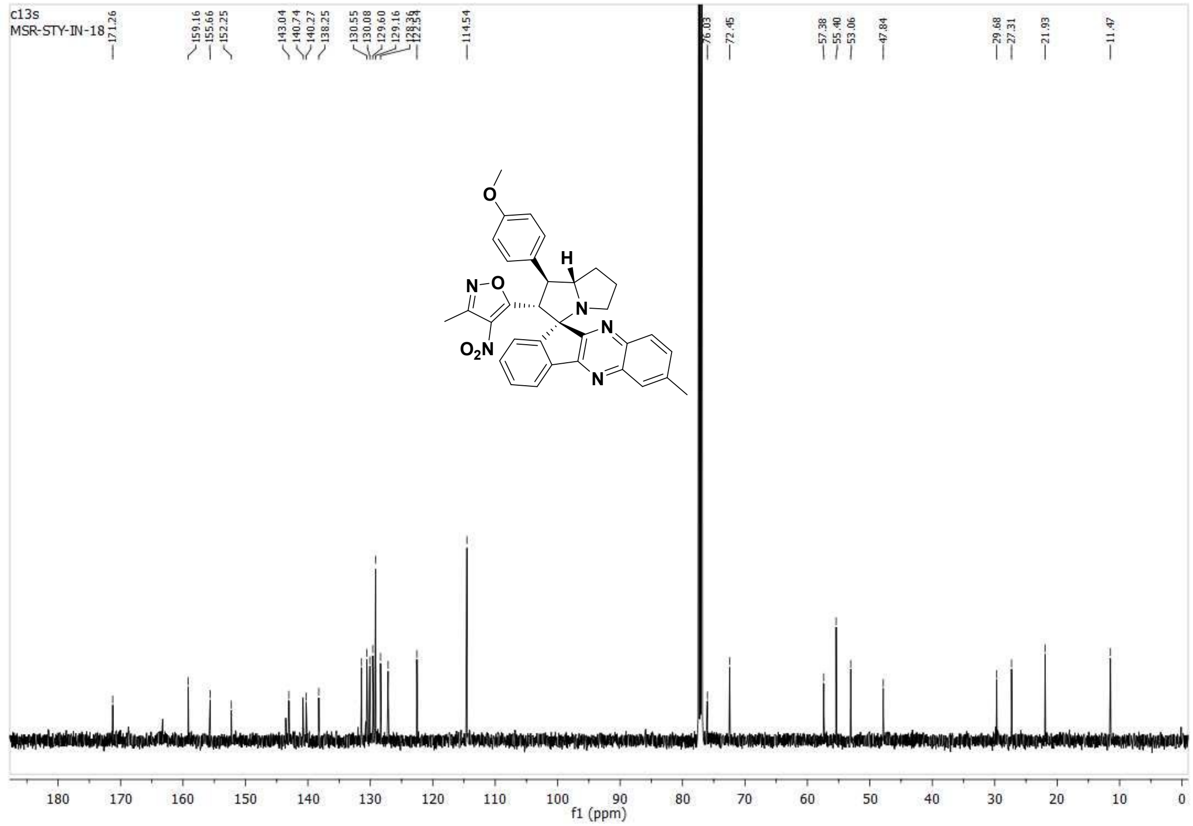

^{1}H &[13]C Spectra of 4u

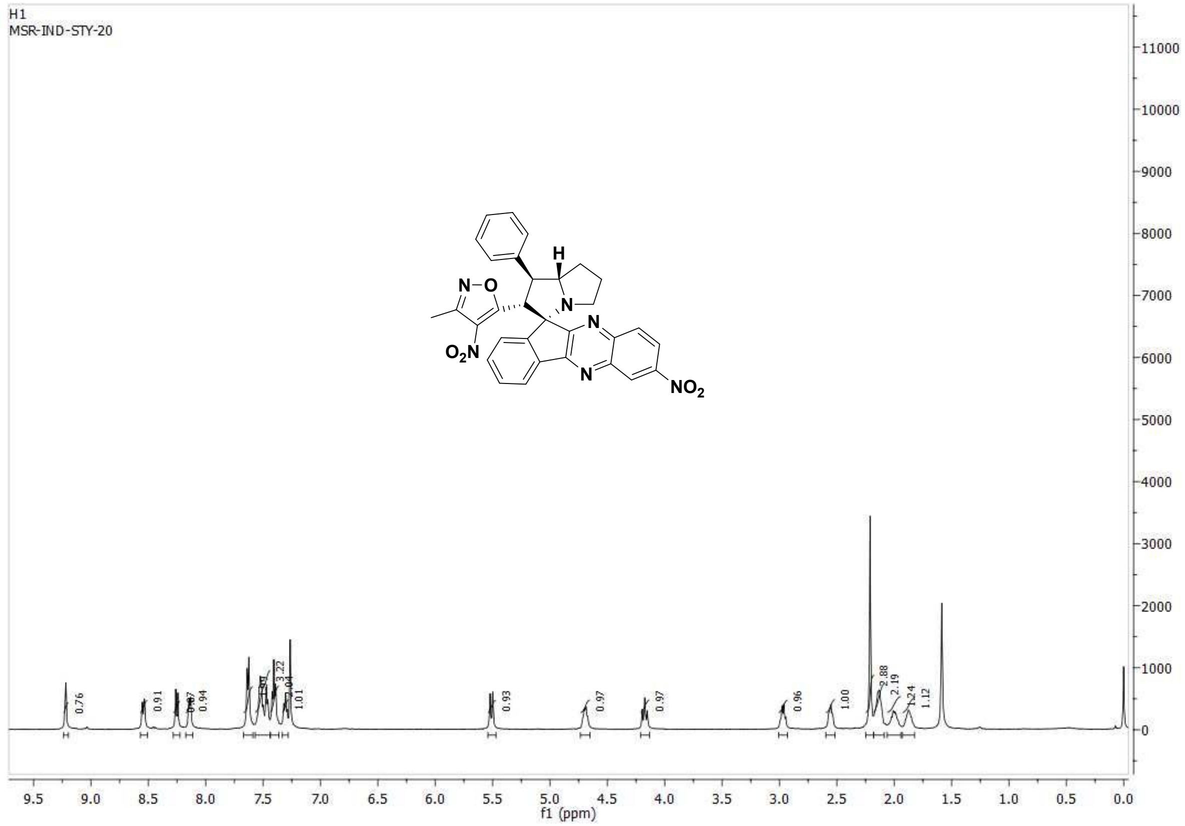

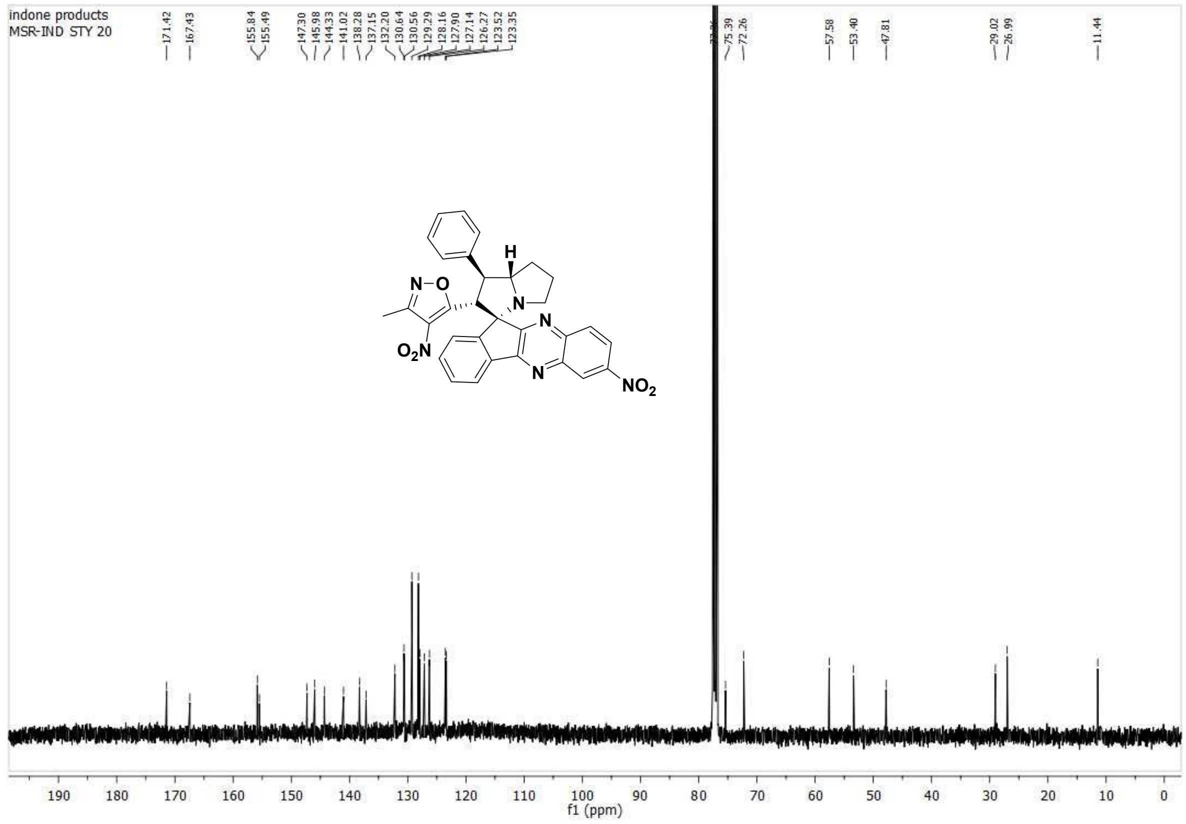

^{1}H &[13]C Spectra of 4v

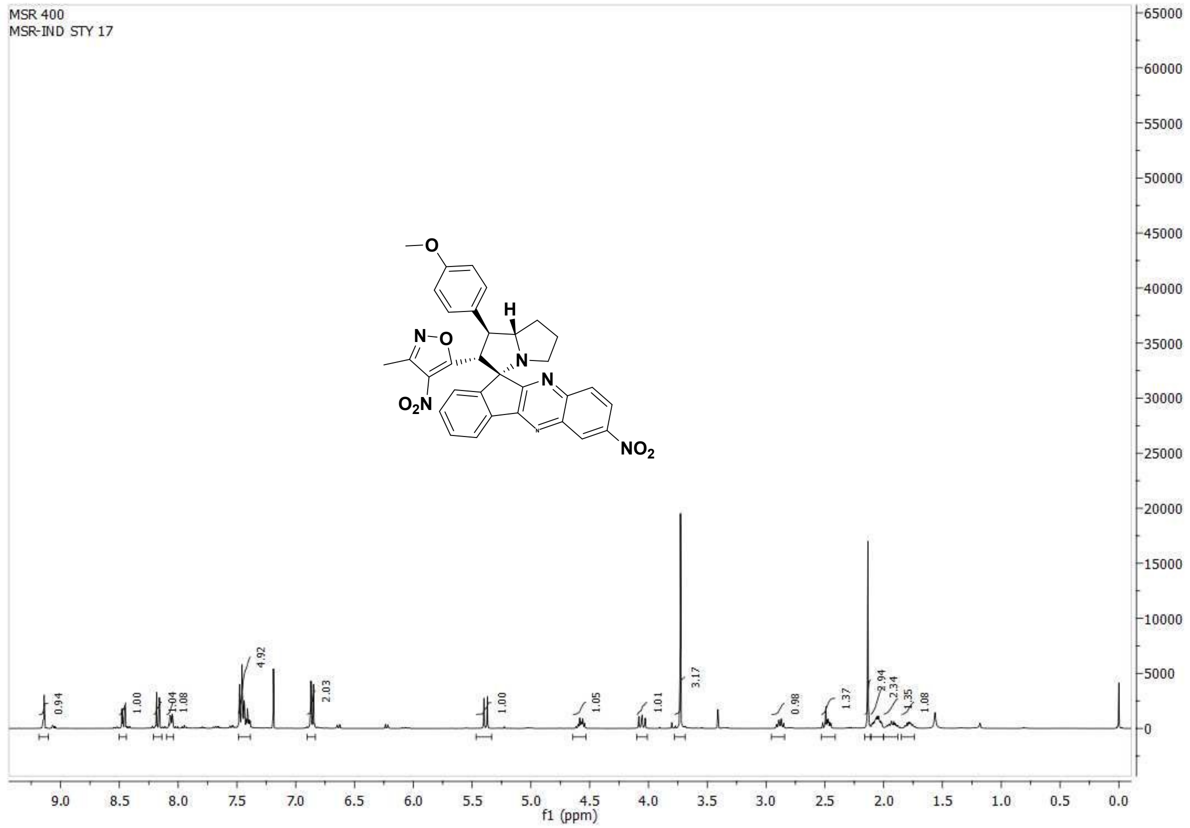

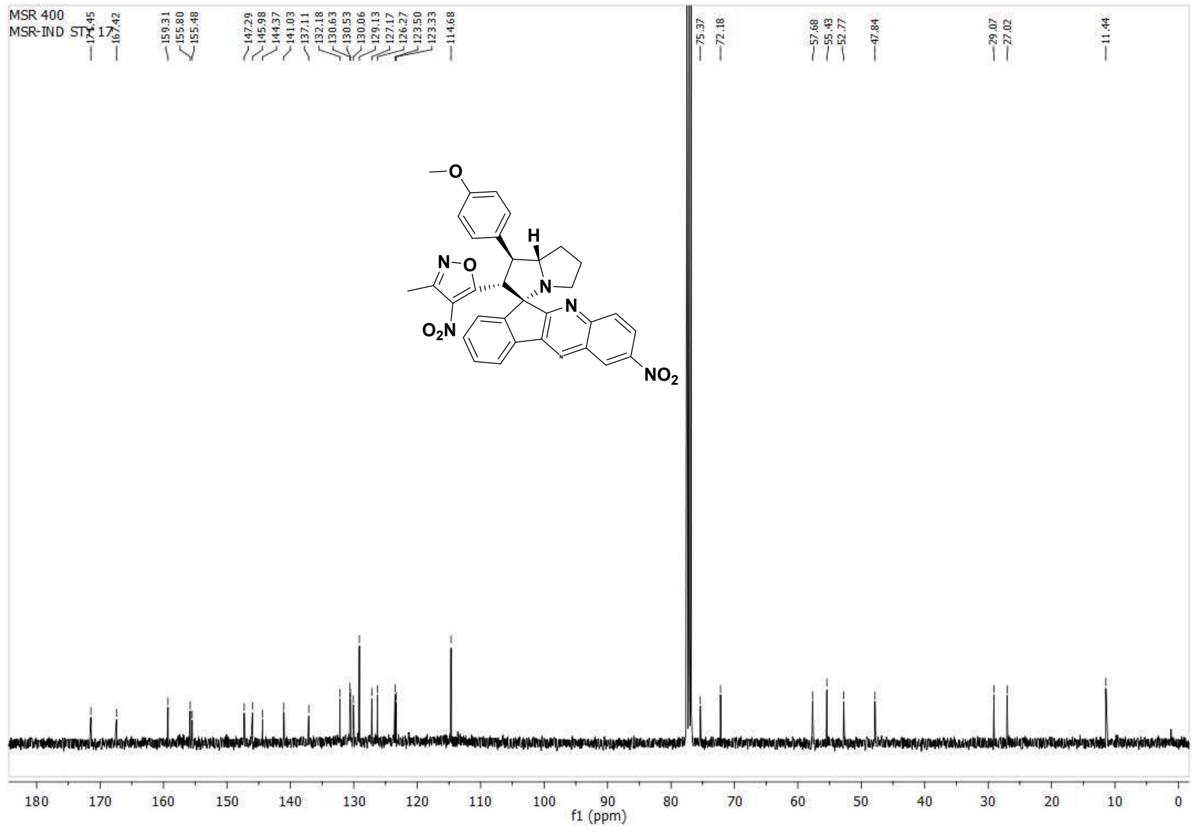

^{1}H &[13]C Spectra of 6a

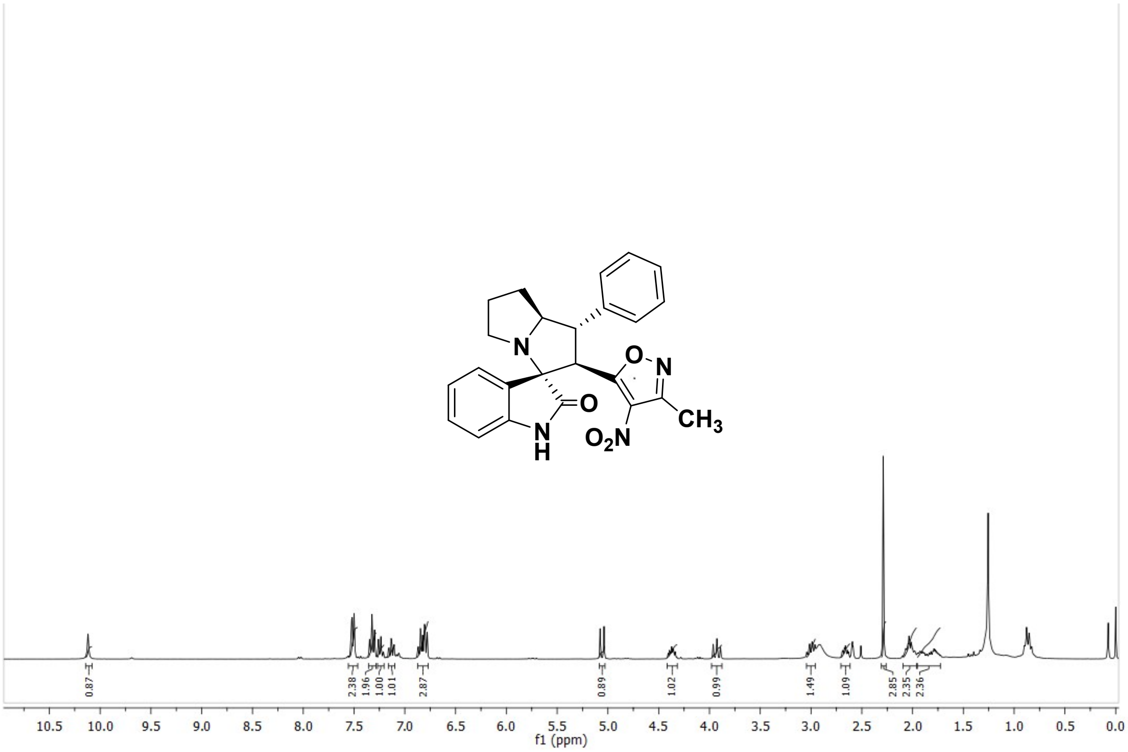

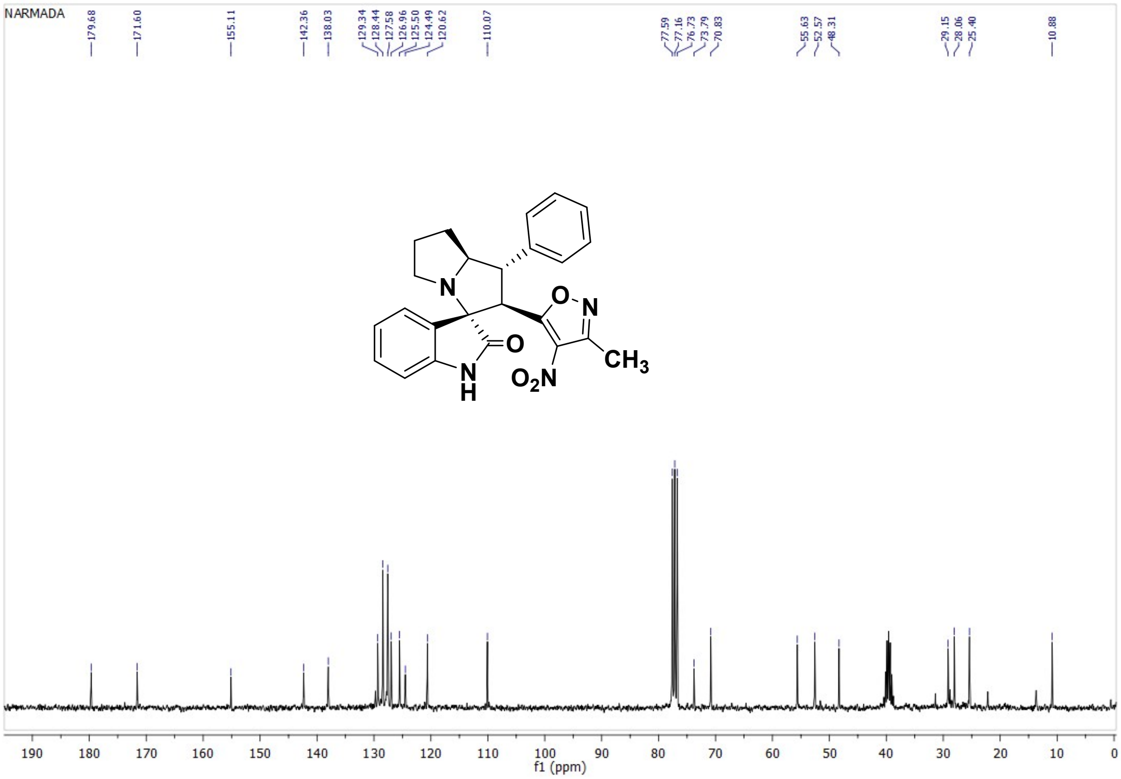

^{1}H &[13]C Spectra of 6b

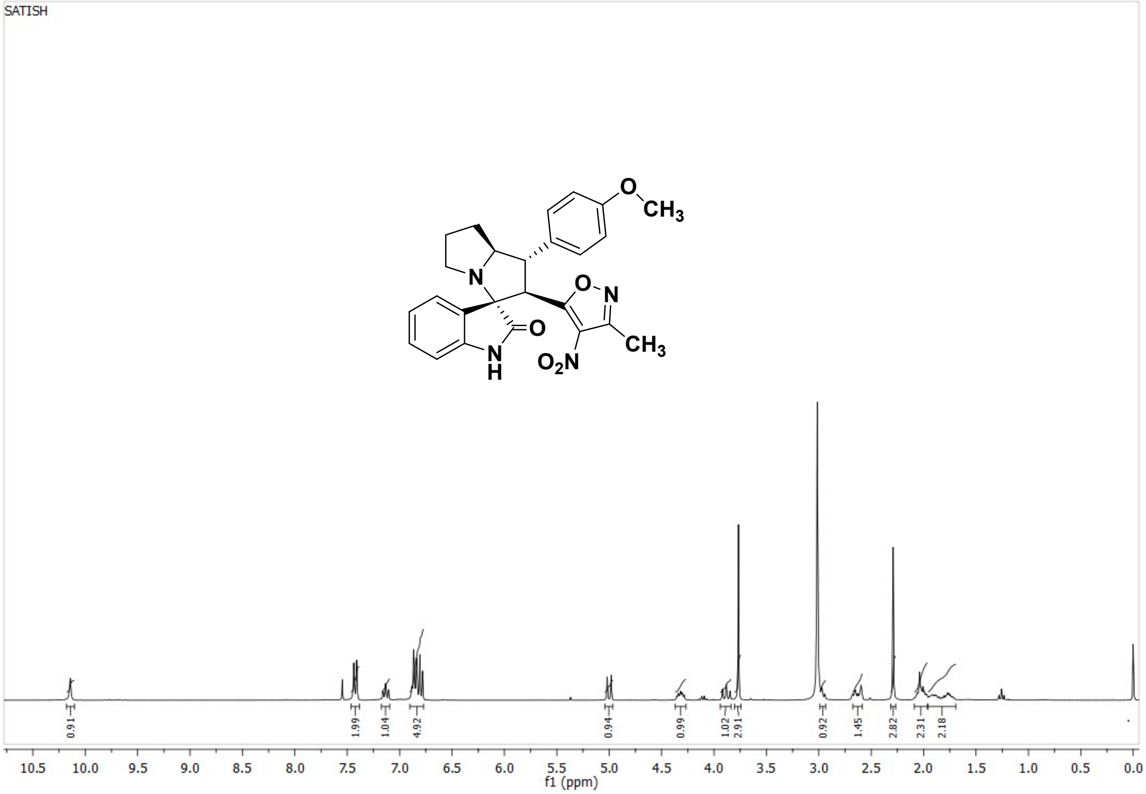

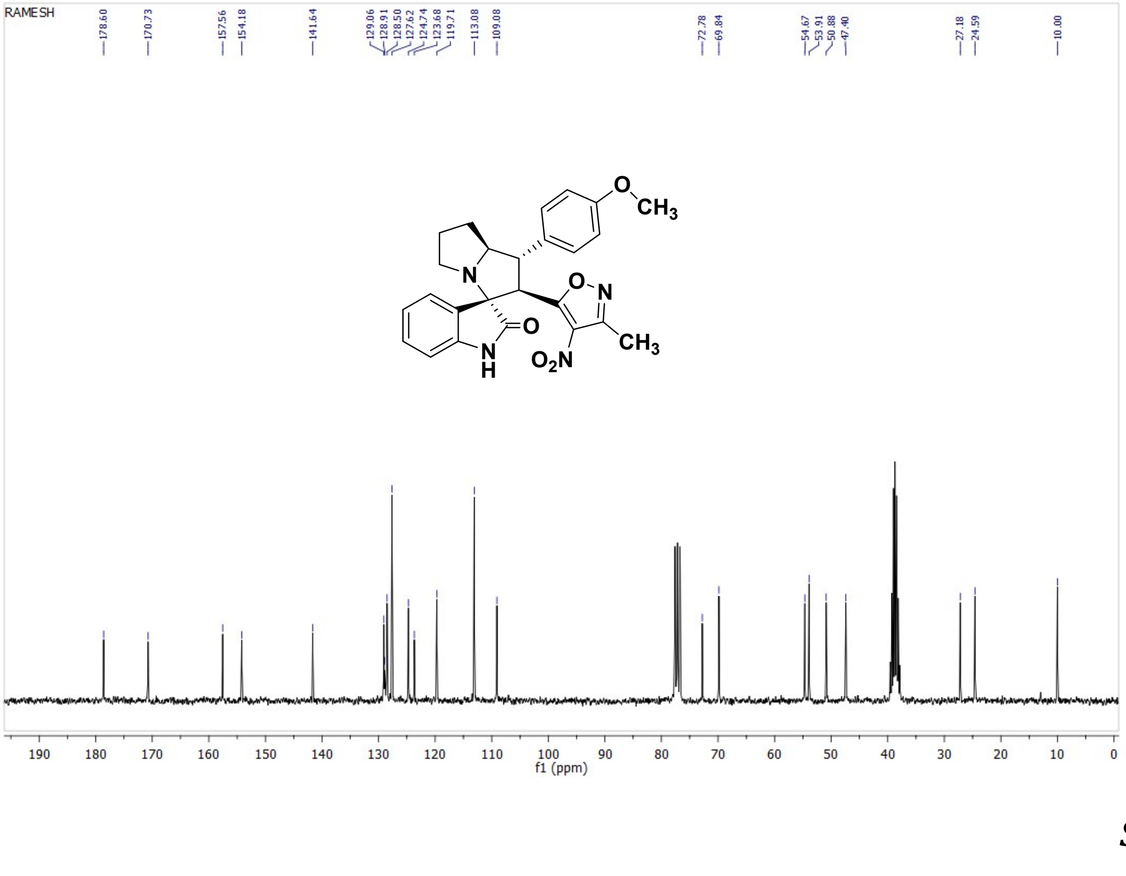

^{1}H &[13]C Spectra of 6c

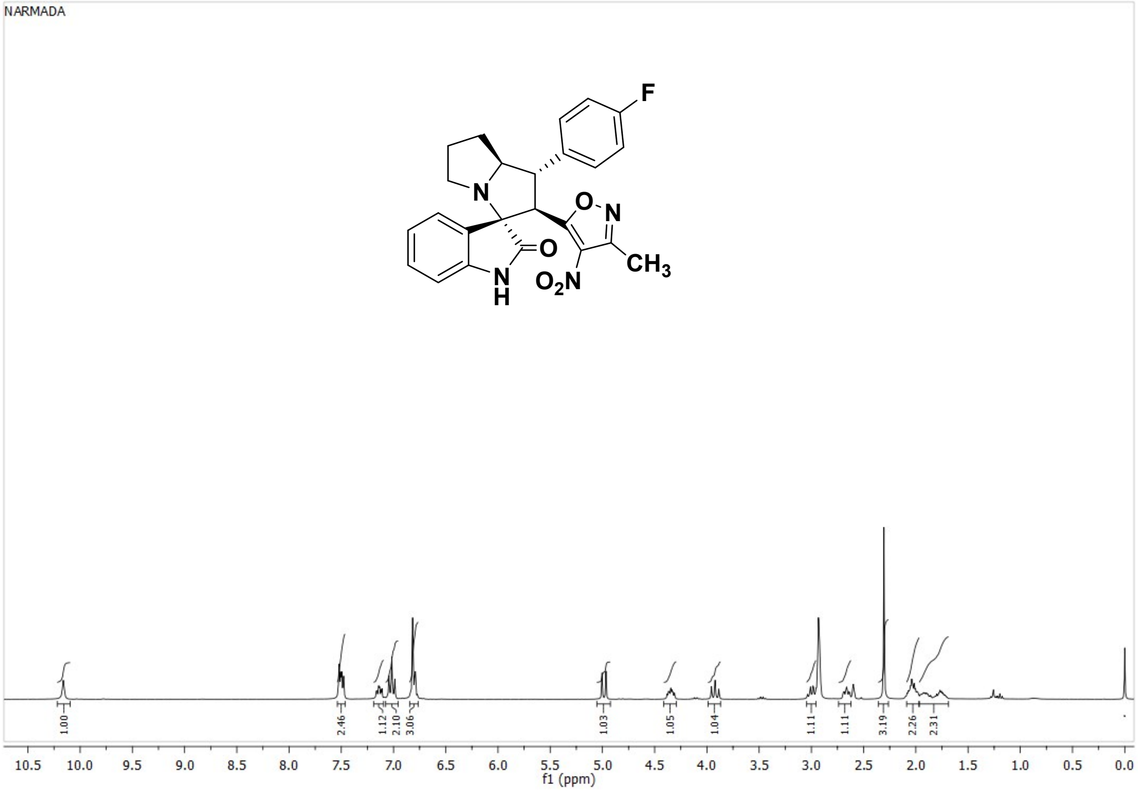

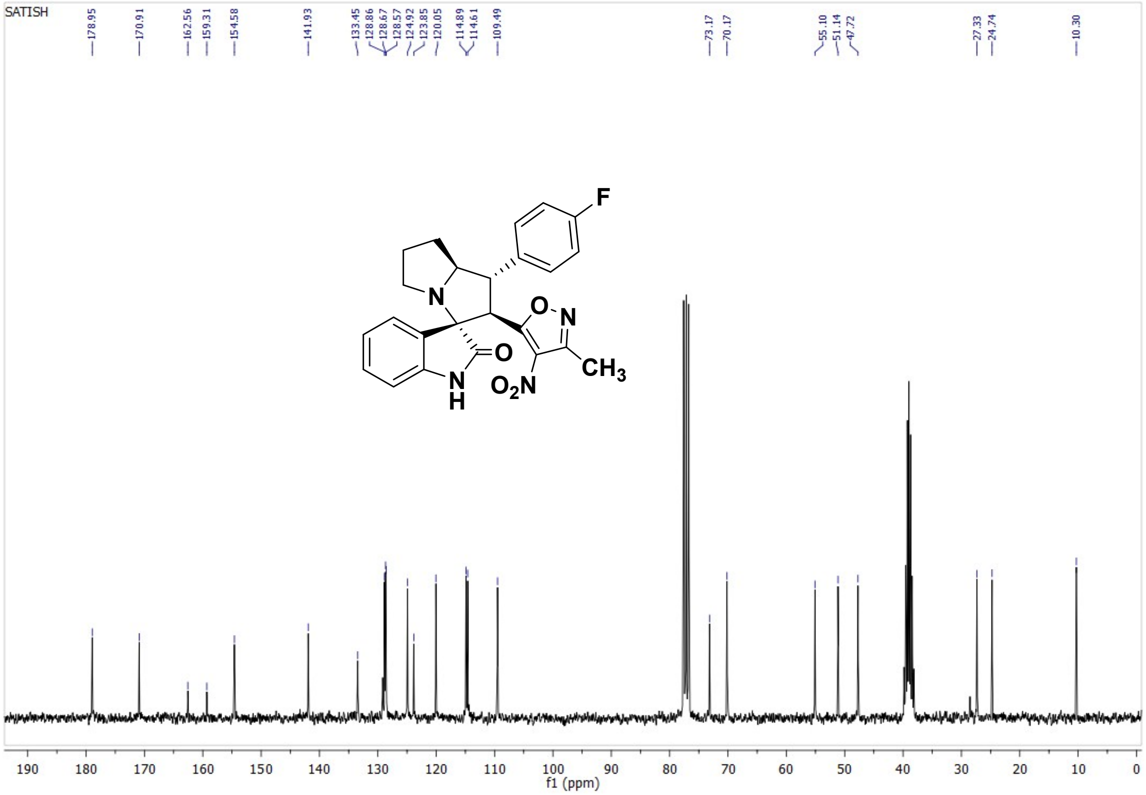

^{1}H &[13]C Spectra of 6d

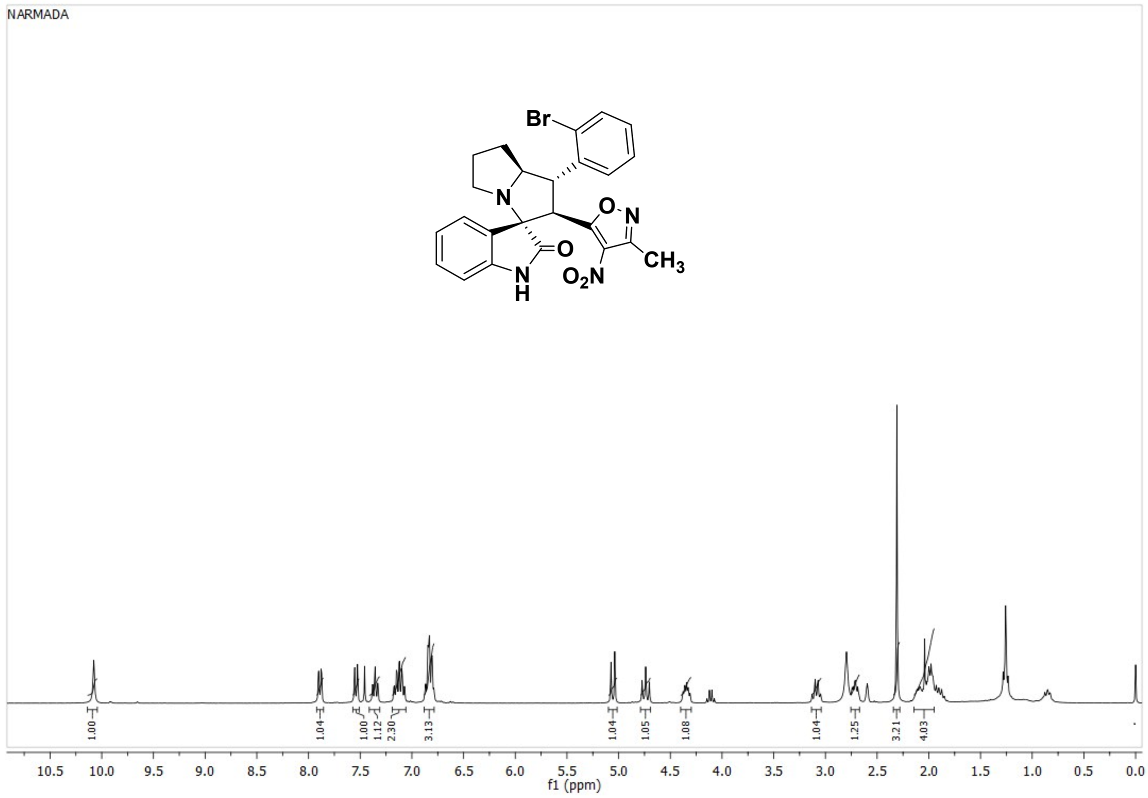

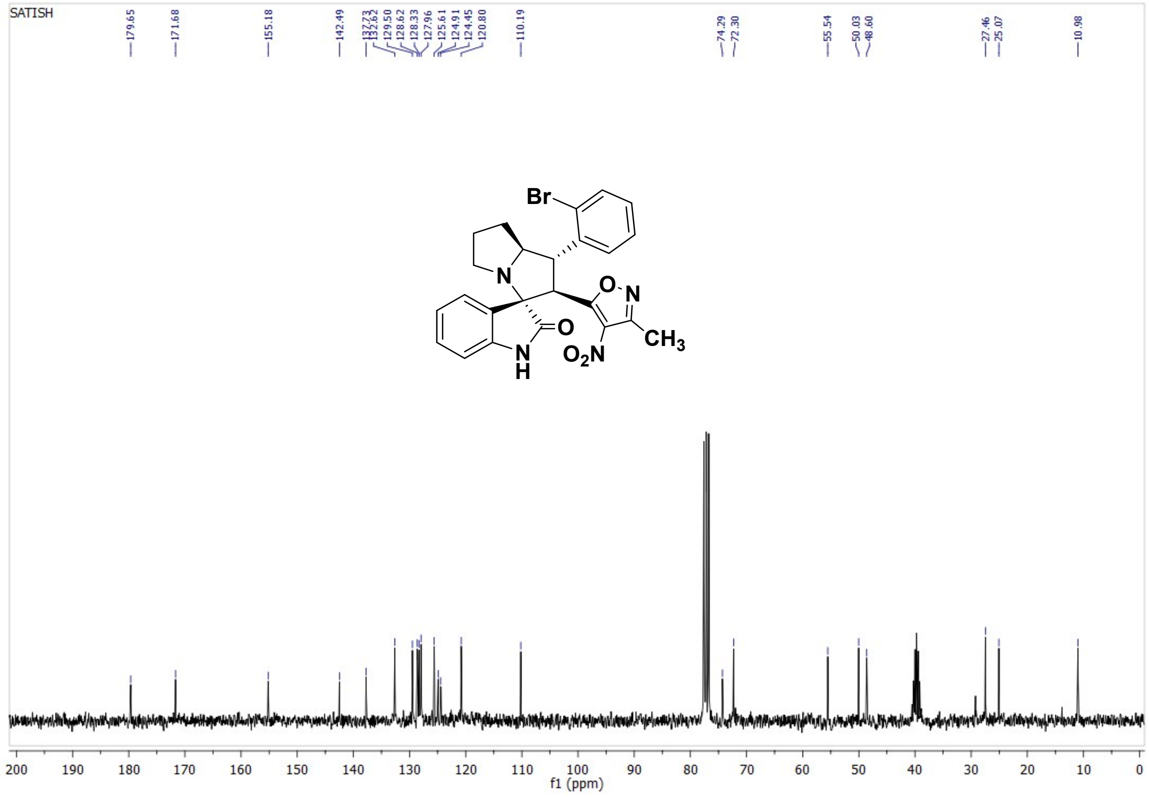

^{1}H &[13]C Spectra of 6e

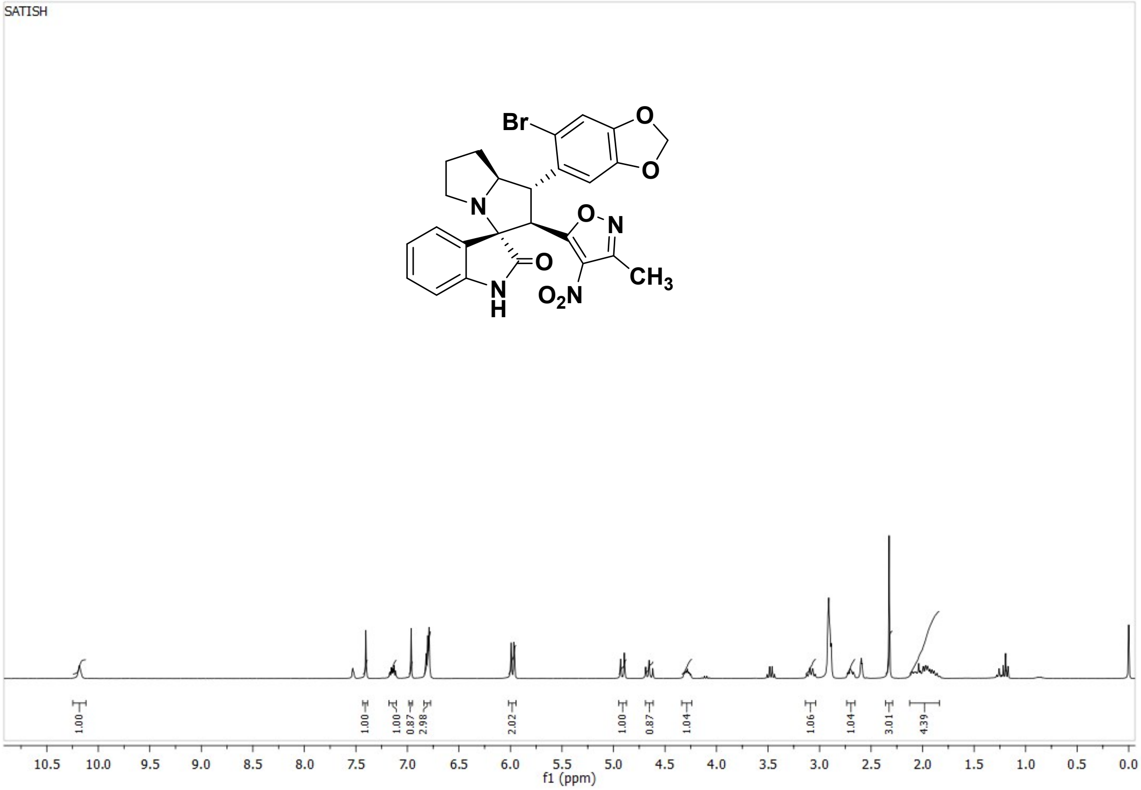

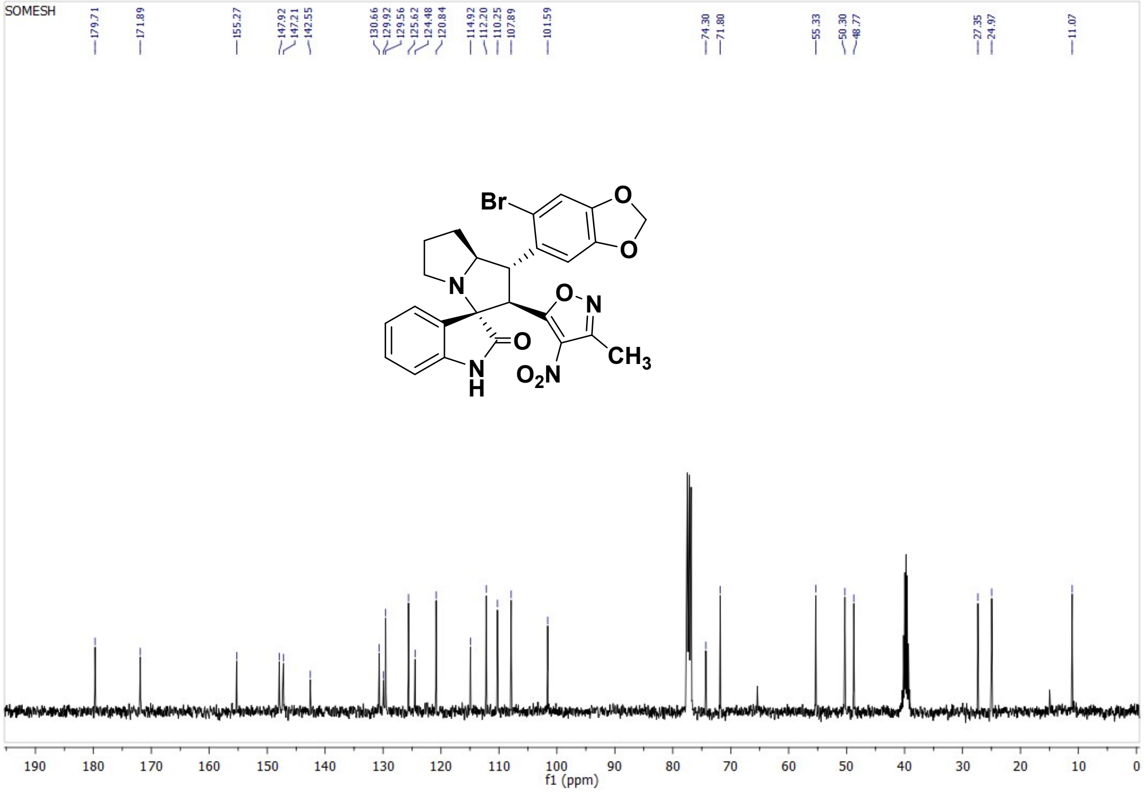

^{1}H &[13]C Spectra of 6f

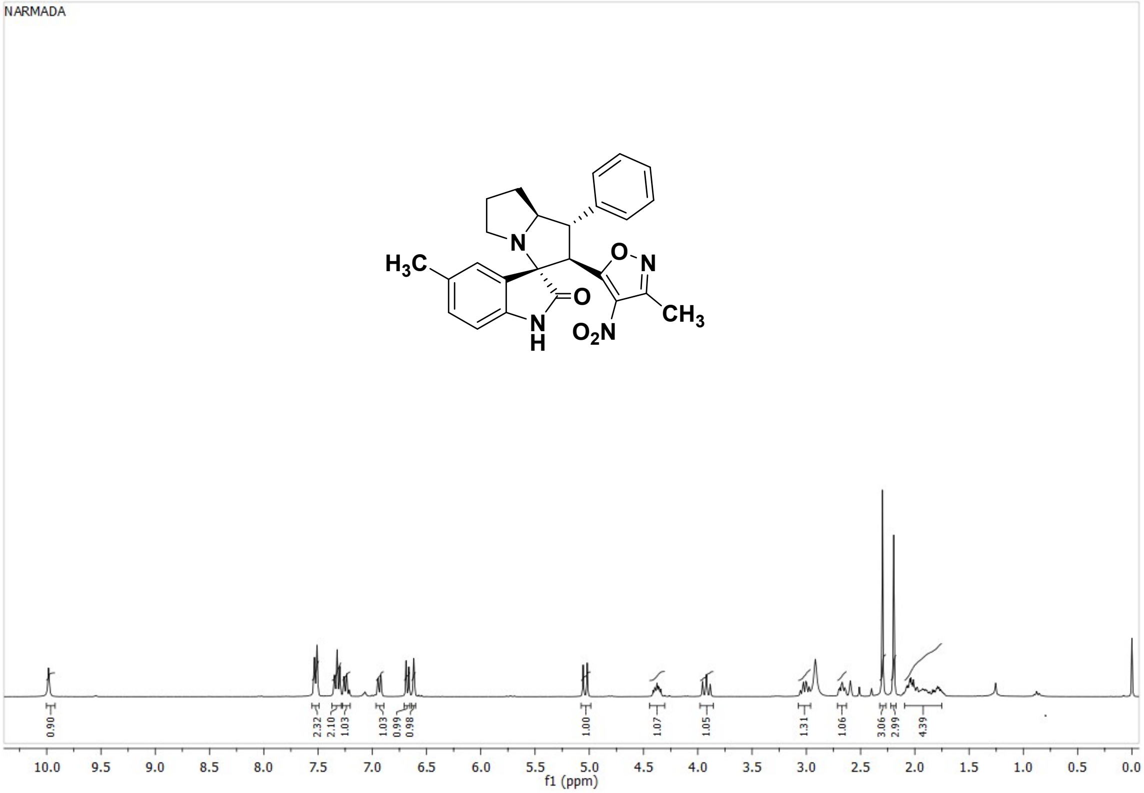

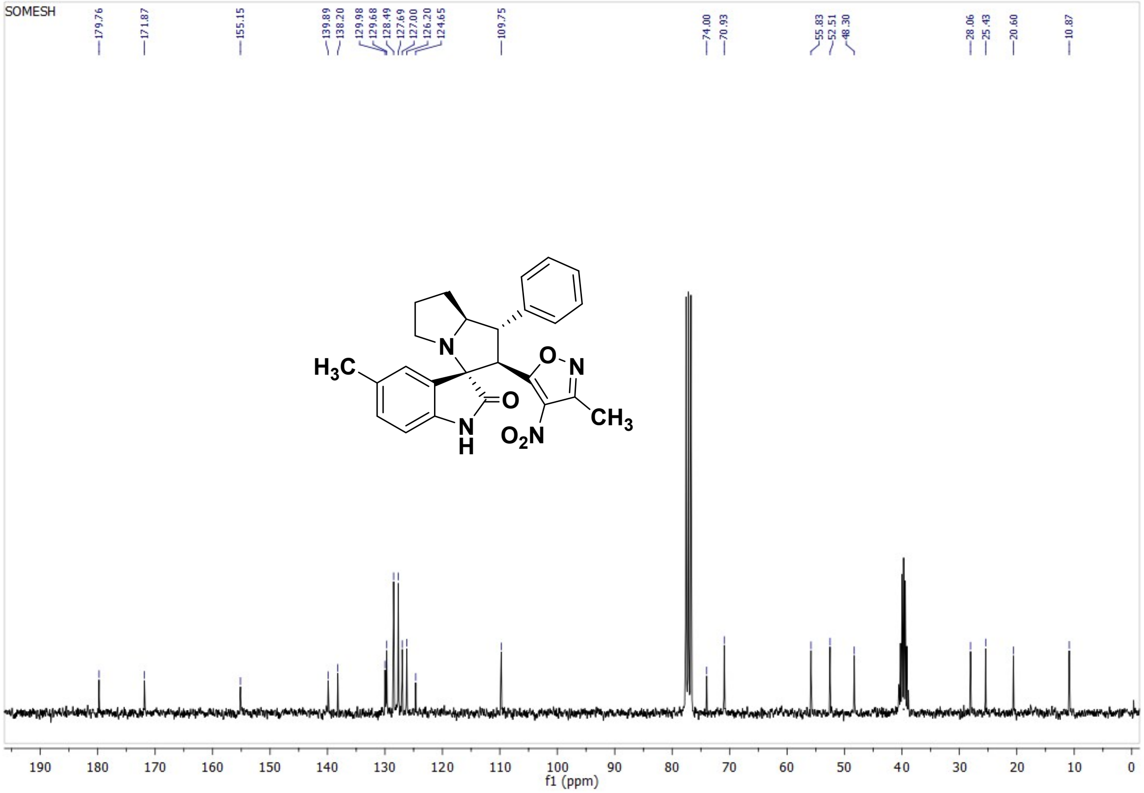

^{1}H &[13]C Spectra of 6g

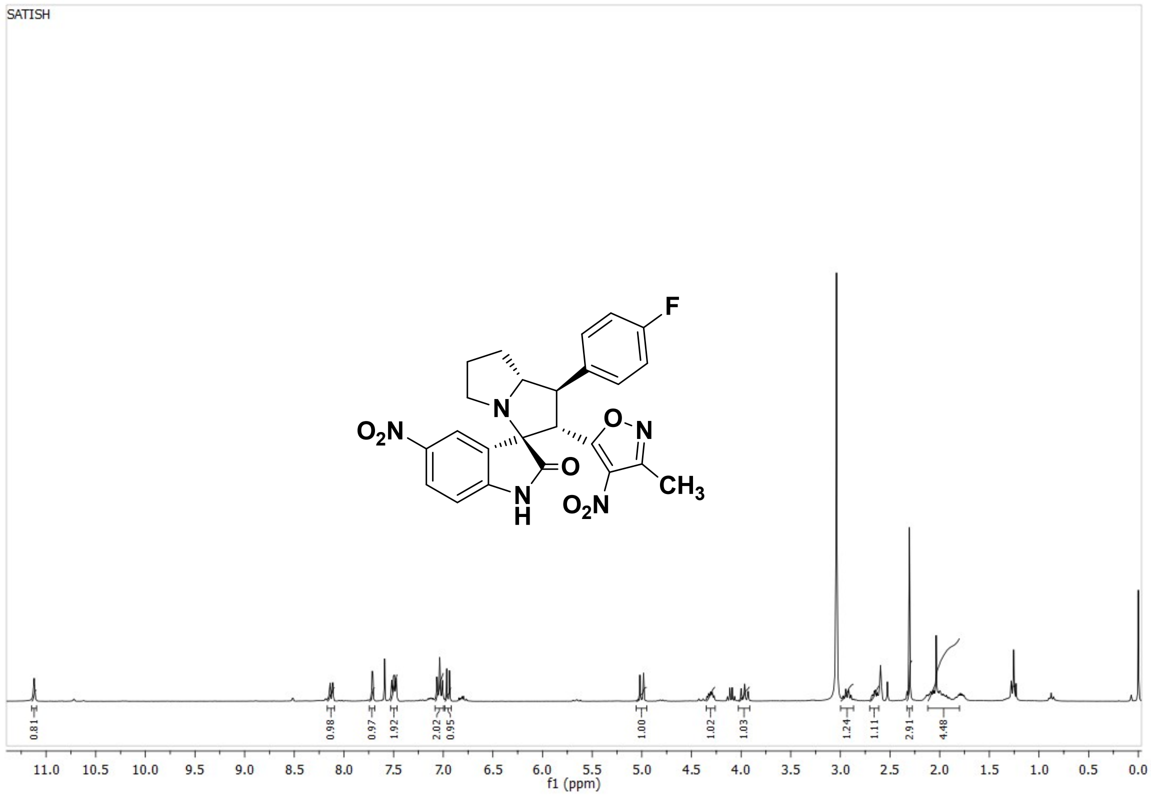

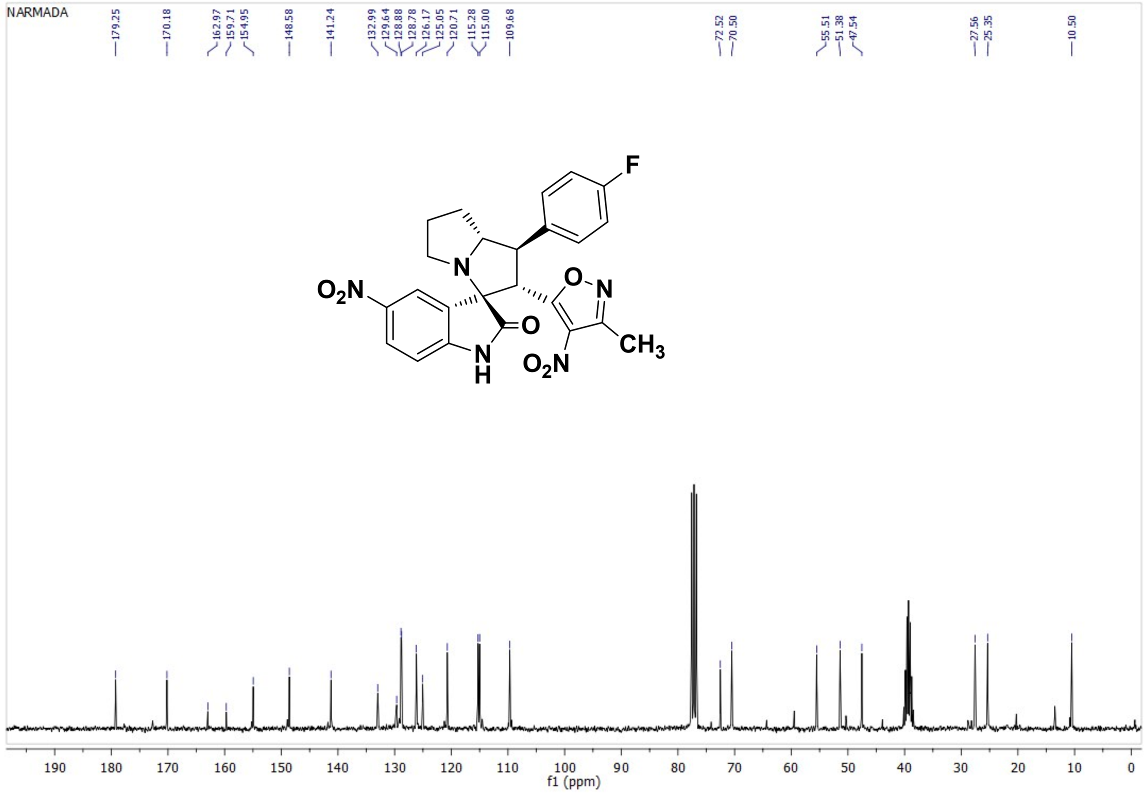

^{1}H &[13]C Spectra of 6h

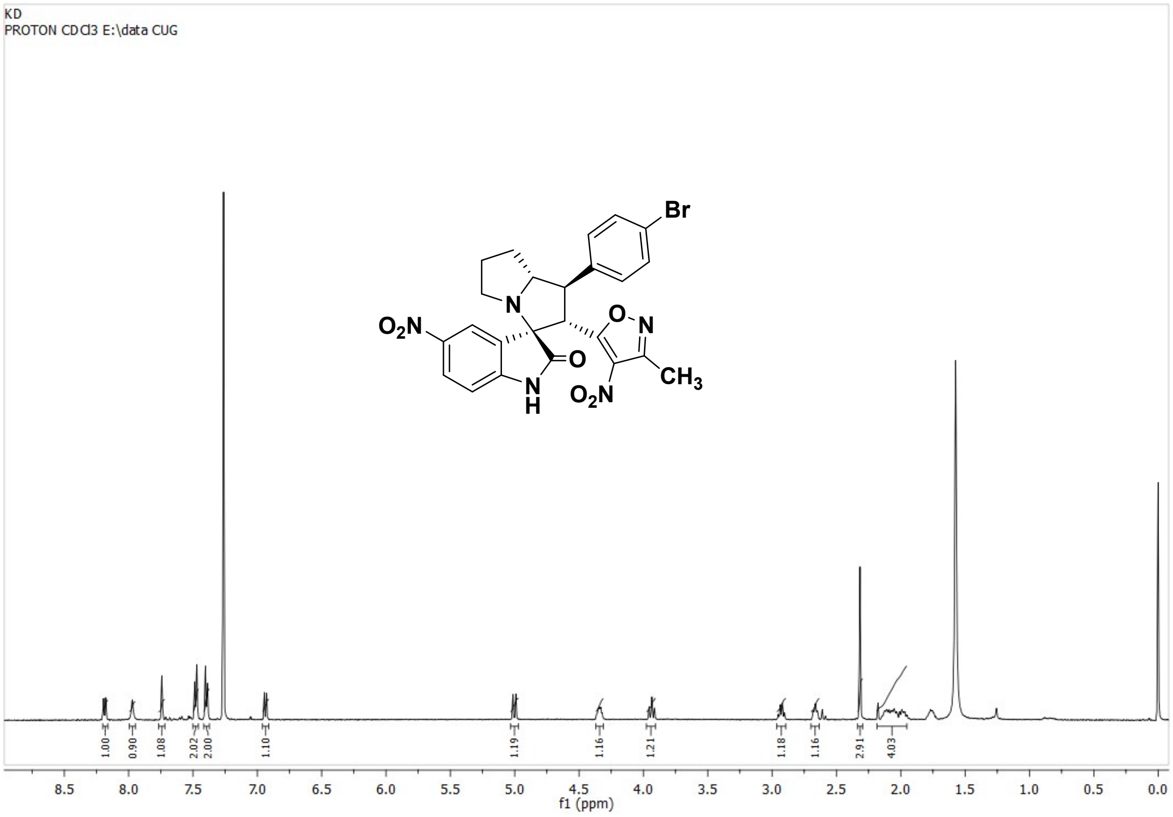

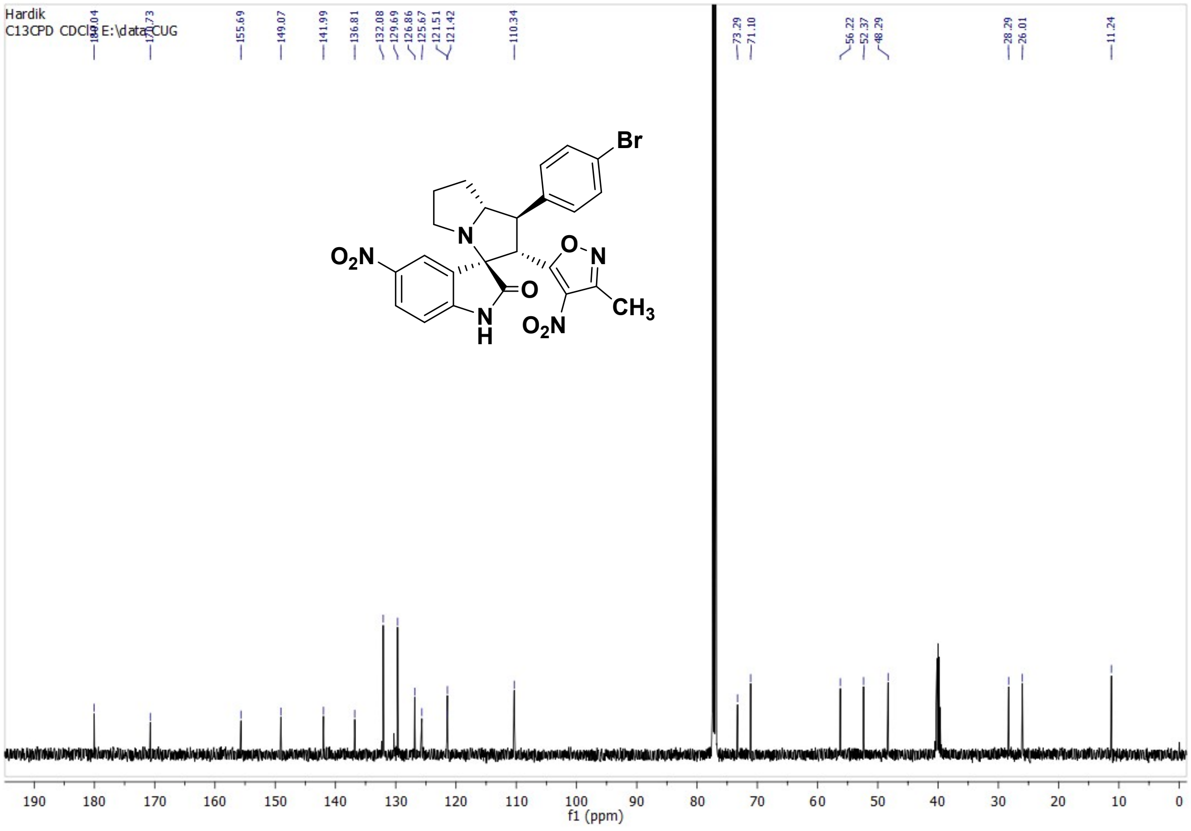

^{1}H &^{13}C Spectra of 6i

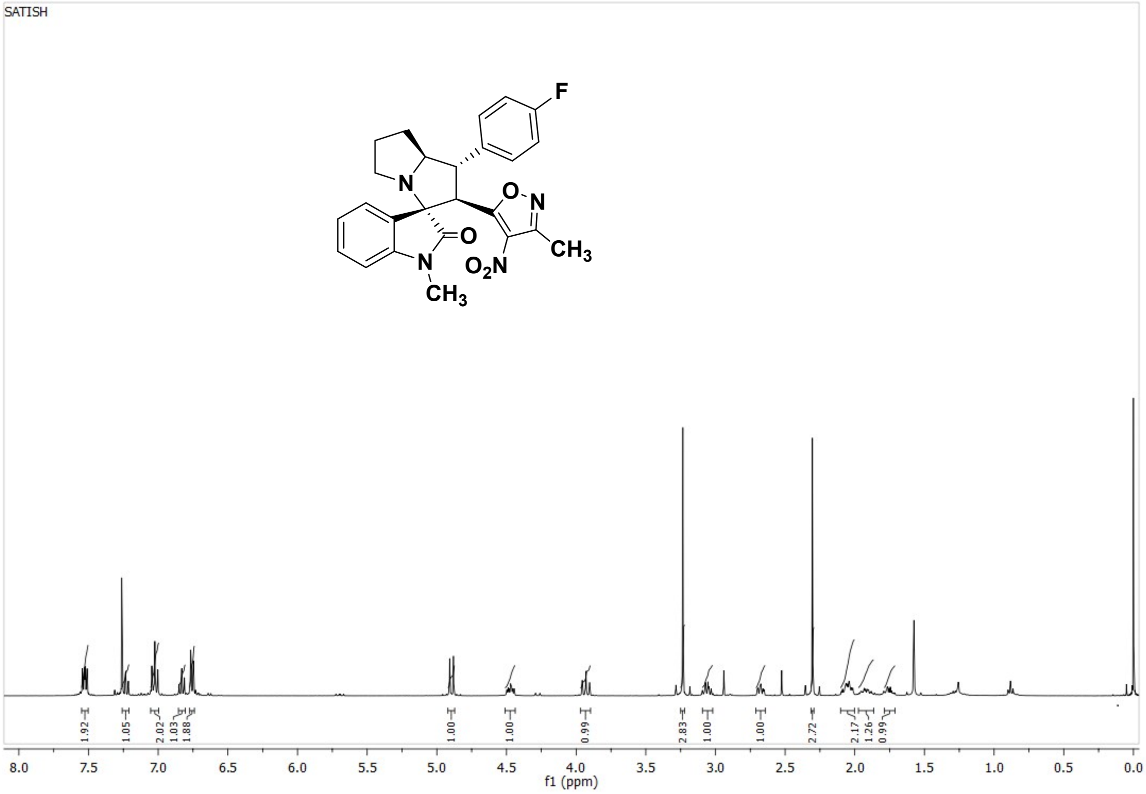

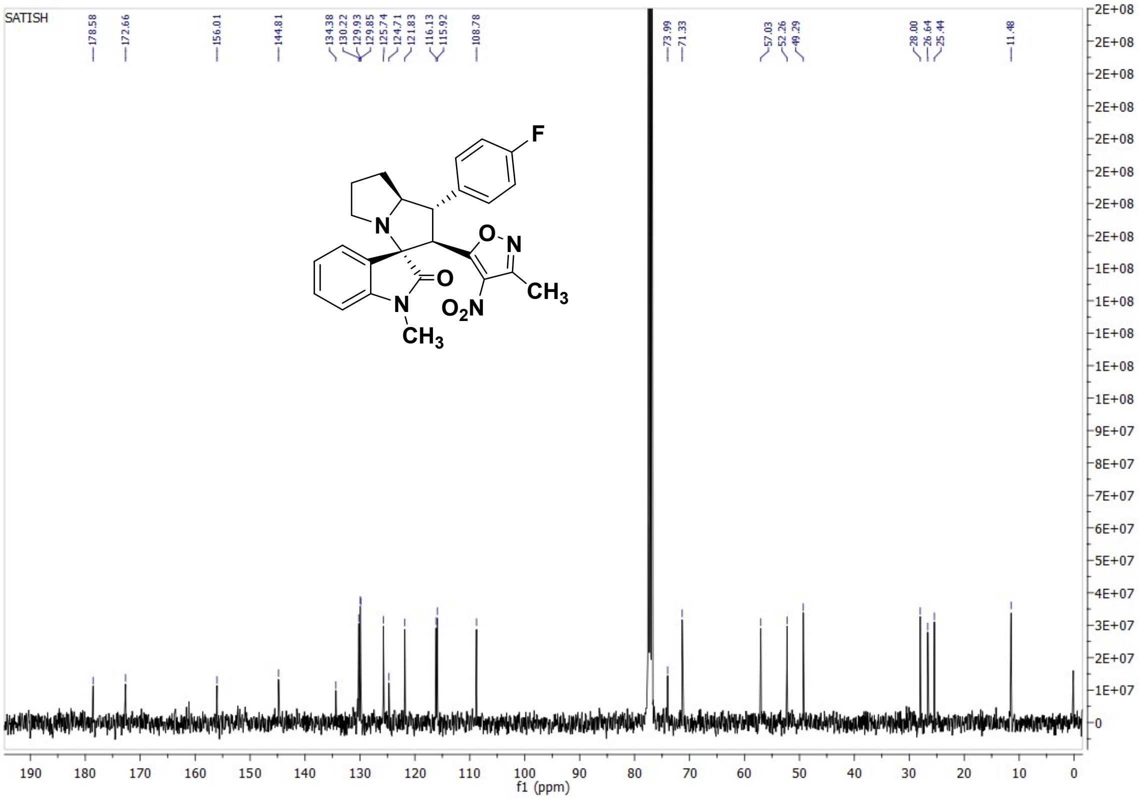

^{1}H & ^{13}C Spectra of 6j

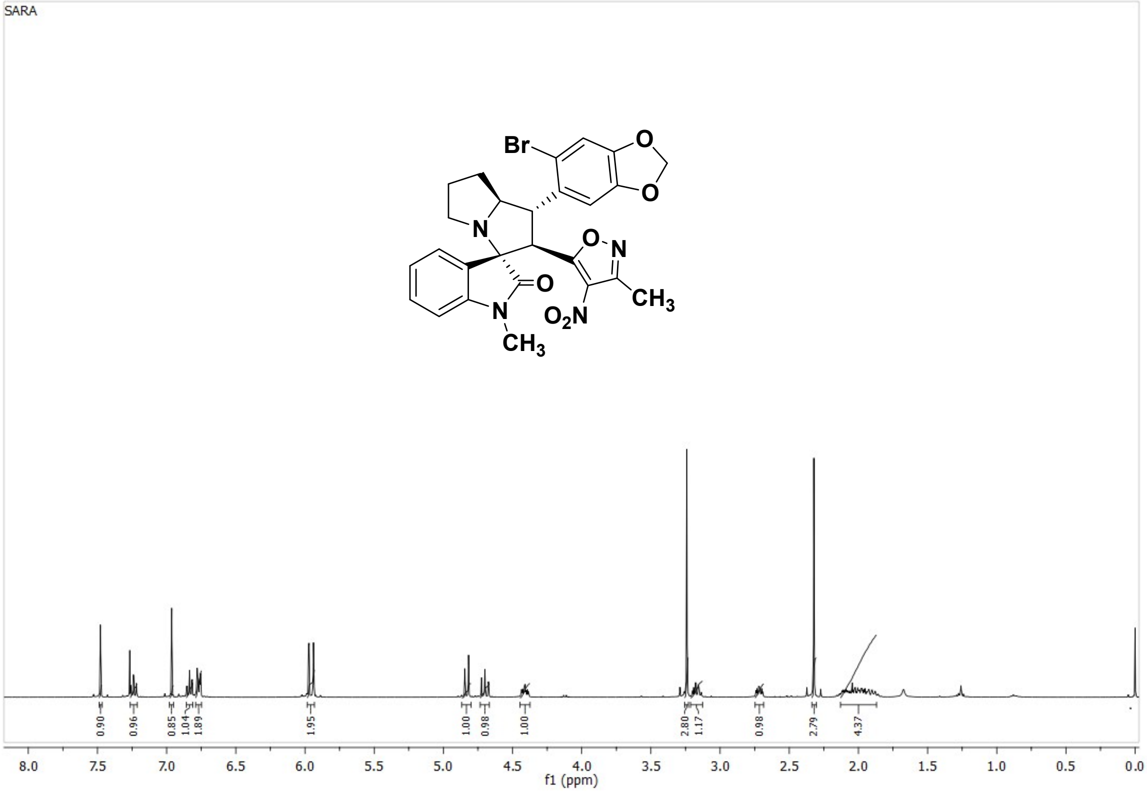

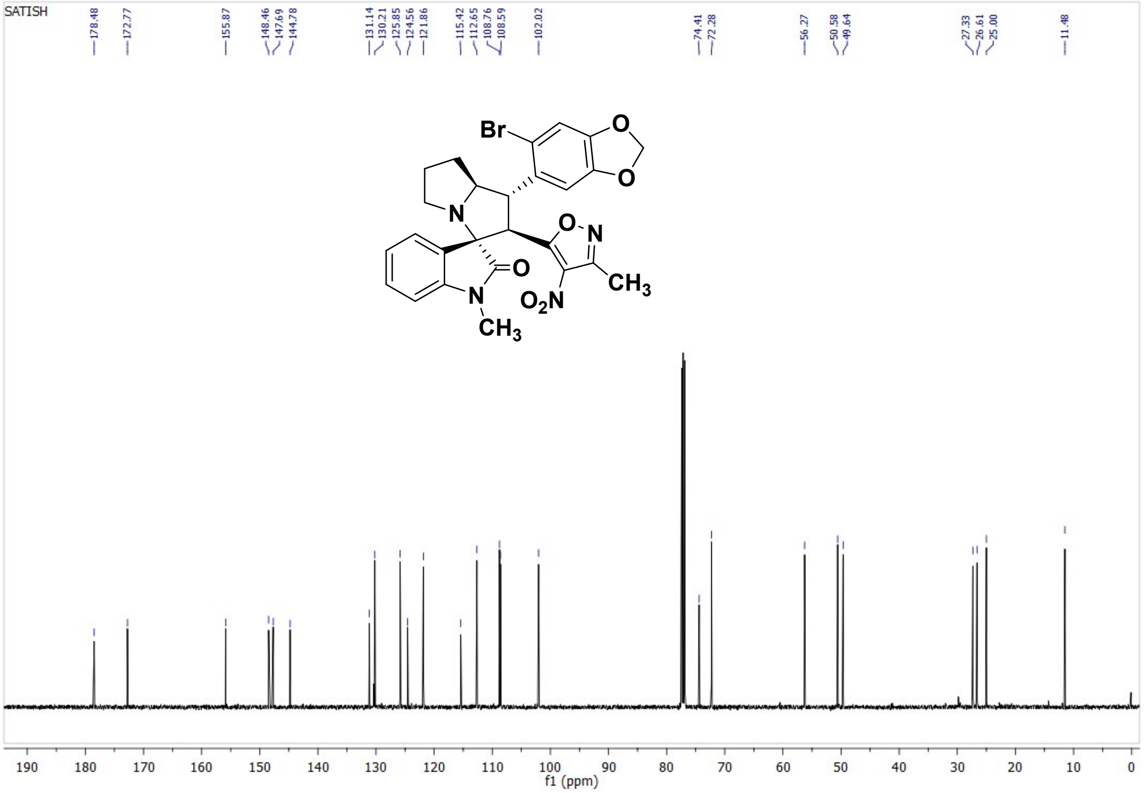

^1H & ^{13}C Spectra of 6k

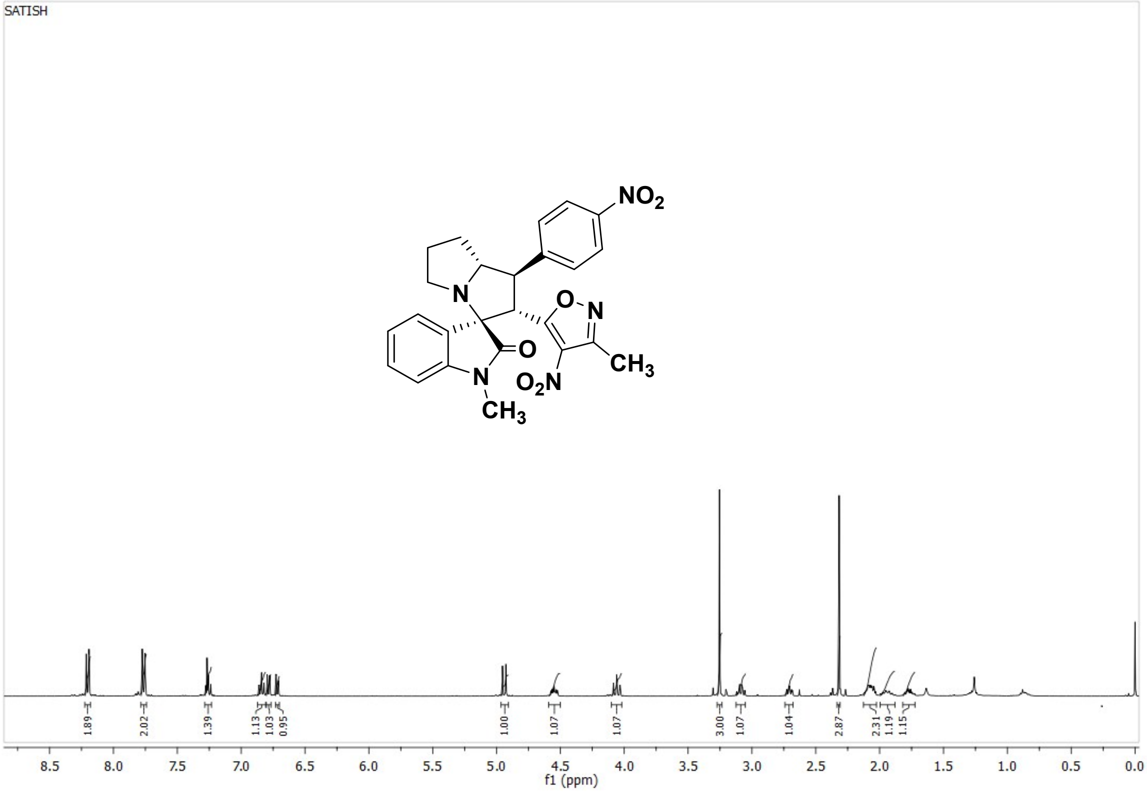

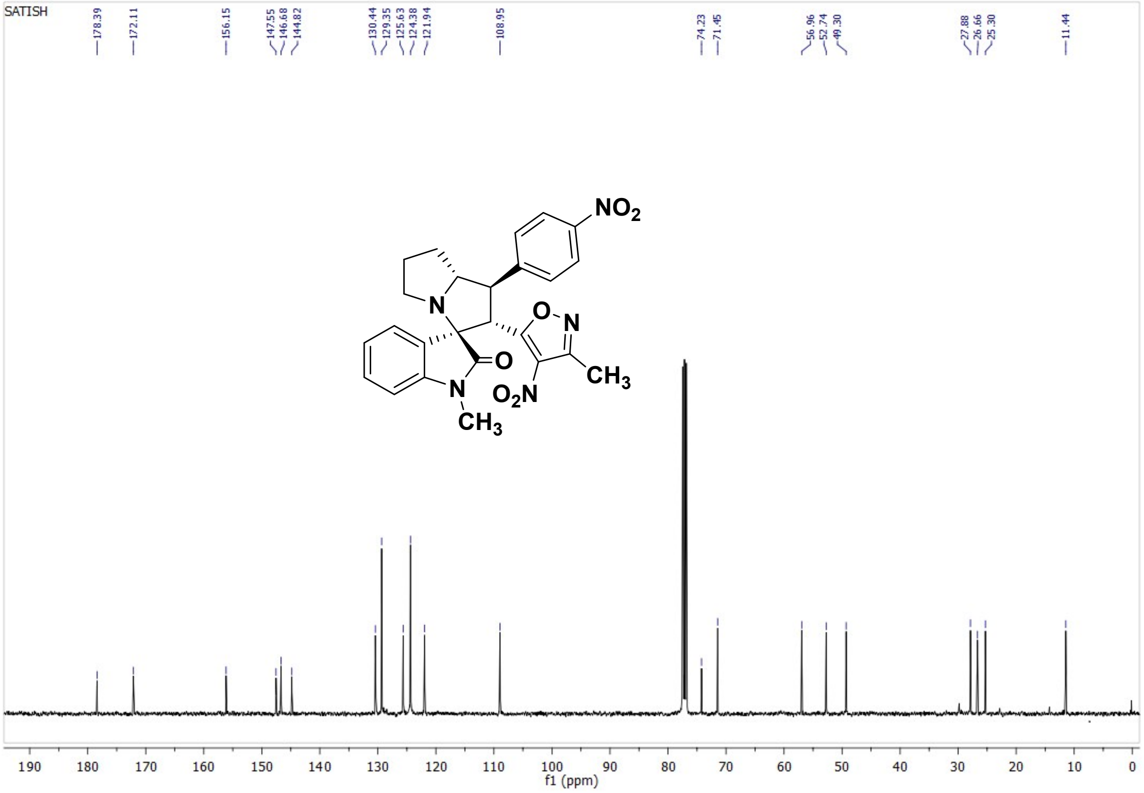

^{1}H & ^{13}C Spectra of 6l

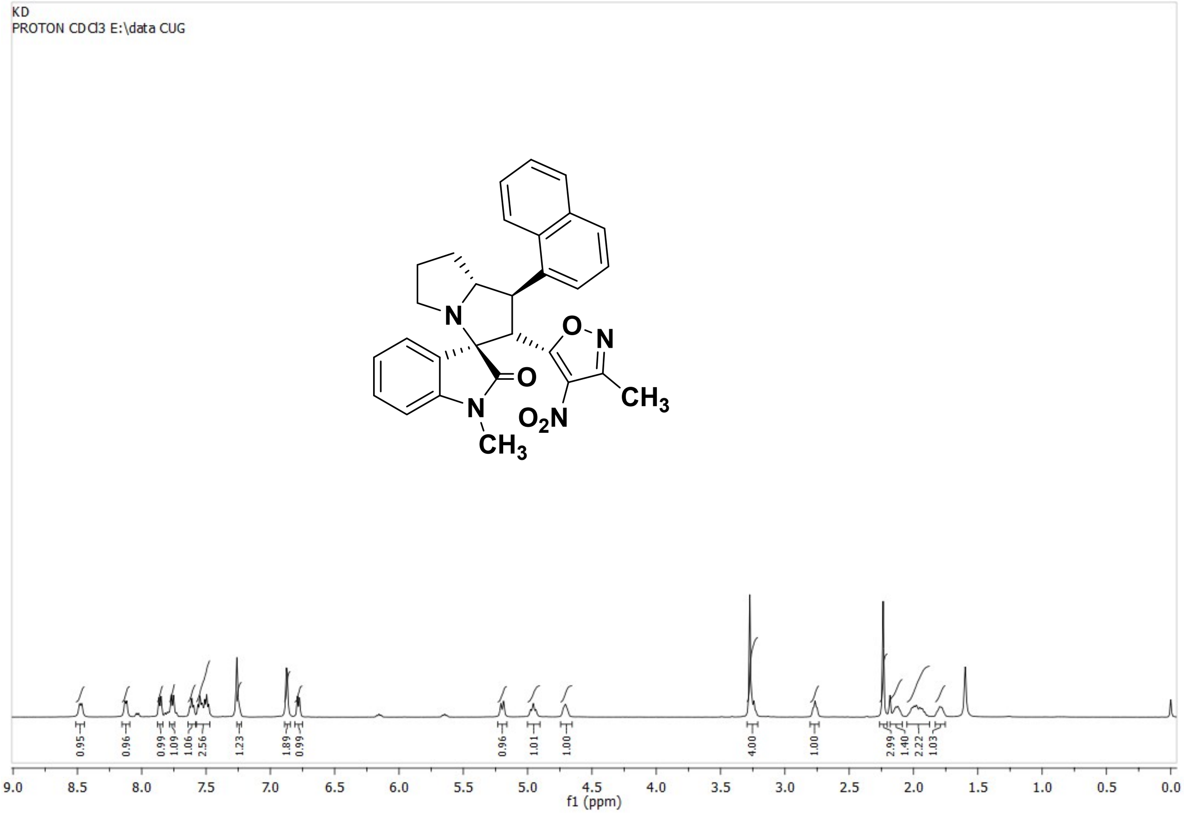

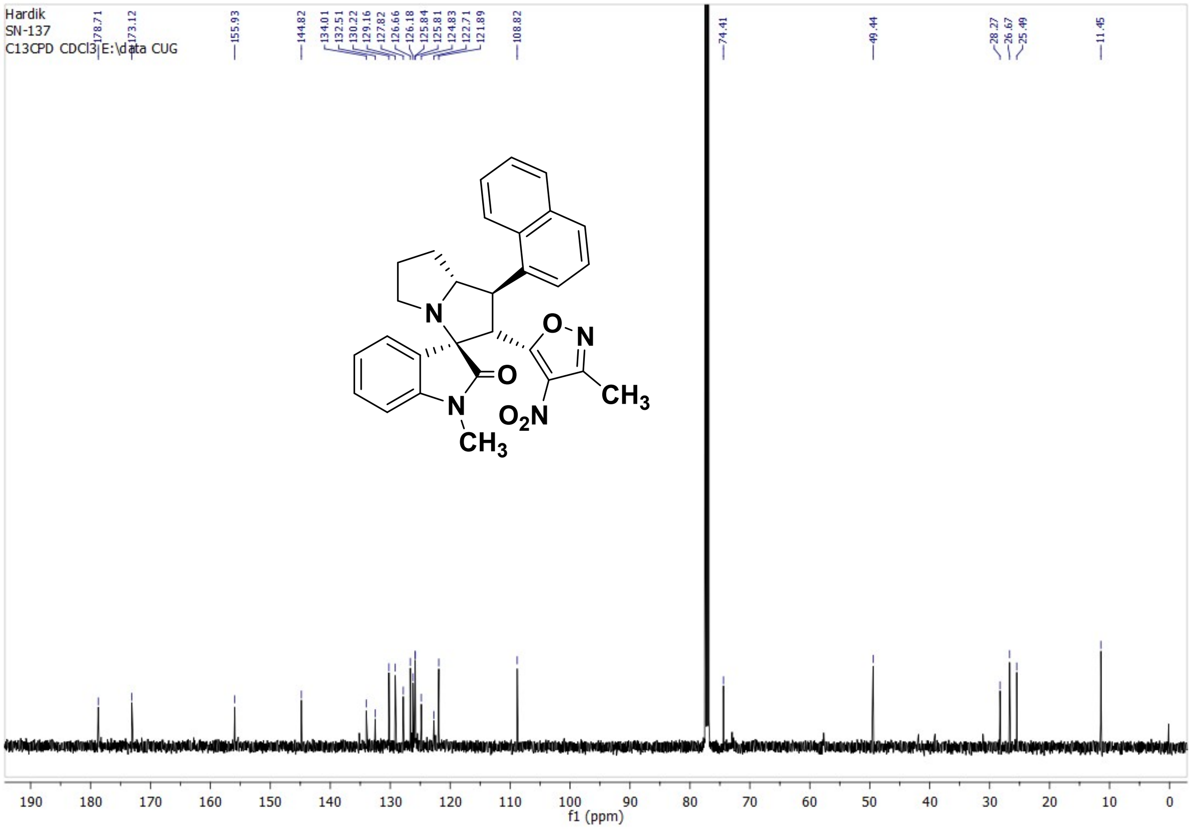

^{1}H & ^{13}C Spectra of 6m

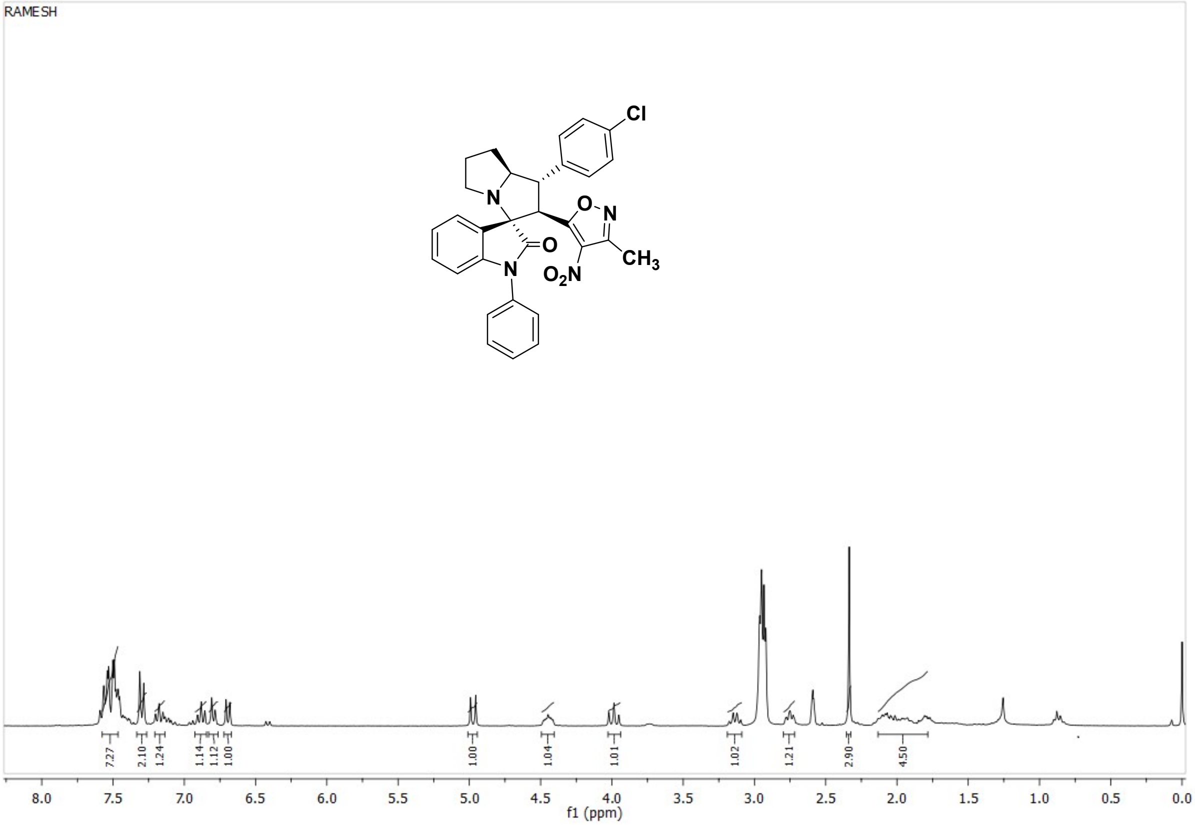

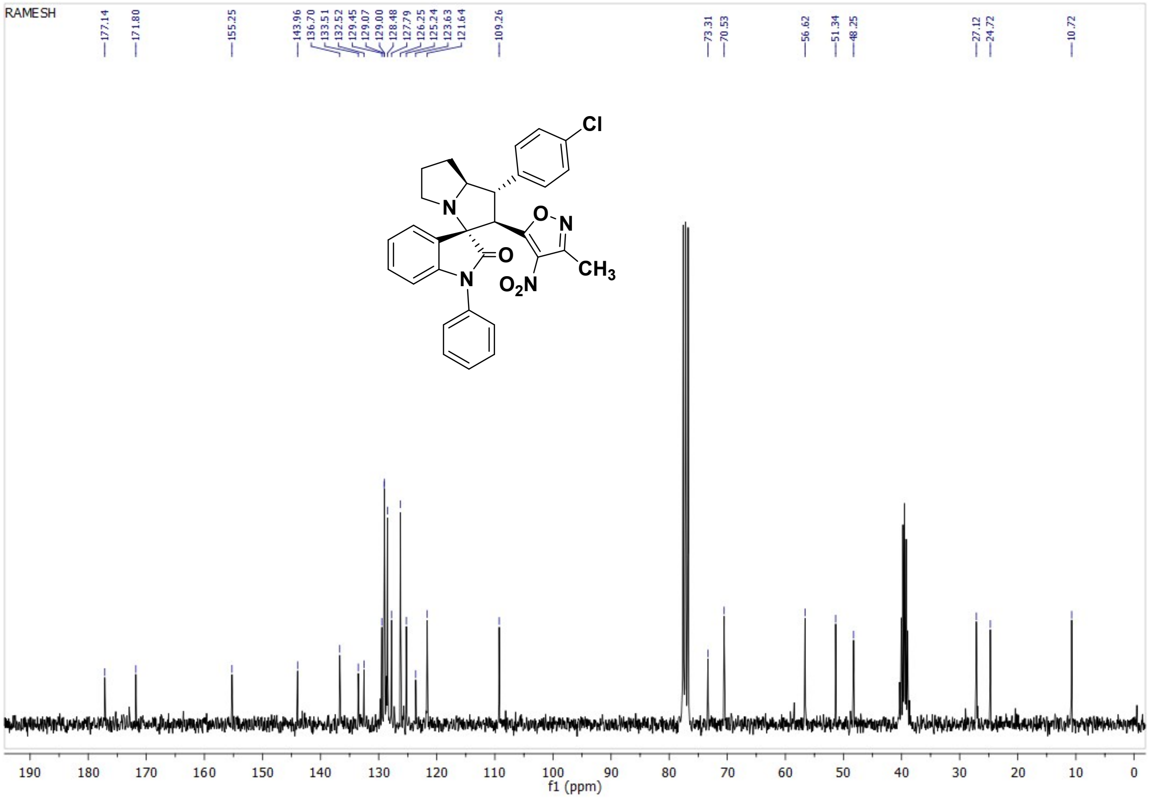

^{1}H & ^{13}C Spectra of 6n

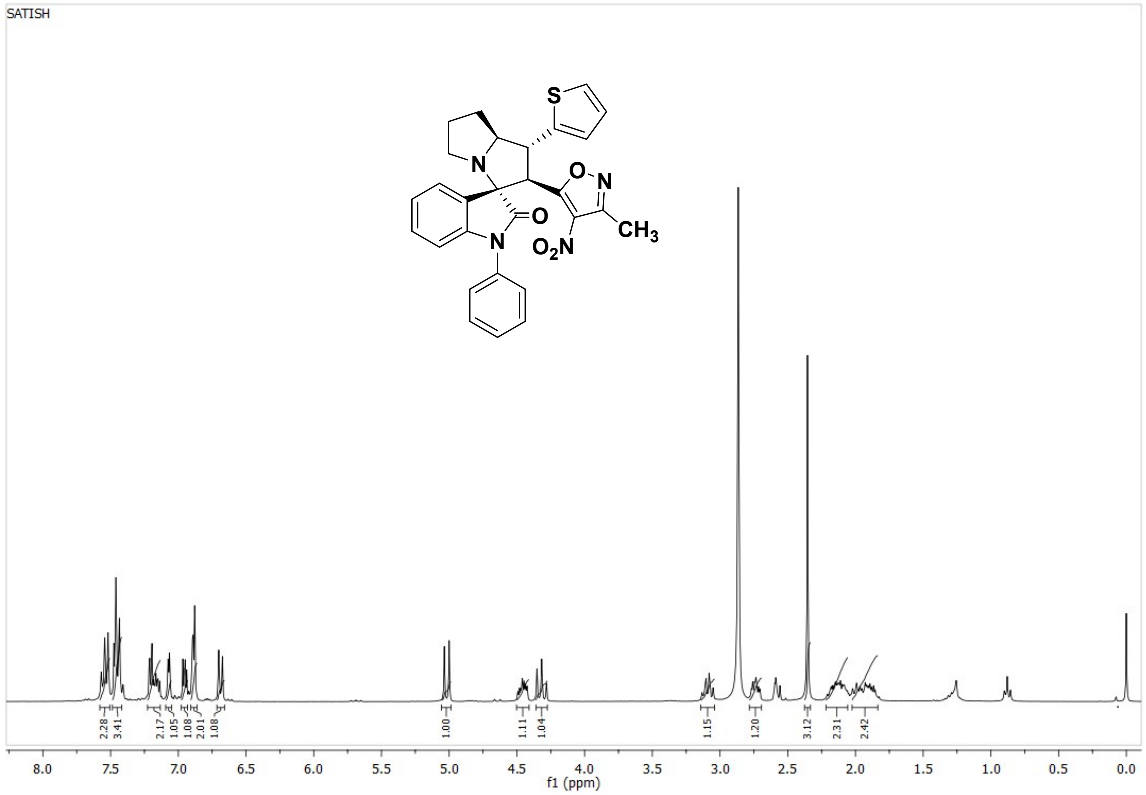

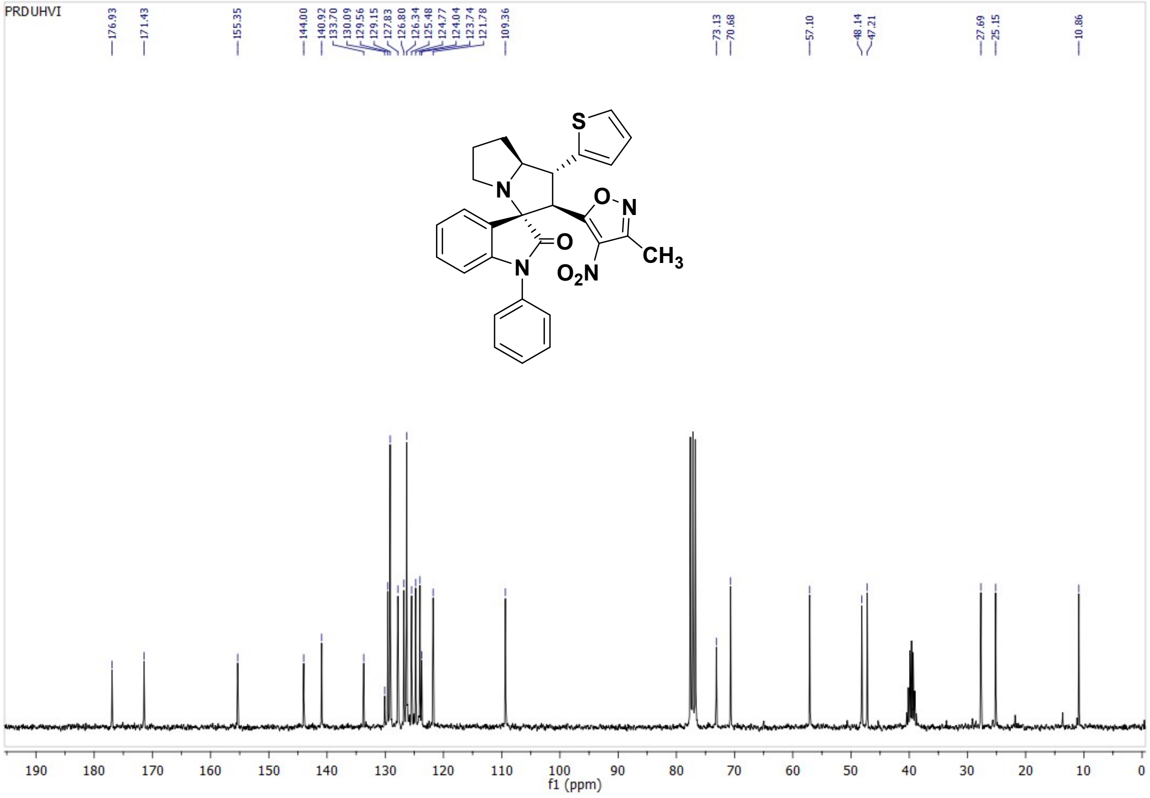

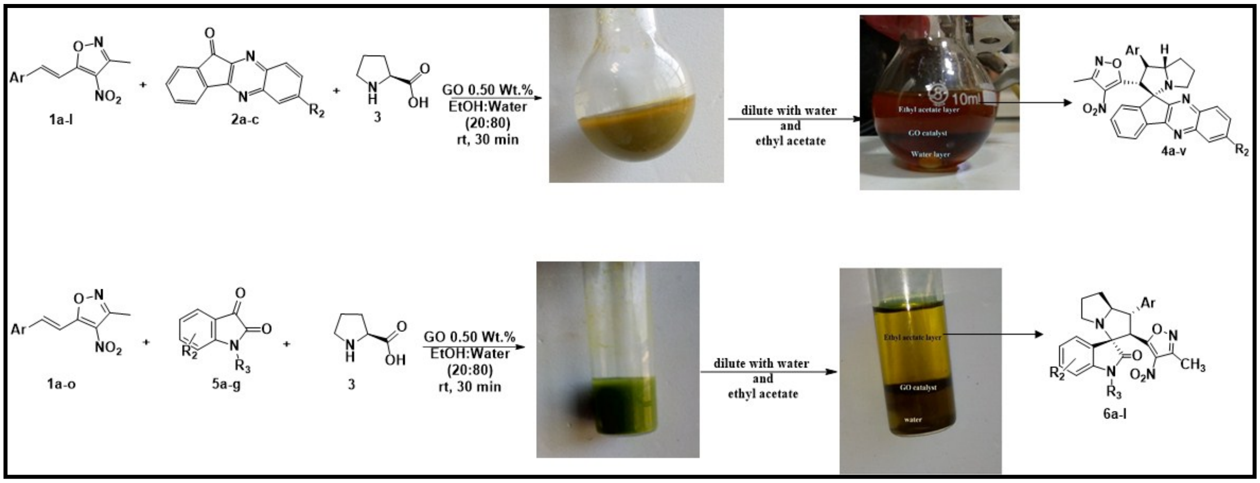
1a-l
2a-c
3
GO 0.50 Wt.%
EtOH:Water
(20:80)
rt, 30 min
dilute with water
and
ethyl acetate
Ethyl acetate layer
GO catalyst
Water layer
4a-v
1a-o
5a-g
3
GO 0.50 Wt.%
EtOH:Water
(20:80)
rt, 30 min
dilute with water
and
ethyl acetate
Ethyl acetate layer
GO catalyst
water
6a-l
